# Health care costs of case management for frequent users of the emergency department: Hospital and insurance perspectives

**DOI:** 10.1371/journal.pone.0199691

**Published:** 2018-09-24

**Authors:** Karine Moschetti, Katia Iglesias, Stéphanie Baggio, Venetia Velonaki, Olivier Hugli, Bernard Burnand, Jean-Bernard Daeppen, Jean-Blaise Wasserfallen, Patrick Bodenmann

**Affiliations:** 1 Institute of Social and Preventive Medicine (IUMSP), Lausanne University Hospital, Lausanne, Switzerland; 2 Technology Assessment Unit, Lausanne University Hospital, Lausanne, Switzerland; 3 IEMS Plateforme interfacultaire en économie et management de la santé, University of Lausanne, Lausanne Switzerland; 4 School of Health Sciences (HEdS-FR), University of Applied Sciences Western Switzerland (HES-SO), Fribourg, Switzerland; 5 Life Course and Social Inequality Research Center, University of Lausanne, Lausanne, Switzerland; 6 Department of Community Medicine and Public Health, Lausanne University Hospital, Lausanne, Switzerland; 7 Emergency Department, Lausanne University Hospital, Lausanne, Switzerland; 8 Alcohol Treatment Center, Lausanne University Hospital, Lausanne, Switzerland; 9 Vulnerable Population Center, Department of Ambulatory Care and Community Medicine, University of Lausanne, Lausanne, Switzerland; TNO, NETHERLANDS

## Abstract

**Background:**

In most emergency departments (EDs), few patients account for a relatively high number of ED visits. To improve the management of these patients, the university hospital of Lausanne, Switzerland, implemented an interdisciplinary case management (CM) intervention. This study examined whether the CM intervention—compared with standard care (SC) in the ED—reduced costs generated by frequent ED users, not only from the hospital perspective, but also from the third-party payer perspective, that is, from a broader perspective that takes into account the costs of health care services used outside the hospital offering the intervention.

**Methods:**

In this randomized controlled trial, 250 frequent ED users (>5 visits during the previous 12 months) were allocated to either the CM or the SC group and followed up for 12 months. Cost data were obtained from the hospital’s analytical accounting system for the entire sample and from health insurance companies for a subgroup (n = 140). Descriptive statistics and multivariate regressions were used to make comparisons between groups and assess the contribution of patient characteristics to the main cost components.

**Results:**

At the end of the 12-month follow-up, 115 patients were in the CM group and 115 in the SC group (20 had died). Despite differences in economic costs between patients in the CM intervention and the SC groups, our results do not show any statistically significant reduction in costs associated with the intervention, either for the hospital that housed the intervention or for the third-party payer. Frequent ED users were big users of health services provided by both the hospital and community-based services, with 40% of costs generated outside the hospital that housed the intervention. Higher age, Swiss citizenship, and having social difficulty increased costs significantly.

**Conclusions:**

As the role of the CM team is to guide patients through the entire care process, the intervention location is not limited to the hospital but often extends into the community.

## 1. Introduction

In many communities, although most patients visit hospital emergency departments (EDs) occasionally, a small number of patients generate a disproportionate proportion of total attendances. Depending on the definition, frequent users represent between 1 and 10% of the patients seen in the ED and may account for up to 20–25% of annual visits [1, 2]. In Switzerland, frequent ED users accounted for 4.4% of all ED patients and represented 12.1% of all ED visits at the Lausanne University Hospital in 2008–2009 [3].

Implications of this intensive use of hospital EDs have become a priority for clinicians, hospital administrators, and policy makers. ED overcrowding may put pressure on medical staff and budgetary resources, increasing waiting times and negatively affecting quality of care and patient outcomes. A substantial share of ED visits are related to nonemergent conditions, or issues that could be prevented or more appropriately managed in primary care or community settings. This inefficient use of costly emergency care reduces the system-wide capacity to handle “real” emergencies, hinders the intake and management of the entire pool of patients, and contributes to the increase in overall health care expenditures.

Several interventions have aimed at managing frequent ED users more efficiently. Case management (CM) is the most common type of such an intervention and has been implemented in many countries for frequent ED users [4, 5], as well as for other groups with specific diseases [6–12]. CM intervention consists of an interdisciplinary approach that assesses, plans, personalizes, and guides the use of individual health service resources, at the same time coordinating them, to improve patient outcomes and reduce use of health resources and their associated costs.

Several studies that targeted ED frequent users, including randomized controlled trials (RCTs) and nonrandom comparative cohort designs, showed that CM interventions were associated with improvement in patient satisfaction or quality of life [13, 14], as well as a reduction in the mean or median number of ED visits [13, 15–19]. Other recent studies showed that CM intervention appeared to be effective in reducing ED visits, but without reaching statistical significance [20, 21]. In addition, CM interventions had an impact on costs, although the studies used heterogeneous methods regarding cost outcomes and cost calculations. Considering the perspective of the third-party payer (i.e., the insurer), studies reported reductions in ED charges [15, 22], inpatient charges [16, 22], and combined ED and inpatient charges [23] following the implementation of the intervention. Similarly, but from a hospital perspective, Shumway et al. [13] reported that a group that received the CM intervention had lower ED costs and Murphy and Neven [17] reported that such a group had lower total treatment costs.

In these CM intervention cost analyses for ED frequent users, whatever the perspective adopted, the outcomes of interest included only the costs of services provided in the ED or within the hospital where the intervention was conducted. The investigators did not examine the costs generated by the use of services provided in other local hospitals or in independent primary care practices. One of the main objectives of CM interventions, however, is to limit the need for costly ED visits by preventing the exacerbation of existing conditions and reducing the incidence of severe acute events. This is done by reorienting patients toward community-based services and thus increasing their access to more appropriate (and potentially less costly) primary care. Although some of these services may be delivered through ambulatory consultations at the hospital that implements the CM intervention, they may also be offered by primary care practices in the community. The costs of all of these substitute services should be taken into account when conducting cost analyses of CM interventions to assess their broader impact.

Thus, using data from an RCT comparing a CM intervention with standard care (SC) among frequent ED users of the Lausanne University Hospital, Switzerland, we examined the potential of such an intervention to reduce the costs of health care services from two different perspectives. First, a cost analysis was conducted from the perspective of the hospital that housed the intervention by appraising different health services used within its facilities. Second, a broader analysis was done by examining the costs of health services used, both within and outside the hospital that offered the intervention, from a third-party payer perspective. To our knowledge, this study is the first to use an RCT design to investigate the impact of CM on health care costs with a larger scope than that of the hospital implementing the intervention.

## 2. Methods

### 2.1. Setting

In Switzerland, access to health care is ensured through a universal health coverage system. Since 1996, the Health Insurance Law has imposed mandatory health insurance (MHI) on all Swiss residents, who are required to buy individual health insurance from one of the 53 competing private insurance companies on the market. The dynamics of the market, ruled by the Health Insurance Law, relies on 4 main principles: (1) Health insurers cannot make profits on contracts for MHI, (2) consumers have free choice of their insurer, (3) insurers cannot refuse or select individuals for MHI because of preexisting conditions or risk factors and are compelled to accept any applicant, and (4) MHI covers a standard benefits package regulated by federal legislation and including a comprehensive range of outpatient and inpatient care and services [24].

Health coverage is financed through uniform per capita premiums paid to insurance companies. Patients pay a deductible that they select from a range of CHF 300 to 2,500 and an annual participation fee that includes a copayment for health services used (10%). Higher deductible levels are associated with a reduction in premiums. The level of cost sharing (deductible, coinsurance of 10% up to an annual ceiling) is defined by law and is identical across insurers. Subsidies are given to people with low incomes, so that almost the entire population is covered by MHI [24]. In addition, patients are able to enroll in supplementary and complementary health insurance at an extra cost from the same or a different insurer on a voluntary basis.

The third-party payer system, applicable to almost all companies and for all types of health care services (outpatient, inpatient, medications), implies that the price of health care services used by patients is not paid by the user or the provider, but by the insurer. Medical invoices are sent to health insurance companies and are calculated on a fee-for-service basis for ambulatory physicians and outpatient services provided by hospitals, and on a diagnosis-related group payment system for inpatient care.

Funding of the health care system is split between different government levels and different social insurance schemes. Resources are collected through taxes and MHI premiums. MHI companies are the most important purchasers and payers in the system, financing 36% of total health expenditures in the country (in 2015). Out-of-pocket payments finance 28% of total health expenditures and government spending finances 18% [25]. Complementary and supplementary voluntary health insurance plays a small role, financing about 6% of total health expenditures. The balance in the system is kept because an increase in health expenditures observed in the previous year directly affects the premium amount paid by the enrollees the next year.

### 2.2. Data

We used data from an RCT comparing a CM intervention with SC among frequent ED users of the Lausanne University Hospital, an urban public hospital serving (with other non-university hospitals) a population of 770,000 located in the French-speaking part of Switzerland. It is one of the 5 teaching university hospitals in the country and provides primary care to the local population, as well as tertiary and highly specialized care to a larger population area. In 2013, it provided medical, surgical, and mental health care through 39,000 annual ED visits and 45,200 inpatient stays [26].

The trial recruited 250 adult frequent ED users (defined as 5 or more visits during the previous 12 months) who visited the ED between May 2012 and July 2013. The participants were randomly and equally allocated either to the group receiving the CM intervention (n = 125) or to the group receiving standard ED care (n = 125) and followed up over 12 months. The sample size was determined for the first outcome, an expected comparative reduction of 2 ED visits per year in the intervention group. The control group received SC through the ED, specialists, physicians, and nurses focusing on somatic and/or mental diseases and/or behavioral-specific acute problems. The treatment group, in addition to SC, received CM intervention conducted by an interdisciplinary team (4 nurses supervised by a general practitioner) whose role was to provide social and medical support to each individual. During the baseline meeting, the social situation, physical and mental health status, and risky behaviors of the patients were assessed by the team, leading to the elaboration of a personalized care program. The monitoring of this program was done during 3 other meetings with the team, at 1, 3, and 5 months later at the patients’ homes or in an ambulatory setting or on the telephone. In addition, the patients could contact, at any time, one of the members of the CM team. The primary goals of the CM team were to provide practical assistance and appropriate referrals depending on the situation. Thus, if necessary, social assistance in obtaining financial support, housing benefits in order to have stable housing, or educational opportunities were offered. Patients were also referred to mental health services, substance abuse management services, or a general practitioner or primary care provider, if appropriate. The role of the CM team was to facilitate communication between social and health care providers, both inside the hospital and in the community, in order to maintain continuity of care. More information on the trial is available from the published protocol [27].

The present study focused on costs of health care services used by patients and did not take into account the costs of social assistance (social services) provided to frequent users. Costs of health care services were assessed differently depending on the perspective adopted. For the cost analysis from the hospital perspective, the true value of the resources that were needed to provide health care services was used as a cost estimate. These data were obtained from the hospital’s analytical accounting system, which assigns monetary values to resources involved in providing health care to patients (costs of labor, pharmaceuticals and medical supplies, meals, linen and clothing, utilities, maintenance and repair of buildings and equipment, laundry, cleaning, office supplies, communication, etc.). Costs were available over 12 months following enrollment in the trial. To allow for comparisons, we computed average monthly hospital costs. Total hospital costs were split into 5 components, depending on the medical nature of health care services used: ambulatory costs, somatic inpatient costs, rehabilitation costs, psychiatric costs, and ED costs.

For the broader health care cost analysis, we used MHI claims data. These data were obtained from each insurance company that was individually contacted for each participant who had given his/her consent. The insurance companies that agreed to cooperate provided a unique cost estimate corresponding to the global sum of individual costs over the inclusion period of the study. Given the third-party payer system in Switzerland, these data represent a unique estimate of total health costs from the third-party payer perspective, hereafter referred to as total insurance costs. In addition, similar insurance cost data for health care services provided by Lausanne University Hospital were obtained from its invoicing department, which centralizes all invoices before sending them to health insurance companies for reimbursement. We then split total insurance costs into 2 groups: costs related to health services provided by Lausanne University Hospital (intramural insurance costs) and costs related to services provided by providers other than the hospital (extramural insurance costs). Thus, the costs of health services used within the hospital were assessed from 2 different perspectives, yielding different outcomes, which, because of their definitions, are difficult to compare directly.

For several reasons, including patient consent, cooperation of health insurance companies, and accuracy of information transmitted, insurance cost data were available for a subsample of participants only (n = 140, 56%; control n = 65, 52%; treatment n = 75, 60%). These data were available for people alive at the end of the study. Individual average total monthly insurance costs were calculated.

### 2.3. Statistical analyses

The analyses were performed according to the intention-to-treat principle. Descriptive statistics were computed by using means, median for continuous variables, absolute frequencies, and percentages for categorical variables. Statistical comparisons between the control and treatment groups were performed with the Student's *t*-test for age and chi-squared tests for categorical variables. Differences in costs across treatment and control groups were assessed with unadjusted regressions.

Because of the skewed and heavy-tailed nature of cost data, we used alternative regression methods that have been widely tested in the econometric literature [28–31], although no single approach emerges as optimal [32]. Generalized linear models (GLMs) offer several advantages in this context [33, 34]. We used gamma GLMs with a log link that performed better for modeling the variables of costs and are commonly used with health care expenditures [35, 36]. The log link allowed us to interpret exponentiated coefficients easily as the multiplicative effect on the outcome of a unit change in the regressor.

We also analyzed the variation in individual costs by using multivariate regressions to assess the contribution of patient characteristics to the main cost components from the hospital perspective (total costs, ambulatory costs, ED costs) and from the insurance perspective (total, intramural and extramural costs). These analyses allowed us to better identify the aspects of the lives of frequent ED users in order to focus on the context of CM interventions. We included, as covariates, patient *group* (variable took a value of 1 if the patient was in the treatment group; 0 otherwise), *gender* (1 for men; 0 otherwise), *Swiss citizenship* (1 if the patient had Swiss citizenship; 0 otherwise), whether *the patient died during follow-up* (1 if the patient died; 0 otherwise) and *age* (considered as a continuous variable). In addition, 4 other binary variables capturing *social difficulties*, *somatic conditions*, *mental health problems*, *and risky behaviors* were included. The variable *social difficulty* took a value of 1 if the patient presented at least one of the following: a complex family situation, social isolation, financial hardship, inadequate housing, lack of employment or other activities, problems with immigration status, or limited French proficiency; it took a value of 0 otherwise. *Somatic condition* took a value 1 if the patient presented at least one of the following: severe chronic and/or acute illness, comorbidity, polypharmacy, treatment nonadherence, or physical handicap; it took a value of 0 otherwise. Similarly, *mental health problem* took a value of 1 with at least one of the following diagnoses: depression, anxiety, or personality and psychotic disorders; it took a value of 0 otherwise. Finally, presenting *a risky health behavior* referred to any of the following diagnoses: alcohol abuse problem, illicit drug use, tobacco use, or game addiction. In addition, the impact of *having a primary care physician* (value of 1) or not (0) was tested.

The results of unadjusted and multivariate regressions report the exponentiated coefficients, namely, the relative risks (RRs) with 95% confidence intervals (CIs) and *p* values for gamma-log GLMs for each cost outcome. Statistical analyses were performed with STATA software (version 14; StataCorp LP, College Station, TX, USA).

## 3. Results

Baseline characteristics of the 250 patients in the trial are presented in Table 1. There were no differences between the treatment and control groups in terms of demographic characteristics (gender and age), determinants of health (social difficulty, somatic and mental health problem, and risky behavior), and having a primary care physician, given the random allocation across treatment and control groups. Twenty patients, equally distributed between the 2 groups, died after 5.4 months of follow-up on average (same for both groups). More information about the patients who died during the follow-up period has been published elsewhere (see [37]).

**Table 1:** Demographic characteristics and determinants of health in the sample studied.

	Whole sample (n = 250)	Control group (n = 125)	Treatment group (n = 125)
Gender *Female* *Male*	107 (42.8) 143 (57.2)	52 (41.6) 73 (58.4)	55 (44.0) 70 (56.0)
Age, mean (SD)	46.1 (18.9)	46.3 (19.2)	46.0 (18.6)
Swiss citizenship	119 (47.6)	61 (48.8)	58 (46.4)
Social difficulty	182 (72.8)	89 (71.2)	93 (74.4)
Somatic condition	173 (69.2)	83 (66.4)	90 (72.0)
Mental health problem	126 (50.4)	64 (51.2)	62 (49.6)
Risky behavior	70 (28.0)	32 (25.6)	38 (30.4)
Not having a primary care physician	35 (14.0)	15 (12.0)	20 (16.0)

**Notes**: All data are reported as number (%), except where otherwise indicated.

Table 2 presents data on hospital costs for health care services provided by the hospital for the entire sample and for the 2 treatment groups. Overall, most patients (248/250) used health care delivered by the hospital during follow-up, yielding an average monthly cost of CHF 3,754 for the hospital. On average, somatic acute hospitalizations represented 65% of total hospital costs; 60% of patients (61% in the treatment group and 58% in the control group) had at least one acute inpatient stay. Almost all patients (97%) had at least one ambulatory visit to the hospital, with ambulatory care amounting to CHF 718 per month on average. Although psychiatric care accounted for 11% of hospital costs on average, only 35 of 250 participants used this type of service (21 in the control group and 14 in the treatment group) during the 12 months of follow-up.

**Table 2:** Cost data from the hospital perspective for the whole sample, the control group, and the treatment group.

Hospital perspective monthly costs	Whole sample (n = 250)				Control group (n = 125)				Treatment group (n = 125)				*Percentage of variations in mean costs between treatment and control groups*^b^	Unadjusted regressions comparing costs of treatment and control groups		
	Median	Mean	Mean for positive costs	n with costs>0^a^	Median	Mean	Mean for positive costs	n with costs>0^a^	Median	Mean	Mean for positive costs	n with costs>0^a^		*Relative risk* *(RR)*^c^	*95% CI*	*P value*
Total	1,164	3,754	3,785	248	1,178	3,838	3,869	124	1,083	3,670	3,700	124	-4	0.96	0.62–1.47	0.83
Ambulatory	330	718	742	242	377	754	779	121	290	682	704	121	-10	0.90	0.58–1.42	0.65
Somatic inpatient	337	2,424	4,040	150	434	2,390	3,879	77	176	2,458	4,209	73	3	1.03	0.59–1.80	0.90
Rehabilitation	0	204	2,555	20	0	157	2,178	9	0	252	2,863	11	61	1.24^d^	0.50–3.11	0.64
Psychiatric	0	408	2,914	35	0	537	3,198	21	0	279	2,490	14	-48	0.62^d^	0.30–1.29	0.20
ED	164	378	457	207	232	419	508	103	153	338	406	104	-19	0.81	0.54–1.21	0.29

**Notes**: All costs are monthly costs and expressed in CHF.

^a^ Number of observations with costs >0 are reported.

^b^ Variations in mean costs between treatment and control groups expressed in percentage of variations.

^c^ For each cost outcome, the relative risk (RR) from the unadjusted gamma-log GLMs regressions is reported.

^d^ Because of data distribution (few individuals with costs greater than 0; see columns reporting the number of observations with costs >0), logit models were run to identify differences between groups. This assesses differences in the probability of having costs greater than 0 rather than differences in averaged costs.

Patients in the treatment group had lower ED (-19%), ambulatory (-10%), psychiatric (-48%), and total (-4%) costs than did the control group, although the differences were not statistically significant. Somatic inpatient costs for patients in the treatment group were 3% higher than those for the control group, but the difference, again, was not statistically significant. Participants who died during the study had on average significantly higher total costs than did patients who were still alive at the end of the follow-up period (see Table A in S1 Text for details).

**Table 3** shows the costs from the hospital perspective, as the exponentiated coefficients of gamma-log GLMs for the 3 main cost outcomes: total, ambulatory, and ED. Regressions on total hospital costs exhibited an RR that was lower than that for the treatment group, indicating that CM was associated with decreased costs. However, this RR was not statistically significant (RR = 0.86, *p* = 0.45). All else being equal, dying during the trial was strongly associated with higher costs (RR = 3.69, *p*<0.001). A higher age, Swiss citizenship, having at least one social difficulty, and having at least one mental disorder were positively and significantly associated with higher total costs (*p*<0.001, *p* = 0.01, *p* = 0.07, *p* = 0.06, respectively). The other variables, such as gender, having a somatic condition, or having a risky behavior with respect to drug and alcohol use did not have a significant impact on total hospital costs.

**Table 3:** Results of gamma-log GLMs for cost outcomes evaluated from the hospital perspective: Total, ambulatory, and ED costs.

	Total monthly hospital costs				Monthly ambulatory costs				Monthly ED costs			
	RR	Std. Err.	95% CI	P value	RR	Std. Err.	95% CI	P value	RR	Std. Err.	95% CI	P value
Group (ref = CM group)	0.86	0.17	0.59–1.27	0.45	0.94	0.19	0.63–1.39	0.75	0.84	0.12	0.63–1.13	0.26
Dying during follow-up (ref. = alive at the end of follow-up)	3.69***	1.30	1.85–7.35	<0.001	1.08	0.35	0.57–2.06	0.81	2.33**	0.66	1.34–4.07	<0.003
Age	1.02***	0.00	1.01–1.04	<0.001	1.01	0.00	1.00–1.02	0.23	1.02***	0.00	1.01–1.03	<0.001
Swiss citizenship (ref. = no Swiss citizenship)	1.73*	0.37	1.13–2.64	0.01	1.31	0.26	0.89–1.93	0.18	1.81***	0.30	1.31–2.51	<0.001
Male gender	0.83	0.16	0.57–1.21	0.33	0.91	0.15	0.65–1.27	0.58	0.73*	0.11	0.55–0.99	0.04
Social difficulty	1.44	0.29	0.97–2.15	0.07	1.58*	0.30	1.09–2.29	0.02	1.69***	0.27	1.24–2.31	<0.001
Somatic condition	0.85	0.20	0.54–1.36	0.51	0.88	0.22	0.54–1.46	0.63	1.2	0.20	0.85–1.69	0.3
Mental health problem	1.45	0.28	0.99–2.11	0.06	1.47*	0.27	1.03–2.11	0.03	1.12	0.16	0.83–1.51	0.45
Risky behavior	0.72	0.16	0.46–1.12	0.14	0.66	0.15	0.43–1.02	0.06	0.88	0.15	0.62–1.24	0.46
Not having primary care physician	1.09	0.31	0.61–1.93	0.78	1.23	0.38	0.67–2.28	0.51	1.04	0.25	0.65–1.68	0.86
	AIC = 18		BIC = −819.049		AIC = 15.12		BIC = −938.90		AIC = 13.51		BIC = −1069.22	
Observations	250				250				250			

**Notes:** The 3 main outcomes of hospital costs were analyzed. RR: relative risk (exponentiated coefficients); Std. Err.: standard error; CI: confidence interval;

AIC: Akaike information criterion; BIC: Bayesian information criterion.

* *p* < 0.05.

** *p* < 0.01.

*** *p* < 0.001

Multivariate regressions of the 2 other types of hospital costs, namely, ambulatory and ED, also showed a statistically significant impact of social difficulty on costs. CM had no significant effect on these costs. The other variables affected ambulatory and ED costs differently. Dying during the follow-up period, age, and Swiss citizenship were significant predictors of higher ED costs, whereas being male was associated with lower ED costs. Mental disorders were associated with higher ambulatory costs, but having a risky behavior was associated with lower ambulatory costs (*p* = 0.06).

From a broader perspective of analyzing insurance costs related to services used within and used outside the hospital, the descriptive statistics presented in Table 4 show that, on average, monthly total insurance costs of frequent ED users amounted to CHF 2,150. Compared with those in the control group, patients in the treatment group had 24% higher intramural costs, a nonsignificant difference (*p* = 0.35).

**Table 4:** Cost data from insurance perspective: Total, intramural, and extramural costs for control and treatment groups.

Insurance monthly costs	Total (n = 140)				Control group (n = 65)				Treatment group (n = 75)				*Percentage of variations in mean costs between treatment and control groups*^*b*^	Unadjusted regressions comparing costs of treatment and control groups		
	Median	Mean	Mean for positive costs	n with costs>0^a^	Median	Mean	Mean for positive costs	n with costs>0^a^	Median	Mean	Mean for positive costs	n with costs>0^a^		*Relative risk* *(RR)*^*c*^	*95% CI*	*P value*
Total insurance costs	1,297	2,147	2,147	140	1,009	2,046	2,046	65	1,628	2,235	2,235	75	9	1.09	0.75–1.60	0.64
Intramural costs	485	1,249	1,267	138	387	1,104	1,122	64	730	1,374	1,392	74	24	1.24	0.78–1.98	0.35
Extramural costs	527	898	898	140	519	942	942	65	546	861	861	75	-9	0.91	0.60–1.40	0.62

**Notes**: Costs are monthly costs and expressed in CHF. Insurance costs are associated with health services used within the hospital that housed the intervention (intramural costs) and with those used outside the hospital (extramural costs).

^a^ The number of observations with costs >0 are reported.

^b^ Variations in mean costs between treatment and control groups expressed in percentage of variations.

^c^ For each cost outcome, the relative risk (RR) from the unadjusted gamma-log GLM regressions is reported for the restricted sample of 140 individuals.

To check for potential sample selection biases regarding the availability of insurance cost data, we compared the sample characteristics between treatment and control groups of the restricted sample (n = 140). They were similar regarding their demographic, clinical, and social characteristics (see Table A in S2 Text), as well as their total hospital costs (Table B in S2 Text).

Multivariate regressions of insurance costs outcomes (Table 5) showed that, among frequent users, age (RR = 1.02; 95% CI = 1.01–1.04) and social difficulties (RR = 1.88; 95% CI = 1.3–2.7) were positively and significantly associated with total, intramural, and extramural insurance costs. Risky behaviors regarding alcohol or drugs did not impact insurance costs. There was a positive and statistically significant association between mental health problems (RR = 1.78; 95% CI = 1.16–2.75) and intramural insurance costs.

**Table 5:** Results of the gamma-log GLMs for total, intramural, and extramural costs evaluated from the insurance perspective.

	Total Insurance costs				Intramural costs				Extramural costs			
	RR	Std. Err.	95% CI	P value	RR	Std. Err.	95% CI	P value	RR	Std. Err.	95% CI	P value
Group (CM group = reference)	1.10	0.19	0.78–1.55	0.58	1.22	0.26	0.80–1.86	0.36	0.92	0.17	0.64–1.33	0.67
Age	1.02***	0.00	1.01–1.04	<0.001	1.02***	0.00	1.01–1.04	<0.001	1.03***	0.00	1.01–1.04	<0.001
Swiss citizenship (ref = no Swiss citizenship)	1.54*	0.30	1.05–2.27	0.03	1.60*	0.38	1.00–2.55	0.05	1.44	0.33	0.92–2.26	0.11
Male gender	0.87	0.14	0.63–1.19	0.39	0.96	0.19	0.65–1.42	0.83	0.8	0.15	0.54–1.17	0.24
Social difficulty	1.88***	0.34	1.31–2.70	<0.001	1.66*	0.37	1.06–2.59	0.03	2.24***	0.45	1.51–3.33	<0.001
Somatic condition	1.15	0.26	0.73–1.81	0.55	1.34	0.39	0.75–2.41	0.32	1.01	0.23	0.65–1.59	0.95
Mental health problem	1.51*	0.28	1.05–2.16	0.03	1.78**	0.39	1.16–2.75	0.01	1.22	0.22	0.84–1.75	0.29
Risky behavior	0.93	0.19	0.62–1.39	0.72	0.77	0.19	0.47–1.28	0.31	1.17	0.23	0.79–1.72	0.44
Not having primary care physician	1.06	0.29	0.62–1.82	0.82	1.28	0.47	0.62–2.63	0.51	0.84	0.21	0.51–1.39	0.49
	AIC = 17.19		BIC = -496.20		AIC = 16.04		BIC = -408.13		AIC = 15.46		BIC = -400.20	
Observations	140				140				140			

**Notes**: Intramural costs are those associated with health services used within the hospital that housed the intervention, whereas extramural costs are those associated with health services outside the hospital. RR: relative risk (exponentiated coefficients); Std. Err: standard error; CI: confidence interval; AIC: Akaike information criterion; BIC: Bayesian information criterion.

* *p* < 0.05.

** *p* < 0.01.

*** *p* < 0.001.

### 4. Discussion

This study assessed the impact on costs of a CM intervention targeting frequent users of the ED of Lausanne University Hospital, defined as at least 5 visits in the 12 months prior to enrollment. Despite differences in costs between patients in the intervention and the control groups, our results do not show any significant reduction in costs associated with the intervention, either for the hospital that housed the intervention or for the insurance companies covering health care costs. Despite these overall negative results, our approach adds to the literature on CM interventions by investigating their impact on several types of costs evaluated from both hospital and insurance perspectives.

From the hospital perspective, the cost components associated with ED visits, ambulatory care, inpatient stays, and psychiatric care did not differ between patients receiving CM and patients receiving SC. In a similar clinical setting and from the hospital perspective as well, Shumway et al. showed that a CM intervention led to a reduction in the number of ED visits and in ED costs [13]. However, the authors did not find any reduction in the costs of other health services provided by the hospital such as inpatient care, ambulatory care, and psychiatric services for CM patients compared with those for SC patients.

From the insurance perspective, the evaluation of health care resources used by frequent ED users underlines several important points. With monthly insurance costs of CHF 2,150, health expenditures of frequent users are almost 5 times higher than the average monthly insurance costs per insured resident living in the same region. In 2013, the average monthly health insurance costs in the canton of Vaud were CHF 483 per inhabitant [38]. The magnitude of this difference demonstrates that this particular group may have an important impact on the health system as a whole. This result is consistent with previous studies showing that a majority of frequent ED users had significantly higher levels of health care use, including primary care [39–41]. In our sample, about 86% of the patients reported having a primary care physician. Hence, it is probably not the lack of access to primary care that leads these patients to seek emergency care. In the United States, frequent ED users were more likely than infrequent users to have a primary care physician [42]. Thus, this population does not necessarily use ED as a “substitute” for primary care, but likely uses it because of more pronounced needs for health care overall [40].

Previous articles have underlined that many frequent ED users have broad and complex medical and nonmedical needs. This vulnerable population subgroup often accumulates somatic and psychiatric disorders [3, 43], as well as precarious social situations [44]. These factors are clearly highlighted in our study, in which 51% of individuals had mental health problems, 70% somatic conditions, and 73% social difficulties.

As discussed in the literature that seeks to differentiate between the respective influence of age, proximity of death, and morbidity on health care costs of aging populations [45–48], our analysis showed that the use of health care resources increases in the period close to death. From the hospital perspective, total costs associated with frequent users who died during the study were on average 4 times higher than those associated with patients who were still alive. In particular, inpatient costs were 5 times higher and ED costs more than 3 times higher. Before a patient dies, morbidity often increases, leading to more treatments and thus to an increase in health care use and costs. Multivariate regressions, when demographic variables were controlled for, also underlined that dying has an impact on health care costs. More generally, our multivariate regressions add to the knowledge about frequent ED users by identifying which of the frequent users’ characteristics drive their health care costs. Thus, beyond proximity of death, age, and citizenship, mental disorders and social difficulties, including social isolation, housing instability, and financial insecurity, play a crucial role by driving both higher costs for the hospital and higher health expenditures for the insurer. Overall, these results provide information on the aspects that call for special attention in the implementation of CM interventions and the follow-up of these patients. Thus, as was the case for the CM intervention implemented at Lausanne University Hospital, improving the social situation of frequent ED users may be beneficial for the individuals themselves, for the hospital ED, and for the health care system overall.

Our study highlights that, among the insurance costs in the studied population, about 40% occurred outside the hospital that housed the intervention, underlining the importance of considering a perspective that is broader than that of the hospital. However, frequent users may turn to the hospital for specific types of care such as those related to mental disorders, which were found to affect intramural but not extramural costs. Indeed, 60% of health care costs on average were induced by health care services, including a mix of inpatient and ambulatory care for somatic and psychiatric care provided at Lausanne University Hospital, indicating a certain preference for the hospital. The distance between the health care providers and the patient’s residence is known to play a role in the choice of health care provider. Previous studies have shown that most of the frequent users lived within 8 kilometers of the ED [43, 44].

Our analysis addressed several shortcomings of the literature by analyzing health care resources—expressed as global insurance costs—used outside the hospital that housed the intervention. However, identifying the nature of every service—primary care and other hospital services—would have been relevant in order to check whether and to what extent frequent users use emergency care in other hospitals. [41], who did not find that CM intervention was successful in decreasing ED visits by frequent users (>10 ED visits in 21 months), tracked ED use at the university hospital that housed the intervention, as well as at other community hospitals in the city. Their figures showed that ED visits in other community hospitals were also substantial and thus worth taking into account.

The study has several limitations. First, the costs of the intervention itself were impossible to assess. Indeed, some of the physicians working as members of the CM team also delivered care to the SC group. Thus, it was not possible to evaluate the time specifically allocated to each of the 2 groups. From the hospital perspective, the management of these 250 frequent users over the study period cost approximately CHF 4.3 million overall for patients in the CM group versus CHF 5.0 million for SC patients, that is, almost CHF 700,000 less. It would have been interesting to learn the extent to which the investment in the CM program may have been compensated for by the reduction in treatment costs. However, our results suggest that the intervention may have interesting economic mechanisms and affect hospital productivity. Treatment is associated with a 14% economic reduction in total costs from the hospital perspective on the one hand (RR = 0.86; see Table 3) and with an economic increase in intramural insurance costs on the other. CM is associated with an increase in hospital revenues paid by the insurance companies and thus, the CM intervention may lead to an increase in hospital productivity. CM intervention may also reduce ED visit costs, allowing the identification of patients with unmet needs and their appropriate outpatient care. Further investigations are required regarding these mechanisms and impacts because our results are not statistically significant. A second limitation of the study concerns its power. The sample size may not be sufficient to perform cost analyses and find statistical differences between groups. The protocol established that 250 patients (125 in each group) was the sample size required to detect a between-group average reduction in the primary outcome of the study (a reduction of 2 visits per year to the ED) [27]. However, no calculations were made to determine the required sample size to detect a between-group average reduction in costs. Moreover, the sample studied in the cost analysis from the insurance perspective was reduced to a subsample (140/250), limiting the statistical power of the analysis.

Like most studies investigating the impact of a CM intervention, the time frame of the analysis was relatively short and did not exceed several months. Given the multiple and complex needs of these patients, time may be needed for such an intervention to take effect. A longer follow-up period could have elicited long-term and potentially significant effects in reducing health care use and expenditures. Frequent users may require long and intensive support to recover from problems such as mental health disorders, addiction, and social difficulties [49]. In addition, analyzing the trends and changes in costs over a longer period could be interesting. In a first step, one might expect that the intervention, by allowing identification of needs, would lead to an increase in health care use and then its costs. In a second step, the services offered by the intervention, by improving health status and social conditions, might induce a reduction in health care service use and its associated costs.

## 5. Conclusion

In this study, we used data from an RCT to compare a CM intervention with SC among frequent ED users of an urban public hospital in CH. The results show that frequent users of health care services inside the hospital that housed the intervention are also frequent users of health care services outside the hospital. This specific group has above-average health care costs, mainly driven by complex clinical (somatic and psychic) conditions and social determinants. The role of the CM team is to guide patients through the care process and provide social support. Patient-centered care is part of a continuous integration of medical and social dimensions, and the location of intervention is not limited to the hospital but often extends into the community. Further investigations with a larger sample, an increased power of analysis, and over a longer study period are required to validate the extent to which a CM intervention may improve the management of frequent users at the ED and reduce the economic burden that these patients place on hospital budgets, as well as on the health care system overall.

## Supporting information

S1 Text Information on patients who died during follow-up. (DOC)

S2 Text Information of the restricted sample (N = 140). (DOC)

S3 Text Consort checklist. (DOC)

S4 Text Publications original protocol. (PDF)

S1 Fig Consort flow chart. (TIFF)
