# Supplementary material for: Health care costs of case management for frequent users of the emergency department: Hospital and insurance perspectives
Source: PLoS One. 2018 Sep 24;13(9):e0199691. doi: 10.1371/journal.pone.0199691 (PMC6152853; doi:10.1371/journal.pone.0199691)
Supplement: S1 Text — (DOC) [file pone.0199691.s001.doc]

**S1 Text. Information on patients who died during follow-up**

Table A shows that participants who died during the study had on average significantly higher total costs than did patients who were still alive at the end of the follow-up period (RR=4.23, 95% CI=2.70-6.62). In particular, this was the case for the somatic inpatient (RR=5.60, 95% CI =3.20-9.81) and ED costs (RR=3.71, 95% CI=2.09-6.58), which were higher for people who died than for those who did not. By contrast, there were no significant differences between the group of patients alive and those who died in terms of ambulatory and psychiatric costs.

Table A**: Cost data from the hospital perspective for the 20 patients who died during follow-up**

| **Hospital perspective monthly costs** | **Median** | **Mean** | ***Relative risk comparing costs between dead and alive participants*†** | **95% CI** | ***P* values** |
| --- | --- | --- | --- | --- | --- |
| Total | 8,055 | 12,614 | 4.23 | 2.70-6.62 | <0.001 |
| Ambulatory care | 222 | 760 | 1.06 | 0.6-1.89- | 0.834 |
| Somatic inpatient | 6,534 | 9,922 | 5.60 | 3.20-9.81 | <0.001 |
| Rehabilitation care | 0 | 1,639 | 8.99‡ | 3.07-26.36 | <0.001 |
| Psychiatric care | 0 | 293 | 0.30‡ | 0.04-2.34 | 0.25 |
| Emergency department | 916 | 1,153 | 3.71 | 2.09-6.58 | <0.001 |

**Notes**: All costs are expressed in Swiss francs.

†For each outcome of costs, relative risks (RRs) of being dead or still alive of the unadjusted regressions are reported.

‡Because of data distribution (few individuals with a positive outcome), logit models were run to identify differences between groups. This assesses differences in the probability of having positive costs rather than differences in averaged costs.
