# Supplementary material for: Health care costs of case management for frequent users of the emergency department: Hospital and insurance perspectives
Source: PLoS One. 2018 Sep 24;13(9):e0199691. doi: 10.1371/journal.pone.0199691 (PMC6152853; doi:10.1371/journal.pone.0199691)
Supplement: S2 Text — (DOC) [file pone.0199691.s002.doc]

**S2 Text. Information of the restricted sample (N=140)**

Tables A and B provide descriptive statistics for the treatment and control groups of the restricted sample. They were similar regarding their demographic, clinical, and social characteristics, as well as their total hospital costs. No differences were found between groups in terms of determinants of health or demographic characteristics.

**Table A: Socio-demographic and medical characteristics of the subsample studied (N=140)**

|  | **Total (n=140)** | **Control group** | **Intervention group (n =75)** | ***P* values of test *** |
| --- | --- | --- | --- | --- |
|  |  | **(n =65)** |  |  |
| Male | 57.14 (80) | 61.54 (40) | 53.33 (40) | 0.33 |
| Age, mean (SD) | 45.42 (17.92) | 44.99 (19.62) | 45.81 (16.43) | 0.77 |
| Citizenship (being Swiss) | 48.56 (68) | 50.76923 (33) | 46.66667 (35) | 0.63 |
| Social determinants (at least one diagnosis from the following: complex family situation, social isolation, financial hardship, inadequate housing, lack of employment or other activities, problems with immigration status, limited French proficiency) | 72.85 (102) | 69.23 (45) | 76 (57) | 0.37 |
| Somatic determinants (at least one diagnosis from the following: chronic and/or acute severe illness, comorbidity, polypharmacy, treatment nonadherence, physical handicap) | 69.28 (97) | 67.69 (44) | 70.66 (53) | 0.70 |
| Mental determinants (at least one diagnosis from the following: depression, anxiety, personality and psychotic disorders) | 47.14 (66) | 49.23 (32) | 45.33 (34) | 0.65 |
| At-risk behaviors (any of the following diagnoses: alcohol abuse problem, illicit drug use, tobacco use, game addiction) | 30 (42) | 30.7 (20) | 29.33 (22) | 0.85 |
| Not having a primary care physician | 12.80 (18) | 15.38 (10) | 10.66 (8) | 0.40 |

**Notes**: All data are reported as number (%), except where otherwise indicated.

^*^ *P* value of chi-square tests are reported except for age, where a *t*-test was computed.

**Table B: Costs evaluated from the hospital perspective for the entire restricted sample (n=140) and both treatment and control group subsamples**

| **Hospital**  **perspective**  **monthly costs** | **Total (N=140)** | | | | **Control group (N=65)** | | | | **Treatment group (N=75)** | | | | ***Percentage of variation in mean costs between treatment and control groups^b^*** | **Unadjusted regression comparing costs of treatment and control groups** | | |
| --- | --- | --- | --- | --- | --- | --- | --- | --- | --- | --- | --- | --- | --- | --- | --- | --- |
|  | **Median** | **Mean** | **Mean for positive costs** | **n with costs>0^a^** | **Median** | **Mean** | **Mean for positive costs** | **n with costs>0^a^** | **Median** | **Mean** | **Mean for positive costs** | **n with costs>0^a^** |  | ***Relative risk (RR)****^c^* | ***95% CI*** | ***P value*** |
| Total | 1,089 | 2,560 | 2,597 | 138 | 982 | 2,540 | 2,580 | 64 | 1,184 | 2,576 | 2,611 | 74 | 1 | 1.01 | 0.62-1.63 | 0.95 |
| Ambulatory | 349 | 734 | 761 | 135 | 371 | 660 | 681 | 63 | 344 | 798 | 832 | 72 | 21 | 1.21 | 0.64-2.28 | 0.55 |
| Somatic inpatient | 233 | 1,412 | 2,381 | 83 | 322 | 1,391 | 2,379 | 38 | 168 | 1,430 | 2,383 | 45 | 3 | 1.06^d^ | 0.54-2.09 | 0.85 |
| Rehabilitation | 0 | 52 | 1,225 | 6 | 0 | 95 | 2,062 | 3 | 0 | 16 | 388 | 3 | -83 | 0.86^d^ | 0.16-4.42 | 0.85 |
| Psychiatric | 0 | 361 | 2,199 | 23 | 0 | 395 | 2,332 | 11 | 0 | 332 | 2,078 | 12 | -16 | 0.93^d^ | 0.38-2.28 | 0.88 |
| ED | 164 | 320 | 386 | 116 | 164 | 328 | 394 | 54 | 163 | 313 | 378 | 62 | -5 | 0.95 | 0.59-1.54 | 0.84 |

**Notes**: All costs are expressed in Swiss francs.

^a^ Number of observations with costs >0 are reported.

^b^ Variations in mean costs between treatment and control groups expressed in percentage of variations.

^c^ For each cost outcome, the relative risk (RR) from the unadjusted gamma-log GLM regressions is reported.

^d^ Because of data distribution (few individuals with costs greater than 0; see columns reporting the number of observations with costs >0), logit models were run to identify differences between groups. This assesses differences in the probability of having costs greater than 0 rather than differences in averaged costs.
