# Supplementary material for: Health care costs of case management for frequent users of the emergency department: Hospital and insurance perspectives
Source: PLoS One. 2018 Sep 24;13(9):e0199691. doi: 10.1371/journal.pone.0199691 (PMC6152853; doi:10.1371/journal.pone.0199691)
Supplement: S4 Text — (PDF) [file pone.0199691.s004.pdf]

Bodenmann et al. BMC Health Services Research 2014, 14:264
http://www.biomedcentral.com/1472-6963/14/264

BMC Health Services Research

STUDY PROTOCOL | Open Access

Case management for frequent users of the emergency department: study protocol of a randomised controlled trial

Patrick Bodenmann[1*], Venetia-Sofia Velonaki[2], Ornella Ruggeri[2], Olivier Hugli[3], Bernard Burnand[4], Jean-Blaise Wasserfallen[5], Karine Moschetti[5,6], Katia Iglesias[4], Stéphanie Baggio[7] and Jean-Bernard Daeppen[8]

Abstract

Background: We devised a randomised controlled trial to evaluate the effectiveness and efficiency of an intervention based on case management care for frequent emergency department users. The aim of the intervention is to reduce such patients' emergency department use, to improve their quality of life, and to reduce costs consequent on frequent use. The intervention consists of a combination of comprehensive case management care and standard emergency care. It uses a clinical case management model that is patient-identified, patient-directed, and developed to provide high intensity services. It provides a continuum of hospital- and community-based patient services, which include clinical assessment, outreach referral, and coordination and communication with other service providers.

Methods/Design: We aim to recruit, during the first year of the study, 250 patients who visit the emergency department of the University Hospital of Lausanne, Switzerland. Eligible patients will have visited the emergency department 5 or more times during the previous 12 months. Randomisation of the participants to the intervention or control groups will be computer generated and concealed. The statistician and each patient will be blinded to the patient's allocation. Participants in the intervention group (N = 125), additionally to standard emergency care, will receive case management from a team, 1 (ambulatory care) to 3 (hospitalization) times during their stay and after 1, 3, and 5 months, at their residence, in the hospital or in the ambulatory care setting. In between the consultations provided, the patients will have the opportunity to contact, at any moment, the case management team. Participants in the control group (N = 125) will receive standard emergency care only. Data will be collected at baseline and 2, 5.5, 9, and 12 months later, including: number of emergency department visits, quality of life (EuroQOL and WHOQOL), health services use, and relevant costs. Data on feelings of discrimination and patient's satisfaction will also be collected at the baseline and 12 months later.

Discussion: Our study will help to clarify knowledge gaps regarding the positive outcomes (emergency department visits, quality of life, efficiency, and cost-utility) of an intervention based on case management care.

Trial registration: ClinicalTrials.gov Identifier: NCT01934322.

Keywords: Randomised controlled trial, Case management, Emergency department, Frequent users, Quality of life

* Correspondence: Patrick.Bodenmann@hospvd.ch
[1]Department of Ambulatory Care and Community Medicine, University of Lausanne, Lausanne CH-1015, Switzerland
Full list of author information is available at the end of the article

BioMed Central

© 2014 Bodenmann et al.; licensee BioMed Central Ltd. This is an Open Access article distributed under the terms of the Creative Commons Attribution License (http://creativecommons.org/licenses/by/4.0), which permits unrestricted use, distribution, and reproduction in any medium, provided the original work is properly credited. The Creative Commons Public Domain Dedication waiver (http://creativecommons.org/publicdomain/zero/1.0/) applies to the data made available in this article, unless otherwise stated.

Background

Individuals attending emergency departments (ED) on a regular basis account for a disproportionally high number of all ED visits. LaCalle and Rabin [1] in their systematic review found that patients visiting an ED four or more times per year accounted for 4.5%–8% of all ED patients and 21%–28% of all ED visits. Emergency department frequent users (ED-FUs) attend the emergency department on multiple occasions; however, definitions and threshold numbers of visits vary across studies. According to Locker [2], the definition of five attendances or more per year corresponds to a non-random event and should be used to allow better comparisons between studies. ED-FUs present a higher rate of morbidity and mortality than less frequent ED users [3-7], are more at risk of drug and alcohol abuse [5,7-9], often present mental health issues [3,5,6,10], are more likely to visit for complications and exacerbations of chronic conditions [10,11], and are often homeless, uninsured, and from low socio-economic levels [3,12-14]. The majority of them believe that their complaints require immediate attention [1], and thus they constitute a significant burden on hospitals due to multiple visits and the number of problems they bring to the ED.

ED-FUs contribute significantly to ED overcrowding and extended waiting times, often due to inappropriate visits to the unit [15]. Overcrowding is detrimental to the quality of care in EDs. However, the severity of the reason for consultation at the ED is often controversial [1]. Indeed, several studies show that ED-FUs have non-emergency conditions [10,16-18] and could receive better care in settings other than an ED [19,20], which is not designed to provide continuous care to patients with non-emergency, chronic conditions. In addition, the numerous issues that ED-FUs have are not easily addressed by simply providing care alone. Appropriate and consistent medical and social services are needed for such vulnerable populations.

In response to these concerns, several institutions worldwide (e.g. in the United States, Canada, Sweden, the United Kingdom, the Netherlands, Spain, and Australia) [9,12,21-31] have introduced specific interventions for ED-FUs aimed at reducing the number of their visits, treating their medical co-morbidities, and/or addressing their social needs. Interventions vary, according to a recent systematic review of the literature by our research team that identified different types of interventions aimed at improving the management of adult ED-FUs [32] and at assessing interventions' effectiveness. Most of the studies describe interventions referring to and/or inspired by case management (CM) [9,12,25,29-31,33].

One of the most common interventions consists of CM multidisciplinary teams composed of nurses, psychologists, and possibly physicians [27,34-39]; this approach can help address complex situations and scenarios. Team members from different professional backgrounds, such as psychiatrists and health educators might complement the team, depending on the specific CM project. Coordination and organizational care tasks are often allocated to a case manager [37] who guides patients through the care process and provides social support. Care is generally considered as a continuous integration of medical and social dimensions. It is commonly patient-centered and holistic in nature, and takes patient empowerment [27,35,36] into account. Moreover, the locus of intervention is not limited to the hospital, and often extends into the community.

CM is a highly flexible and dynamic process and mainly depends on patient needs; the order of individual steps is often not constricted. In fact, its dynamic condition emphasizes that sometimes several steps take place simultaneously, or that the case manager has to return to a previous step. Based on the literature, this can be summarized in five steps [27,38-43]: identification, assessment/reassessment, planning, implementation, and evaluation/monitoring. The Behavioural Model for Vulnerable Populations [44] provides a theoretical framework for understanding how CM might improve the care of vulnerable patients; this theoretical framework suggests that the use of health services is a function of:

- predisposition of patients (demographics, health beliefs, social structure, and childhood characteristics);
- factors that enable or impede such use (personal, family, or community resources); and
- patient need for care (perceived and evaluated health).

CM guarantees that issues in each of these domains are addressed.

Interventions aimed at improving ED-FU management have had positive outcomes: some of the interventions evaluated have been effective in reducing emergency department use [9,12,21,24,26,29,31]. Cost-reduction analyses are also promising: Wassmer anticipated reductions in cost even when partially based on modeling estimates [31]; two other studies showed the effects of clinical case management on hospital services and its cost effectiveness [12,29]. Some interventions have had positive effects on social outcomes [12], such as a significant reduction in homelessness [25,29]. A positive effect on social outcomes is essential, as the link between social problems and health has been demonstrated by many authors [45]. Finally, clinical outcomes were assessed in three studies [12,25,29]; one of them demonstrating a positive effect in reducing alcohol and drug use [12].

In the literature, interventions aimed at improving the management of ED-FUs have demonstrated several positive outcomes, but there are still some knowledge gaps:

- There is only one randomised controlled trial (RCT) showing a significant reduction in ED use by FUs compared to patients receiving standard care [29].
- The threshold for number of visits varies across the three existing RCT [22,29,30]; only one is based on the definition of five or more attendances per year, corresponding to more than known random events [29].
- Cost reductions were demonstrated in three studies [12,29,31], but only one is an RCT [29], and the other two did not contain a control group.
- Patient baseline characteristics and health-care specificities shown in 11 studies included in a systematic review by Althaus and al. [32] were only relevant within the country in which each study was conducted (the US, Sweden, Canada, Australia, and the UK).

Because of the existence of the knowledge gaps mentioned above in a topic that is of the utmost importance for patients, clinicians, and policymakers, with this trial we would like to demonstrate that by establishing locally a model of care for these patients, we can decrease the use of the health-care system, improve these patients' quality of life, and reduce costs consequent on frequent use.

Aims and hypotheses

The primary aim of this study is to demonstrate that an intervention on ED-FUs by a multidisciplinary mobile team (based on CM care patterns) is a more appropriate way of reducing use of the ED - through a better orientation in the health-care system - and of improving quality of life than is standard emergency care delivered by nurses and physicians, and that it will reduce associated costs.

The study tests the hypotheses that CM intervention, as compared with standard emergency care,

- reduces ED attendance through a better orientation in the health-care system;
- improves quality of life;
- is a more efficient use of health-care resources (cost vs ED attendance); and
- leads to a favourable cost-utility ratio (cost vs Quality Adjusted Life Years (QALYs)).

Methods/design

Study design

This study is an RCT that compares comprehensive CM care associated to standard emergency care with standard emergency care alone among ED-FUs (Figure 1). The study includes a follow-up at 2, 5.5, 9, and 12 months after the first assessment.

Setting

The study will be conducted in the Lausanne University Hospital ED. This facility is an urban public hospital serving (with other non-university hospitals) 770,000 people. It provides medical, surgical, and mental health care via 50,000 annual ED visits, and is one of the five teaching university hospitals located in Switzerland.

Study population

Participants

Inclusion criteria Patients presenting at an ED between T0 and T1 (12 months), will be eligible to participate, provided they are at least 18 years of age, have made five or more visits to an ED in the previous 12 months, and are capable of communicating in any of the languages spoken by the team (i.e. French, German, Italian, English, or Spanish) or through a community interpreter.

Exclusion criteria Patients will not be enrolled if they cannot give informed consent or are ineligible to receive CM services (e.g. acutely confused, acutely psychotic, with dementia, or intoxicated), will not remain in Switzerland, or are not expected to survive for 18 months following enrollment. Additionally, those incarcerated, people expected to be imprisoned in the short term, and those with a family member who has already enrolled will be also excluded.

Flow diagram

The following flow diagram (Figure 2) shows the progression through the phases of the RCT of interventions based on a multidisciplinary mobile team case management pattern, parallel to standard emergency care for ED-FUs. The numbers given in the diagram are based on the results of a recent cross-sectional study conducted in the same setting at the Lausanne University Hospital ED (Bodenmann P. et al., in progress) and on the power analysis we conducted while designing the study.

Recruitment

Patient recruitment will last one year (T0–›T2).

Frequent user identification An automated 24-hour, seven-days-a-week detection system based on ED patient tracking software will identify all patients who will have attended the ED five times or more during the previous 12 months. A member of the CM team will approach each FU; the FU will receive written information, an oral explanation, and sufficient time to consider their opportunity to participate in the study. If the FU agrees, he or she will give his or her informal written consent. A psychologist will participate in the recruitment of the patients in order

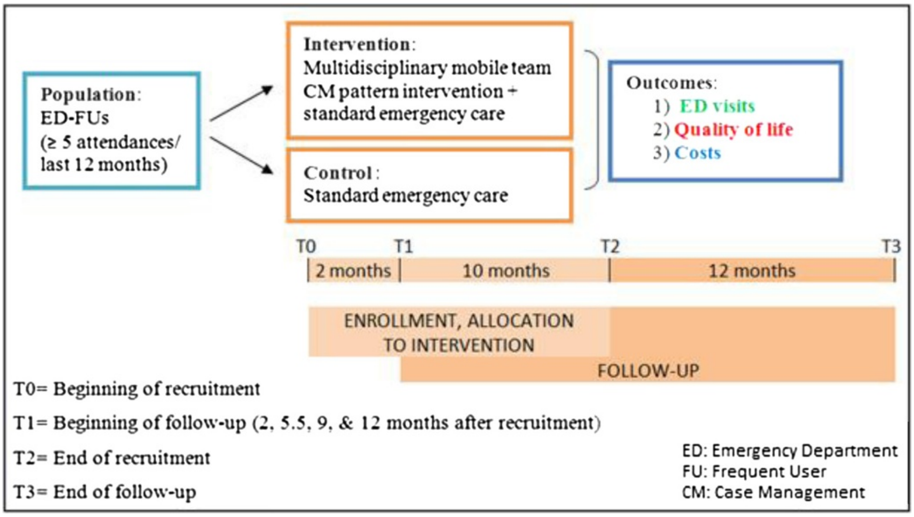

Figure 1 Study design: study design with inclusion and follow-up timetable.

to achieve better standardization of the process and to ensure increased motivation in the participants.

If a patient is no longer in the ED, a member of the CM team will make three attempts to contact that patient by telephone within 24–72 hours of their departure from the hospital, to briefly explain the study and try to organize a meeting. If the patients has a general practitioner he/she will be alerted by telephone, email, or mail by the team member in charge of their patient. The purpose of the contact is both

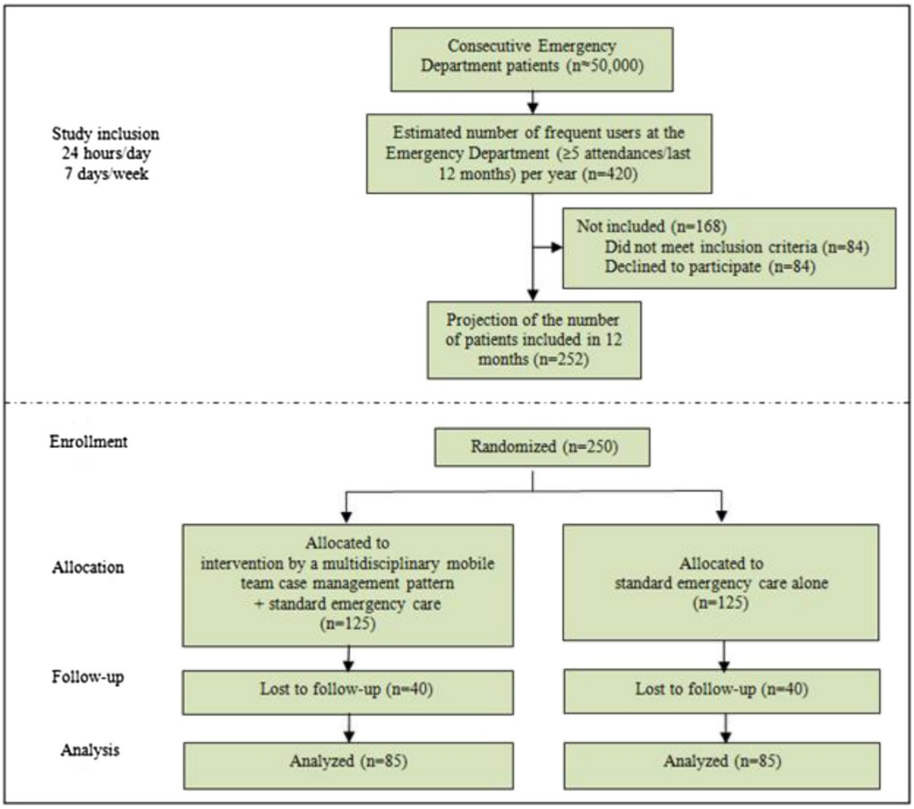

Figure 2 Flow diagram: flow diagram including estimates of numbers of patients to be included based on previously published [46,47] and unpublished (Bodenmann P. et al., in progress) studies of ED-FUs conducted by our research group and on the power analysis conducted during study design.

to inform the general practitioner and to get information from him/her.

Allocation to conditions

Sequence generation

The randomisation list associating questionnaire numbers to intervention or control groups will be generated by the statistician using block randomisation prior to the start of the study. Computer-based, randomly-generated, permuted blocks of random size will assure group size balance (www.randomization.com). Patients will then be allocated to either group A or group B. The research team will then decide if group A or B is to be the intervention group, therefore blinding the statistician to the true allocation. The randomisation list will be held by the research team. At night and during the weekends, the CM team will be informed of ED-FU consultations via email by the ED's staff. The CM team will contact each ED-FU the day after or on the following Monday, and if the patient agrees to participate in the study, the process of randomisation will take place in the research team office.

Allocation concealment mechanism

The statistician will hold the randomisation list and reveal each patient's allocation corresponding to the questionnaire number. The allocation will be reported to the CM team by phone once baseline characteristics have been collected by the CM team. The patient will then be informed about the procedures he or she should follow, without knowing whether he or she has been assigned to the intervention or to the control group.

Blinding

The *research nurse*, responsible for collecting outcomes, will not be blinded to the patient's allocation, as she had to have access to the database. The *CM team* will be blinded until randomisation. The *statistician* will be blinded to the true group until the analyses are complete. As the intervention is also provided by *ED staff* who will interact with the CM team for the intervention group patients, it is impossible to have them blinded. *Patients* will agree to take part in a study in which they will be managed by a coordinated team. *Blinding effectiveness* will be assessed by asking patients at the end of their follow-up period if they thought they were in the intervention or the control group. Since it delivers the intervention, the CM team cannot be blinded. The *data collection manager*, also responsible for quality control, will have access to all data and therefore cannot be blinded.

Interventions

The multidisciplinary mobile team CM pattern intervention

The mobile team consists of four nurses practitioners. A medical supervisor (general practitioner) stages the implementation of the project, monitors the team consolidation process, and is available for medical consultations for any difficult medical conditions in patients. He has the responsibility of verifying that the intervention offered is the preferred one.

Patients randomised to CM will receive an intervention designed to offer support for ED-FUs and the professionals who work with them inside the hospital, as well as for the community medical- and social providers who will maintain outside continuity of care:

- The CM team (four nurses) will meet the patient at the hospital or ambulatory EDs. First, they will complete an assessment of one and a half hours focussing on baseline characteristics, social determinants of health, mental and somatic diseases, risk behaviors, health-care use, and health literacy [48-50]. Second, the CM team will complete, with each patient, a questionnaire including instruments that assess quality of life (EuroQOL and WHOQOL) and feelings of discrimination.
- FUs will be seen initially by the team from one (ambulatory care) to three (hospitalization) times during their visits to the hospital and again one, three, and five months later at their home or in an ambulatory setting (Figure 3).
- In between the consultations provided, the FUs of the intervention group will have the opportunity to contact, at any moment, one of the members of the CM team in an "open-door policy perspective" with subsequent monitoring of the frequency and the content of every intervention required.
- Initial (Day 1) and follow-up interventions by the CM team (at one, three, and five months) will include counseling about social determinants of health, substance-use disorders (if relevant), and the use of medical care systems. Counseling will be based on motivational interviewing (empathy, collaboration, autonomy, and valorization), while avoiding confrontation. Each member of the CM team will have a checklist covering the proposals and advice that they have to give to every FU patient and outlining the material (flyers, addresses, etc.) that they have to provide.
- The primary goals of the interventions are to furnish specific assistance and to provide referrals for the patients:
  - *If the social determinants of an ED-FU are not adequate*, the team will —
    → provide assistance in obtaining income entitlements, health insurance coverage if eligible, stable housing (e.g. shelters for the homeless), schooling for children, prevention of potential

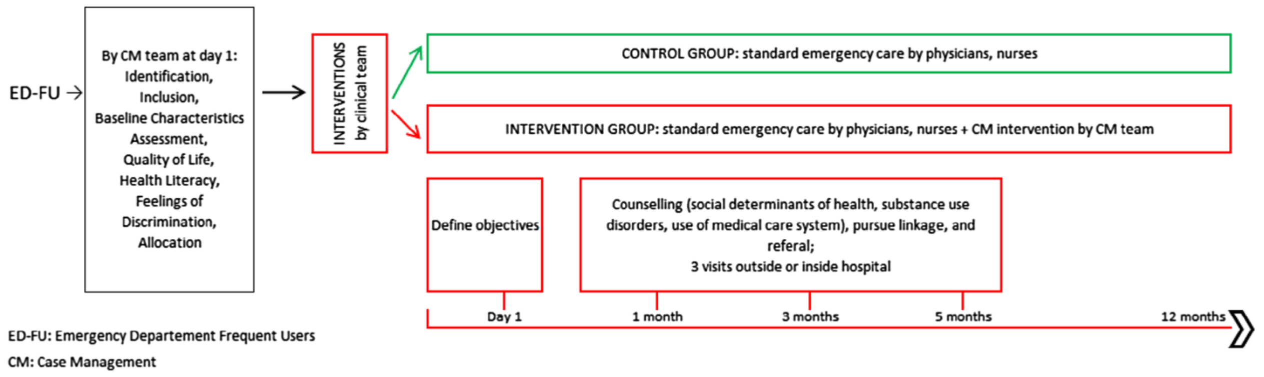

Figure 3 Timetable of the interventions: timetable for every ED-FU included in the study with interventions (at Day 1, 1 month, and 3 and 5 months for the intervention group).

violence (i.e. conjugal and/ or against the children) in the home.

- *If there are mental disturbances*, the team will — → refer patients to mental health departments inside the hospital, and if necessary, to a psychiatrist, psychologist, or general practitioner (GP) out in the community.
- *If the patient presents risk behaviors (alcohol consumption, smoking, or other drug use)*, the team will — → refer the patient to substance abuse services and provide links to community services in order to maintain continuity of care.
- *In cases of somatic problems* (and in which the patient either has no GP or has not consulted with their GP for a long time) the team will — → find a new GP or make contact with the previous provider, contingent on the patient's consent.

– Each member of the team will follow a maximum caseload of 20 patients as a case manager. We will take into account the CM team's capacity in order to ensure consistent recruitment over time: if the program reaches capacity, particularly when the intervention group of participants is enrolled, it may become necessary to stop recruitment until clinical capacity is again available.

– The linkage to medical and social services providers inside the hospital (with the participation of the CM team in network meeting crisis interventions organized by the professionals involved in each case) will continue outside the hospital with GPs and home visits by nurses and social services. The CM team will centralize documents and facilitate communication between all care providers, ensuring ongoing community outreach in order to maintain continuity of care.

This program uses an assertive clinical CM model that is patient-identified, patient-directed, and developed to provide high intensity services. It provides a continuum of hospital- and community-based patient services that includes clinical assessment, outreach referral, and coordination and communication with other service providers. Additional components are patient education in a motivational perspective, individual counseling, crisis intervention, medical assessment, and ongoing medical care.

Teamwork, case conferences, continuing education

The core team (nurse practitioners, and a general practitioner) is supported by several vulnerable population experts from various hospital departments - including gynaecology-obstetrics, paediatrics, psychiatry, and ethics - who act as contact persons for their respective departments and complement the team's interventions with their expertise on specific problems of gender, children who are minors, mental illness, and ethical concerns.

Members of the CM team will receive intensive training in motivational interviewing and cross-cultural competences, and will take specific classes in adequate referral to social assistance (e.g. income entitlements and stable housing), alcohol and drug use disorders, and home violence.

Because of the potential for difficult situations concerning many of these vulnerable patients, the members of the CM team will receive psychological support.

The control intervention

Patients randomised to the control group (standard care) will receive standard emergency care from physicians (resident or attending physician) and nurses, without the case manager being involved. Nevertheless, the mobile team will contact each patient in the control group, providing them with general information in the form of a flyer which will outline the existence of the mobile team, and provide relevant addresses and telephone numbers. A member of the team will then complete an assessment of one and a half hours focused on baseline characteristics,

social determinants of health, mental and somatic diseases, risk behaviors, health-care use, and health literacy [48-50]. Finally, the CM team will complete, with each patient, a questionnaire including instruments that assess quality of life (EuroQOL and WHOQOL) and feelings of discrimination.

The assessment effect, if any, will be present in the control group as in the intervention group.

Concerning standard care, after the first orientation by a nurse, when an intervention is provided by a resident he or she will be systematically supervised by a chief resident. Referrals to other specialists are routinely made by residents acting as liaisons to the appropriate hospitalization sector; there is no systematic presence or involvement of nurse practitioners.

Finally, patients randomised to standard care will be eligible to receive CM services at the end of the study. In any critical situation where a patient included in the study (in the intervention or the control group) needs hospitalization, that hospitalization will occur. Nevertheless, if the patient spontaneously contacts the clinical team by means of the phone number on the flyer, he or she will be able to benefit from an intervention by the CM team (as will the intervention group), after the end of the follow-up period for the patient.

Measurements - outcomes

Questionnaires for each patient will be filled in and data will be collected at the baseline and 2, 5.5, 9, and 12 months later to assess the outcomes of the intervention. The primary outcome measurement is the number of ED visits. Secondary outcome measurements are the standardized measurements of health status via EuroQol (EQ-5D) [51,52] and WHOQOL-BREF, cost analysis based on the use of health services, and an instrument on feelings of discrimination. (Figure 4).

Primary outcome: number of ED visits

The primary outcome will be the number of ED visits made by FUs. This information will mainly be available via the Lausanne hospital/ambulatory electronic records system and hospital/ambulatory administrative databases covering a period of 12 months after the initial emergency department visit.

"Care Notebooks" in hands of case and control participants will also be generated from the beginning of the study. Patients will be asked to report all visits (ED visits to any hospital and all outpatient visits) in their Care Notebook during the 12 months following their first visit (Figure 3). Patients will be contacted by telephone by the nurse researcher to answer questions about their use of the health-care system and to verify that they have completed their Care Notebooks appropriately. If this is not the case, the nurse researcher will help the patient find methods and strategies for improving their reporting. If necessary, incentives would be used to help FU patients complete Care Notebooks appropriately. The quality control will be repeated at 2-, 5.5-, 9-, and (finaly) 12-month

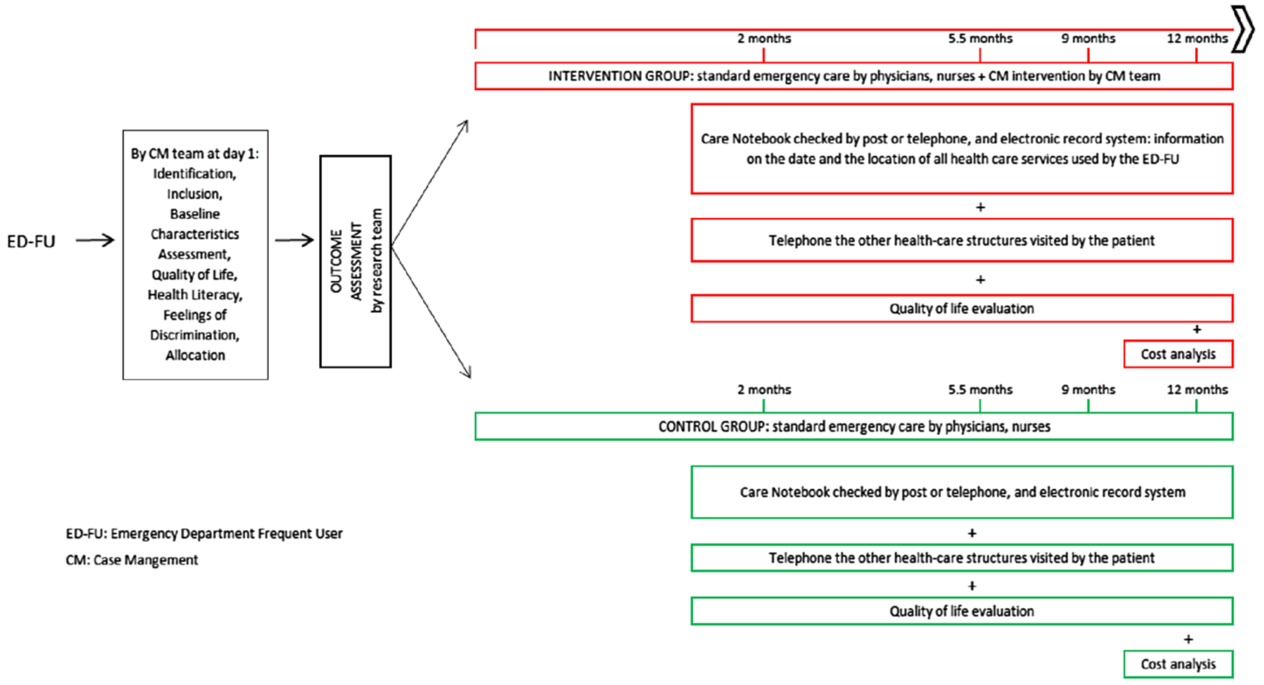

Figure 4 Timetable of the assessments: timetable for every ED-FU included in the study with outcome assessment (2, 5.5, 9, and 12 months for the intervention and the control group).

follow-ups. Confirmatory telephone calls to other hospitals, medical institutions, or private practices that the patient claims to have visited will be made by the nurse researcher, after obtaining the patient's written permission to do so.

Moreover, the validity of FU patients' answers could be assessed by matching their answers to our gold standard electronic records system of visits within our hospital and to the records of the participant's health insurance provider, after having obtained relevant informed consent.

Secondary outcome: cost analysis

The second outcome measurement focuses on the costs induced due to the health-care resources used by the FU patients. Their health-care consumption is related to services provided by our hospital but we cannot exclude that a FU also uses services provided by other hospitals/institutions in the community (services provided outside our hospital).

1) *Concerning services provided by our hospital*, different hospital administrative databases which record all inpatient and outpatient admissions will allow us to have access to details of all healthcare used by the FU and consequently the related costs. The latter will be composed of costs related to:
   a. outpatient resources induced by ED attendances,
   b. inpatient resources induced by ED attendances,
   c. non-ED related outpatient resources used within the hospital,
   d. the ED CM multidisciplinary team intervention.

Access to the accounting analytical systems of our hospital, as well as to the outpatient invoicing department, will allow the necessary information to be collected in order to calculate costs.

2) *Concerning services used outside our hospital*, information recorded in Care Notebooks will help to identify to what extent FUs seek and use services outside our hospital's boundaries, including whether patients use EDs of other hospitals, etc. The Care Notebook, by recording the date and the location of all visits the FU makes, will also help identify the types of services (health and/or social services) used by FUs.

The CM intervention may affect how the FUs use the health care system in general. The primary outcome of the project will allow us to identify whether the CM intervention is associated with a decrease in the number of visits to the ED of our hospital. However, it is also important to investigate to what extent the potential decrease in health-care resources used at the ED of our hospital is (or is not) associated with an increase in health-care resources used outside this specific ED. Consequently, information from the Care Notebook will help capture a substitution effect between health-care utilization at our hospital's ED and health-care utilization external to this specific ED. Based on average unit costs, costs associated with the health care consumption outlined in the Care Notebook will be simulated.

Additionally, having obtained the patient's informed consent we will contact their health insurer or the relevant health services provider in order to collect information on the frequency, type, and cost of health services that the participant has used during the study.

Secondary outcome: standardized measurement of health status via EuroQol (EQ-5D) and WHOQOL-BREF

Another secondary outcome will be the assessment of the health status of participants, as measured by the EQ-5D. This instrument is a non-disease-specific self-report of health-related quality of life. It is applicable to a wide range of health conditions and treatments, and provides a simple measurement of health that is used in clinical and economic analysis. Each respondent defines his/her own health status by combining one level (from a choice of three: "no problems", "some problems", "extreme problems") from each of five dimensions: mobility; self-care; usual activities; pain/discomfort; and anxiety/depression. For any state of health reported, an EQ-5D score reflecting a health utility weight will be derived.

Quality of life will also be assessed using the WHOQOL-BREF — an instrument developed by the World Health Organization. It is a 26-item Likert scale, which focuses on four aspects of quality of life: physical health, psychological health, social relationships, and environment. It also contains two items concerning the individual's overall satisfaction with life and general sense of personal well-being. Each response on this Likert scale is coded from 1 to 5.

To complement the assessment of the health status of participants, we will address patient satisfaction through a five-item questionnaire, validated locally.

Secondary outcome: feelings of discrimination

Additionally, an instrument assessing the feelings of discrimination will be filled in by each participant at the beginning of the study and 12 months later. The discrimination instrument was validated in a previous study conducted at the University Hospital of Geneva [53].

Sample size

The sample size has been calculated to detect a between-group average reduction of two visits per year to the ED (i.e. minus four visits for the intervention group versus minus two for the control, with an expected standard deviation of four in both groups), in

accordance with the results of a systematic review of the literature by Althaus et al. [32]. With a significance level of 0.05 and power of 0.9, each group should include at least 85 participants. "Given that an increased mortality rate of ED-FUs is described in the literature [54] and that, from previous observations from the CM team's clinical activities, 30% of our patients should be refugees or undocumented migrants, we expect an increased proportion of patients lost to follow-up. We therefore voluntarily overestimated the drop-out rate for the overall population to be 30% (80/250). The total required sample size has been rounded up to 250 patients (125 in each group).

Statistical methods

Groups will be compared from their initial allocation independently of eventual crossover (intention-to-treat analyses). The principle measurement of effect is an individual's average reduction in visits to the ED over 12 months compared to the number of visits observed in the control group. This will be calculated using linear regression with number of visits during 12 months' follow-up as an independent variable, and group allocation and yearly number of ED visits prior to intervention as dependent variables. Should group imbalance occur, secondary analysis will test the confounding effects by measuring the effect after adjusting for these confounding effects in the linear regression. Known determinants of frequent use are to be considered as potential confounders if, by chance due to the randomisation, we are to observe a relative difference of 20% between groups.

FUs are known to visit EDs on regular bases over a short period of time [55] (regression to the mean), so we also expect to see a decrease in the number of visits in the control group. Our analysis will measure the true effects of the intervention taking this phenomenon into consideration.

In terms of medico-economic analysis, benefits of the care management program will be evaluated by health gains expressed in Quality Adjusted Life Years (QALYs) over the 12-month period. A cost-utility analysis from the health-care provider perspective will be conducted by combining the use of two outcomes (i.e. costs and health status in terms of quality of life). A cost-utility ratio will then be calculated. A sensibility analysis will also be conducted in order to estimate the confidence interval for the cost-utility ratio. Uncertainty will be assessed by univariate and probabilistic sensitivity analysis (Monte Carlo simulation). All statistical analysis will be carried out with STATA 12.0, Statacorp, College Station, Texas, USA.

Ethical approval

The protocol, information letters, questionnaires, and the informed consent form of the study were approved by the Human Research Ethics Committee of the Canton of Vaud, Switzerland (no 32/12). There is no expected adverse event or side effect for participants.

Discussion

This study is coordinated with recent local research projects dedicated to assessing profiles and improving healthcare for ED-FUs, who are considered to be a highly vulnerable subgroup and a proxy for vulnerable populations in general.

At the Lausanne University Hospital ED, in 2008–2009, ED-FUs accounted for 4.4% of ED patients and 12.1% (n = 5,813) of all ED visits (n = 48,117) [46]. A retrospective chart review case–control study, conducted in this hospital between April 2008 and March 2009 by Bieler et al. [46], demonstrated that social (i.e. homelessness, institutionalization, unemployment, or dependence on government welfare) and specific medical vulnerability factors (i.e. ED primary diagnosis of substance abuse and the use of five or more clinical departments in the 12 previous months) increased the risk of ED use among 719 patients. A combination of social and medical factors was markedly associated with frequent ED use, as FUs were 10 times more likely to have three of them (of a total of eight factors; 95% CI = 5.1 to 19.6). This result is confirmed by Althaus et al. [56] in a retrospective chart review on hyperfrequent users (12 attendances or more during a year): they were 13 times more likely than non-FUs (65.5 vs 5.0%) to present three or more of the risk factors of vulnerability that Bieler et al. referred to [46] and 2.2 times more likely than FUs (62.5 vs 28.4%). Finally, unpublished, local, prospective, cross-sectional data (Bodenmann P. et al., in progress) obtained between November 2009 and June 2010 has demonstrated differences between 226 FUs and 173 infrequent users. FUs were more often younger with a mean age of 51 vs 56 in infrequent users, and the former had experienced five to 18 admissions in the previous 12 months. They cumulated vulnerabilities in terms of somatic problems, mental diseases, risk behavioral indicators, and unfavorable social determinants of health.

Taking care of a growing number of vulnerable patients requires specific interventions. A systematic review of the effectiveness of interventions targeting ED-FUs concluded that such interventions may reduce ED use and that CM, the most frequently described intervention, seemed to improve social and clinical outcomes and reduce ED costs in different studies [32]. Three studies [12,29,31], from which one RCT [29], concluded that CM could contribute to the reduction of ED use and of consequent costs, while two of these studies [12,31] found additionaly that CM could also lead to positive social outcomes. However, patterns of care that have succeeded elsewhere have to be tested in local or national

settings before being introduced into a new context of care among local patients. A mixed methodology using quantitative and medico-economic analysis is needed.

Responding to the knowledge gaps in the literature [57,58] and following our local studies through different observational designs, our hypothesis is that CM leads to reduced ED use by ED-FUs through a better orientation in the health-care system, improves their quality of life, and is more cost-effective than is standard emergency care alone provided by nurses and physicians serving ED-FUs. Positive findings would constitute a strong incentive to replicate these studies on a larger scale, in a multicenter study with more extensive follow-up procedures. Positive findings would also suggest that specific populations need specific care, and would have major implications for healthcare quality and costs. Finally, the total number of ED visits in Switzerland is around 1.3 million per year [59] and has been steadily growing. If our intervention results in a reduction in the number of ED visits, the impact at the national level could be significant.

Abbreviations

RCT: Randomised controlled trial; ED: Emergency department; FU: Frequent user; ED-FU: Emergency department frequent user; CM: Case management; GP: General practitioner.

Competing interests

The authors declare that they have no competing interests.

Authors' contributions

PB, VSV, and OR wrote the manuscript. All authors critically reviewed the manuscript for important intellectual content. The study design and research proposal were mainly developed by PB and JBD. OH, BB, JBW, KI, KM, and SB made substantial contributions to the conception and design of the study. PB, JBW, and KM performed the power analysis. The intervention was developed by PB, OH, and JBD. All authors agree to be accountable for all aspects of the work in ensuring that questions related to the accuracy or integrity of any part of the work are appropriately investigated and resolved. All authors have read and approved the final manuscript.

Acknowledgements

This study is funded by the Swiss National Science Foundation (no: 32003B_135762).
The authors gratefully acknowledge Stéphanie Stucki, Sophie Paroz, Séverine Alary, Jolanta Nobs, and the CM team (Dr Francis Vu, Corine Ansermet, Marina Canepa-Allen, Letitia LeNosher, and Joelle Schupbach) for having agreed to contribute to the collection of the data, and Mr Brooks (ELCS) for revising the English version of the manuscript.

Author details

[1]Department of Ambulatory Care and Community Medicine, University of Lausanne, Lausanne CH-1015, Switzerland. [2]Department of Community Medicine and Public Health, Lausanne University Hospital, Lausanne CH-1011, Switzerland. [3]Emergency Department, Lausanne University Hospital, Lausanne CH-1011, Switzerland. [4]Institute of Social and Preventive Medicine, Lausanne University Hospital, Lausanne CH-1011, Switzerland. [5]Health Technology Assessment Unit, University of Lausanne, Lausanne CH-1015, Switzerland. [6]Institute of Health Economics and Management, University of Lausanne, Lausanne CH-1015, Switzerland. [7]Life Course and Inequality Research Centre, Faculty of Social and Political Sciences, University of Lausanne, Lausanne CH-1015, Switzerland. [8]Alcohol Treatment Service, Lausanne University Hospital, Lausanne CH-1011, Switzerland.

Received: 28 May 2014 Accepted: 11 June 2014
Published: 17 June 2014

References

1. LaCalle E, Rabin E: **Frequent users of emergency departments: the myths, the data, and the policy implications.** *Ann Emerg Med* 2010, **56**(1):42–48.
2. Locker TE, Baston S, Mason SM, Nicholl J: **Defining frequent use of an urban emergency department.** *Emerg Med J* 2007, **24**(6):398–401.
3. Moore G, Gerdtz M, Manias E, Hepworth G, Dent A: **Socio-demographic and clinical characteristics of re-presentation to an Australian inner-city emergency department: implications for service delivery.** *BMC Public Health* 2007, **7**:320.
4. Dent AW, Phillips GA, Chenhall AJ, McGregor LR: **The heaviest repeat users of an inner city emergency department are not general practice patients.** *Emerg Med (Fremantle)* 2003, **15**(4):322–329.
5. Fuda KK, Immekus R: **Frequent users of Massachusetts emergency departments: a statewide analysis.** *Ann Emerg Med* 2006, **48**(1):9–16.
6. Hansagi H, Allebeck P, Edhag O, Magnusson G: **Frequency of emergency department attendances as a predictor of mortality: nine-year follow-up of a population-based cohort.** *J Public Health Med* 1990, **12**(1):39–44.
7. Huang JA, Tsai WC, Chen YC, Hu WH, Yang DY: **Factors associated with frequent use of emergency services in a medical center.** *J Formos Med Assoc* 2003, **102**(4):222–228.
8. Byrne M, Murphy AW, Plunkett PK, McGee HM, Murray A, Bury G: **Frequent attenders to an emergency department: a study of primary health care use, medical profile, and psychosocial characteristics.** *Ann Emerg Med* 2003, **41**(3):309–318.
9. Skinner J, Carter L, Haxton C: **Case management of patients who frequently present to a Scottish emergency department.** *Emerg Med J* 2009, **26**(2):103–105.
10. Malone RE: **Heavy users of emergency services: social construction of a policy problem.** *Soc Sci Med* 1995, **40**(4):469–477.
11. Lucas RH, Sanford SM: **An analysis of frequent users of emergency care at an urban university hospital.** *Ann Emerg Med* 1998, **32**(5):563–568.
12. Okin RL, Boccellari A, Azocar F, Shumway M, O'Brien K, Gelb A, Kohn M, Harding P, Wachsmuth C: **The effects of clinical case management on hospital service use among ED frequent users.** *Am J Emerg Med* 2000, **18**(5):603–608.
13. Sun BC, Burstin HR, Brennan TA: **Predictors and outcomes of frequent emergency department users.** *Acad Emerg Med* 2003, **10**(4):320–328.
14. Ullman R, Block JA, Stratmann WC: **An emergency room's patients: their characteristics and utilization of hospital services.** *Med Care* 1975, **13**(12):1011–1020.
15. Hsia RY, Tabas JA: **Emergency care: the increasing weight of increasing waits.** *Arch Intern Med* 2009, **169**(20):1836–1838.
16. Kne T, Young R, Spillane L: **Frequent ED users: patterns of use over time.** *Am J Emerg Med* 1998, **16**(7):648–652.
17. Oktay C, Cete Y, Eray O, Pekdemir M, Gunerli A: **Appropriateness of emergency department visits in a Turkish university hospital.** *Croat Med J* 2003, **44**(5):585–591.
18. Ovens HJ, Chan BT: **Heavy users of emergency services: a population-based review.** *CMAJ* 2001, **165**(8):1049–1050.
19. D'Aloiso L, Carlomagno F, Bisceglia M, Anaganti S, Ferretti E, Verrienti A, Arturi F, Scarpelli D, Russo D, Santoro M, Filetti S: **Clinical case seminar: in vivo and in vitro characterization of a novel germline RET mutation associated with low-penetrant nonaggressive familial medullary thyroid carcinoma.** *J Clin Endocrinol Metab* 2006, **91**(3):754–759.
20. Anonymous: **Reducing 'frequent flyers' in the emergency room: Medicaid members connect with primary care.** *Patient Education Management* 2008, **15**(6):67–68.
21. Andren KG, Rosenqvist U: **Heavy users of an emergency department: psycho-social and medical characteristics, other health care contacts and the effect of a hospital social worker intervention.** *Soc Sci Med* 1985, **21**(7):761–770.
22. Hansagi H, Olsson M, Hussain A, Ohlen G: **Is information sharing between the emergency department and primary care useful to the care of frequent emergency department users?** *Eur J Emerg Med* 2008, **15**(1):34–39.
23. Lee KH, Davenport L: **Can case management interventions reduce the number of emergency department visits by frequent users?** *Health Care Manag (Frederick)* 2006, **25**(2):155–159.
24. Olsson M, Hansagi H, Tomson Y, Sjoberg S: **Individualised care plans - a feasible way to improve care for frequent users of the emergency department?** *Socialmed Tidskr* 2004, **1**(2):122–129.

25. Phillips GA, Brophy DS, Weiland TJ, Chenhall AJ, Dent AW: **The effect of multidisciplinary case management on selected outcomes for frequent attenders at an emergency department.** *Med J Aust* 2006, **184**(12):602–606.
26. Pope D, Fernandes CM, Bouthillette F, Etherington J: **Frequent users of the emergency department: a program to improve care and reduce visits.** *CMAJ* 2000, **162**(7):1017–1020.
27. Powell SK, Tahan HA: *Case management. A practical guide for education and practice.* 3rd edition. Philadelphia: Wolters Kluwer | Lippincott Williams & Wilkins; 2010.
28. Sargent P, Pickard S, Shaeff R, Boaden R: **Patient and carer perceptions of case managmeent for long-term conditions.** *Health and Social Care in the Community* 2007, **15**(6):511–519.
29. Shumway M, Boccellari A, O'Brien K, Okin RL: **Cost-effectiveness of clinical case management for ED frequent users: results of a randomized trial.** *Am J Emerg Med* 2008, **26**(2):155–164.
30. Spillane LL, Lumb EW, Cobaugh DJ, Wilcox SR, Clark JS, Schneider SM: **Frequent users of the emergency department: can we intervene?** *Acad Emerg Med* 1997, **4**(6):574–580.
31. Wassmer RW, Winward L, Derlet R: *Does Counseling Reduce Frequent Emergency Department Use?.* 2008. http://www.csus.edu/indiv/w/wassmerr/ccp.pdf.
32. Althaus F, Paroz S, Hugli O, Ghali WA, Daeppen JB, Peytremann-Bridevaux I, Bodenmann P: **Effectiveness of interventions targeting frequent users of emergency departments: a systematic review.** *Ann Emerg Med* 2011, **58**(1):41–52. e42.
33. Lee A, Lau FL, Hazlett CB, Kam CW, Wong P, Wong TW, Chow S: **Factors associated with non-urgent utilization of accident and emergency services: a case–control study in Hong Kong.** *Soc Sci Med* 2000, **51**(7):1075–1085.
34. Brewer BB, Jackson L: **A case management model for the emergency department.** *J Emerg Nurs* 1997, **23**(6):618–621.
35. Bristow DP, Herrick CA: **Emergency department case management: the dyad team of nurse case manager and social worker improve discharge planning and patient and staff satisfaction while decreasing inappropriate admissions and costs: a literature review.** *Lippincotts Case Manag* 2002, **7**(6):243–251.
36. Case Management Society of America: *Case management model act.* Arkansas; 2009.
37. Schneidermann M, Ferandez A: **Case management / multidisciplinary care models.** In *Medical management of vulnerable and underserved patients.* Edited by King T, Wheeler M. New York: McGraw-Hill; 2007:151–158.
38. Walsh KT, Zander K: *Emergency department case management.* Marblehead: HCPro, Inc.; 2007.
39. Wetta-Hall R, Berry M, Ablah E, Gillispie JM, Stepp-Cornelius LK: **Community case management: a strategy to improve access to medical care in uninsured populations.** *Care Manag J* 2004, **5**(2):87–93.
40. Berger CS: **Social work case management in medical settings.** In *Social workers' desk reference.* Edited by Roberts AR, Gilbert JG. Oxford: University Press; 2002:497–501.
41. Carr D: **Implications for case management: Ensuring access and delivery of quality health care to undocumented immigrant populations.** *Lippincott's Case Management* 2006, **11**(4):195–206.
42. Netzwerk Case Management Schweiz: *Definition and standards of Case Management.* Luzern; 2006.
43. Walcher M: **Das konzept case management und seine anwendung.** *Education Permanente* 2009, **4**:12–13.
44. Gelberg L, Andersen RM, Leake BD: **The behavioral model for vulnerable populations: application to medical care use and outcomes for homeless people.** *Health Serv Res* 2000, **34**(6):1273–1302.
45. Marmot M, Friel S, Bell R, Houweling TA, Taylor S: **Closing the gap in a generation: health equity through action on the social determinants of health.** *Lancet* 2008, **372**(9650):1661–1669.
46. Bieler G, Paroz S, Faouzi M, Trueb L, Vaucher P, Althaus F, Daeppen JB MD, Bodenmann P: **Social and medical vulnerability factors of emergency department frequent users in a universal health insurance system.** *Acad Emerg Med* 2012, **19**(1):63–68.
47. Schulz KF, Altman DG, Moher D and the CONSORT group: **CONSORT 2010 statement: updated guidelines for reporting parallel group randomized trials.** *Ann Intern Med* 2010, **152**(11):726–732.
48. Berkman ND, Sheridan SL, Donahue KE, Halpern DJ, Crotty K: **Low health literacy and health outcomes: an updated systematic review.** *Ann Intern Med* 2011, **155**(2):97–107.
49. Chew LD, Griffin JM, Partin MR, Noorbaloochi S, Grill JP, Snyder A, Bradley KA, Nugent SM, Baines AD, Vanryn M: **Validation of screening questions for limited health literacy in a large VA outpatient population.** *J Gen Intern Med* 2008, **23**(5):561–566.
50. Herndon JB, Chaney M, Carden D: **Health literacy and emergency department outcomes: a systematic review.** *Ann Emerg Med* 2011, **57**(4):334–345.
51. Drummond MF, Jefferson TO: **Guidelines for authors and peer reviewers of economic submissions to the BMJ: the BMJ economic evaluation working party.** *BMJ* 1996, **313**(7052):275–283.
52. Brooks R: **EuroQol: the current state of play.** *Health Policy* 1996, **37**(1):53–72.
53. Hudelson P, Kolly V, Perneger T: **Patients' perceptions of discrimination during hospitalization.** *Health Expect* 2010, **13**(1):24–32.
54. Gunnarsdottir OS1, Rafnsson V: **Mortality of the users of a hospital emergency department.** *Emerg Med J* 2006, **23**(4):269–273.
55. Billings J, Raven MC: **Dispelling an urban legend: frequent emergency department users have substantial burden of disease.** *Health Aff (Millwood)* 2013, **32**(12):2099–2108.
56. Althaus F, Stucki S, Guyot S, Trueb L, Moschetti K, Daeppen JB, Bodenmann P: **Characteristics of highly frequent users of a Swiss academic emergency department: a retrospective consecutive case series.** *Eur J Emerg Med* 2013, **20**(6):413–419.
57. Pines JM, Asplin BR, Kaji AH, Lowe RA, Magid DJ, Raven M, Weber EJ, Yealy DM: **Frequent users of emergency department services: gaps in knowledge and a proposed research agenda.** *Acad Emerg Med* 2011, **18**(6):e64–e69.
58. Raven MC: **What we don't know may hurt us: interventions for frequent: emergency department users.** *Ann Emerg Med* 2011, **58:**53–55.
59. Sanchez B, Hirzel AH, Bingisser R, Ciurea A, Exadaktylos A, Lehmann B, Matter H, Meier K, Osterwalder J, Sieber R, Yersin B, Camargo CA Jr, Hugli O: **State of emergency medicine in Switzerland: a national profile of emergency departments in 2006.** *Int J Emerg Med* 2013, **6**(1):23.

doi:10.1186/1472-6963-14-264
Cite this article as: Bodenmann *et al.*: Case management for frequent users of the emergency department: study protocol of a randomised controlled trial. *BMC Health Services Research* 2014 **14**:264.

Submit your next manuscript to BioMed Central and take full advantage of:

- **Convenient online submission**
- **Thorough peer review**
- **No space constraints or color figure charges**
- **Immediate publication on acceptance**
- **Inclusion in PubMed, CAS, Scopus and Google Scholar**
- **Research which is freely available for redistribution**

Submit your manuscript at
www.biomedcentral.com/submit

BioMed Central

Case Management may Reduce Emergency Department Frequent use in a Universal Health Coverage System: a Randomized Controlled Trial

Patrick Bodenmann, MD, MSc[1], Venetia-Sofia Velonaki, PhD[2], Judith L. Griffin, MD[1], Stéphanie Baggio, PhD[3], Katia Iglesias, PhD[4,5], Karine Moschetti, PhD[5,6,7], Ornella Ruggeri, Psych D[8], Bernard Burnand, MD MPH[5], Jean-Blaise Wasserfallen, MD MPP[6], Francis Vu, MD[1], Joelle Schupbach, RN[1], Olivier Hugli, MD MPH[9], and Jean-Bernard Daeppen, MD[10]

[1]Vulnerable Populations Center, Department of Ambulatory Care and Community Medicine, University of Lausanne, Lausanne, Switzerland; [2]Institute of Higher Education and Research in Healthcare, Department of Community Medicine and Public Health, Lausanne University Hospital and University of Lausanne, Lausanne, Switzerland; [3]Life Course and Social Inequality Research Center, Faculty of Social and Political Sciences, University of Lausanne, Lausanne, Switzerland; [4]Center for the Understanding of Social Processes, University of Neuchâtel, Neuchâtel, Switzerland; [5]Institute of social and preventive medicine, Lausanne University Hospital, Lausanne, Switzerland; [6]Technology Assessment Unit, Lausanne University Hospital, Lausanne, Switzerland; [7]IEMS - Plateforme interfacultaire en économie et management de la santé, University of Lausanne, Lausanne, Switzerland; [8]Department of Community Medicine and Public Health, Lausanne University Hospital, Lausanne, Switzerland; [9]Emergency Department, Lausanne University Hospital, Lausanne, Switzerland; [10]Alcohol Treatment Center, Lausanne University Hospital, Lausanne, Switzerland.

BACKGROUND: Frequent emergency department (ED) users account for a disproportionately high number of ED visits. Studies on case management (CM) interventions to reduce frequent ED use have shown mixed results, and few studies have been conducted within a universal health coverage system.

OBJECTIVE: To determine whether a CM intervention—-compared to standard emergency care—reduces ED attendance.

DESIGN: Randomized controlled trial.

PARTICIPANTS: Two hundred fifty frequent ED users (5 or more visits in the prior 12 months) who visited a public urban ED at the Lausanne University Hospital between May 2012 and July 2013 were allocated to either an intervention ($n = 125$) or control ($n = 125$) group, and monitored for 12 months.

INTERVENTIONS: An individualized CM intervention consisting of concrete assistance in obtaining income entitlements, referral to primary or specialty medical care, access to mental health care or substance abuse treatment, and counseling on at-risk behaviors and health care utilization (in addition to standard care) at baseline and 1, 3, and 5 months.

MAIN MEASURES: We used a generalized linear model for count data (negative binomial distribution) to compare the number of ED visits during the 12-month follow-up between CM and usual care, from an intention-to-treat perspective.

KEY RESULTS: At 12 months, there were 2.71 (±0.23) ED visits in the intervention group versus 3.35 (±0.32) visits among controls (ratio = 0.81, 95 % CI = 0.63; 1.02). In the multivariate model, the effect of the CM intervention on the number of ED visits approached statistical significance ($b = -0.219$, $p = 0.075$). The presence of poor social determinants of health was a significant predictor of ED use in the multivariate model ($b = 0.280$, $p = 0.048$).

CONCLUSIONS: CM may reduce ED use by frequent users through an improved orientation to the health care system. Poor social determinants of health significantly increase use of the ED by frequent users.

KEY WORDS: case management; vulnerable populations; utilization; clinical trials.

J Gen Intern Med 32(5):508–15
DOI: 10.1007/s11606-016-3789-9
© Society of General Internal Medicine 2016

Trial registration *ClinicalTrials.gov Identifier: NCT01934322*

Electronic supplementary material *The online version of this article (doi:10.1007/s11606-016-3789-9) contains supplementary material, which is available to authorized users.*

Received January 14, 2016
Revised May 4, 2016
Accepted June 21, 2016
Published online July 11, 2016

INTRODUCTION

Frequent emergency department (ED) users account for 3 to 8 % of all patients and 12 to 28 % of all ED visits,[1,2] contributing to overcrowding.[3] Common reasons for such frequent use include pain, chronic physical and mental illness, and substance abuse.[1,2,4,5] Frequent ED users are mainly men, are between 40 and 50 years of age, are sicker and have higher rates of mortality than occasional ED users.[1,2,6] As such, they merit focused attention, and research on interventions to meet their needs is needed.[6]

Case management (CM) is an intervention designed to assist frequent ED users in reducing their ED utilization.[7,8] CM aims to meet patients' individual needs and to optimize resource allocation for the frequent user and payer.[9,10] To our knowledge, only

three randomized controlled trials (RCTs) have examined the impact of CM on ED use.[11–13] Two RCTs[12,13] found that CM reduced the number of ED visits among frequent users, while the third[11] found no significant impact. A randomized information-sharing intervention did not result in a significant reduction in ED use.[14] Health care system characteristics and insurance coverage are factors that influence ED use,[15] and may explain discrepancies among these studies.

According to a study conducted in Switzerland,[16] frequent ED users accounted for 4.4 % of all ED patients and 12.1 % of all ED visits at the Lausanne University Hospital in 2008–2009. Like the majority of developed countries (91 % of OECD member nations),[17] Switzerland has universal health coverage, established in 1994. The system relies on mandatory individual health insurance, with government subsidies available, and less than 1 % of the population is uninsured.[18]

In response to calls for a unified definition of frequent ED use and primary care-based interventions[4], this RCT examined whether an interdisciplinary CM intervention, compared to standard emergency care, would reduce ED utilization among frequent users through an improved orientation to primary care and other community-based services within a universal health coverage system.

METHODS

Study design, setting and participants

Details on the study design and protocol were published in our previous work.[19] Briefly, we conducted an RCT with a parallel design to compare CM with standard care among frequent ED users of the Lausanne University Hospital (Switzerland) ED between May 2012 and July 2013. The Lausanne University Hospital is one of five EDs in the canton (state) of Vaud, and serves 770,000 people, with over 35,000 ED visits annually.[20] We defined frequent ED users as those who made five or more ED visits during the prior 12 months, including the index visit, using a validated definition.[1] Participants were randomized to the CM intervention or control group, and were monitored over 12 months. The primary outcome was the number of ED visits made by participants over the 12 month follow-up.

Participants were required to be at least 18 years of age and able to communicate in any language spoken by the team (French, English, Spanish, German, or Italian) or through a professional interpreter. Patients were excluded from the study if they 1) were unable to give informed consent, 2) planned to stay in Switzerland less than 18 months, 3) were not expected to survive at least 18 months (based on clinical judgment of research team, with systematic, proactive input from clinical providers, e.g. cardiologists or oncologists), 4) were awaiting incarceration or currently incarcerated, 5) had already received CM services, or 6) had a family member already enrolled in the study.

The trial was approved by the Human Research Ethics Committee of the Canton of Vaud, Switzerland (no. 32/12), and all participants provided written informed consent. The trial was funded by the Swiss National Science Foundation (no. 32003B_135762) and was registered on ClinicalTrials.gov (NCT01934322).

Sample Size

Based on results from a systematic review of the literature,[7] the sample size estimate was calculated to detect an average difference of two ED visits annually between the two groups (i.e. four fewer intervention group visits compared to two fewer control group visits, with an anticipated standard deviation of four in both groups). Eighty-five participants were needed in both groups using a significance level of 0.05 and power of 0.9. We anticipated a dropout rate of 30 %, based on the increased mortality rate of frequent ED users,[21]past research[19,22] and clinical experience of the CM team (serving populations including forced migrants and homeless persons), due to the instability in this population. Thus, we aimed to enroll 250 frequent ED users (125 in each group).

Recruitment, randomization, allocation and blinding

We identified frequent users using a continuous automated detection system linked with ED patient tracking software. Study staff provided frequent users with oral and written information about the study. Due to pragmatic constraints (e.g. after hours; simultaneous participants), the single research nurse was not able to approach all eligible frequent users. If a frequent user left the ED prior to contact with the study staff, a team member attempted to reach him/her by telephone up to three times within 24–72 hours, to explain the study and schedule a meeting. If a frequent user declined to enroll, we asked an open-ended question on the reason for declining. With the participants' permission, a CM team member contacted their primary care physician (PCP), if present, to inform him/her about the study and gather information.

Randomization was computer-generated and concealed from patients.[19] The research nurse, CM team, ED staff and data collection manager were not blinded to participant allocation, due to their activities and contacts. We informed study participants that they might receive CM services, without informing them of their group allocation. The statistician was blinded until the analyses were completed.

The CM team administered the intervention for 6 months following enrollment (until January 2014); patients were followed during the 6-month intervention and for an additional 6 months, for a total of 12 months (through July 2014).

CM intervention and control groups

In addition to standard emergency care, participants in the intervention group received the CM intervention at baseline and at 1, 3, and 5 months (Online Appendix 1). The baseline visit lasted 1.5 h, and follow-up visits took 30–60 min. An interdisciplinary mobile team consisting of four nurse practitioners and a chief resident[23] provided the intervention in an ambulatory care, hospital, or home setting. With our "open-door policy," participants were given the telephone number and address of the CM team and could make contact between scheduled appointments.

The CM team provided individualized services to each participant in the intervention group, emphasizing care coordination and facilitating communication between health care team members. Specifically, CM team members provided counseling, based on motivational interviewing and cross-cultural competences, on substance abuse (if applicable) and use of medical services. After assessing individual participant needs, we offered assistance to obtain income entitlements, improved housing (e.g. homeless shelters or asylum seeker housing), health insurance, domestic violence support and educational opportunities, to address these social determinants of health (SDH). Referrals were made to mental health services, substance abuse treatment or a new PCP on a case-by-case basis. As part of the CM intervention, we created a comprehensive care plan (Online Appendix 2) with practical recommendations for all of the participants' health care providers (PCP, psychiatrist, etc.). A key element of the intervention was establishing a link between providers and services at the hospital and community levels, promoting care continuity and improved orientation in the health care system.

Control group participants received only standard emergency care, but also met with a researcher during the 12 month follow-up (at 2, 5.5, 9 and 12 months), completing questionnaires related to outcomes which are not the focus of this paper (e.g. quality of life and the perception of discrimination[24]). Control group participants also received the CM team contact information, and anyone who contacted the team was eligible to receive CM services after the study.

Study Data and Outcome Measures

The primary outcome (number of ED visits) was obtained via the Lausanne hospital/ambulatory electronic records system and hospital/ambulatory administrative databases for each participant during the 12 months prior to and 12 months following enrollment.

Using validated standardized scales at baseline, we collected data on patient sociodemographic characteristics, SDH (including Medical Outcomes Study [MOS] survey[25] and subjective social status[26]), somatic (Charlson comorbidity index[27]) and mental health factors (Patient Health Questionnaire [PHQ][28], Mini-International Neuropsychiatric Interview [M.I.N.I.][29]), at-risk behaviors (Alcohol, Smoking and Substance Involvement Screening Test [ASSIST][30]), and health care utilization.[22]

Statistical Analysis

Statistical analyses were performed using STATA software (version 14; StataCorp LP, College Station, TX, USA), with the significance level set at $p = 0.05$. All analyses followed intention-to-treat standards. Descriptive statistics were computed using means and standard deviations for continuous variables, and absolute frequencies and percentages for categorical variables. We applied a generalized linear model for count data (negative binomial distribution) using the number of ED visits during the 12 months following enrollment as the dependent variable. We included an offset variable (corresponding to the logarithm of survival time) to account for participants who died during the study. First, we performed bivariate analyses to test the effect of the participant group (intervention or control), the number of visits at baseline (12 months before enrollment), age, gender, education, citizenship, French proficiency, PCP, somatic, mental and social determinants, and at-risk behaviors as independent variables on the use of ED services during the 12 month follow-up. Second, we ran a stepwise regression including all these independent variables in order to select the predictive variables ($p = 0.10$) to be included in the multivariate model. Ratio and 95 % confidence intervals were computed to estimate the effect size.

RESULTS

Of the 1145 frequent ED users identified during the recruitment period, we could not approach 217 (Fig. 1) due to pragmatic constraints for the single research nurse recruiting during periods of heavy patient influx, and 231 did not meet eligibility criteria. We were unable to contact 171 (after initial contact in the ED, they did not respond to follow-up calls), and 276 refused to participate. Reasons for declining included no expected benefit, not being satisfied with the hospital, and recent participation in another study. Those who refused did not differ in sex or nationality, but were older than enrolled participants (52.3 vs. 48.6 years old, $p = 0.03$). Overall, 250 (47.5 %) agreed to participate and were allocated to the intervention ($n = 125$) or control group ($n = 125$).

Participant Characteristics (Table 1)

The mean age of the participants was 48.5 years (±18.9), and 57.2 % were men. The intervention group had significantly lower educational attainment than controls. Participants reported high levels of poor SDH, including inadequate housing, lack of employment, and problems with immigration status. The majority suffered from a chronic condition, medical co-morbidity or psychiatric illness, and a third reported at-

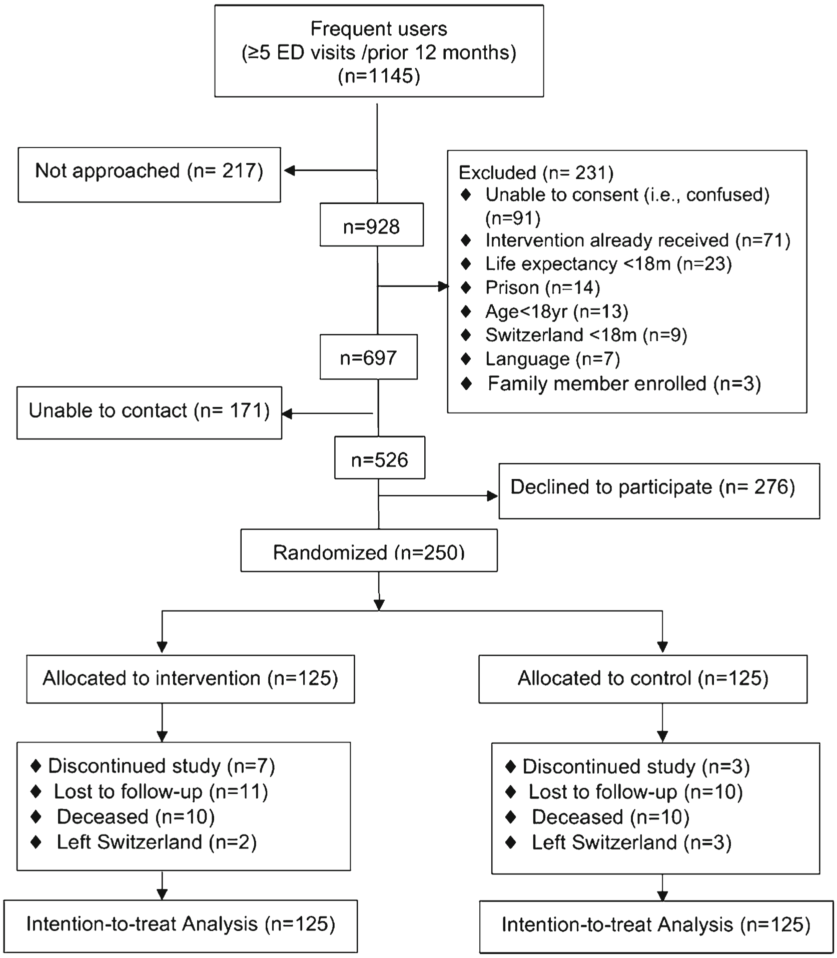

Figure 1 Enrollment flow chart.

risk behaviors. Only 14 % did not have a PCP. The groups had an equal number of ED visits in the 12 months prior to enrollment.

Study Implementation

All 125 intervention group participants received the intervention at baseline, 106 (84.8 %) at 1 month, 98 (78.4 %) at 3 months, and 93 (74.4 %) at 5 months; 108 (86.4 %) intervention group participants contacted the CM team between study visits. No control group participants contacted the CM team proactively. Twenty participants (10 in each group) died during the study.

The CM team referred 66 participants (52.8 %) to mental health professionals and 34 (27.2 %) to substance abuse treatment. Sixty participants (48.0 %) received additional social services, and 83 (66.4 %) were referred to specialized medical doctors.

Outcome (Table 2)

During the 12 month follow-up, control group participants made an average of 3.35 ± 0.32 ED visits, whereas intervention group participants made 2.71 ± 0.23 visits, corresponding to 19 % fewer ED visits (ratio = 0.81, 95 % CI = 0.63 to1.02).

The effect of the CM intervention (i.e. group) on the number of ED visits was not statistically significant in the bivariate model, ($b = -0.217$, $p = 0.080$) (Table 2). The association between social determinants and the number of visits approached statistical significance ($b = 0.272$, $p = 0.055$), with poor SDH being associated with higher ED use, in the bivariate model. Group assignment (intervention or control) and social determinants were used in the stepwise multivariate regression. In this model, the effect of the group approached significance ($b = -0.219$, $p = 0.075$), with the intervention group making fewer ED visits compared to the control group. The presence of poor SDH was significant in the final model ($b = 0.280$, $p = 0.048$).

DISCUSSION

In this randomized controlled trial, a CM intervention led to 19 % fewer ED visits by frequent users, which approached statistical significance, through an improved orientation to and coordination of services within the health care system. Our results also demonstrate that the presence of poor SDH—including social isolation, housing instability, or financial insecurity—was associated with higher ED use among frequent users.

Table 1 Baseline Characteristics of the Study Population

	Total (*n* = 250)	Intervention group (*n* = 125)	Control group (*n* = 125)
Sociodemographic characteristics (%, *n*)			
Male	57.2 (143)	56.0 (70)	58.4 (73)
Age (mean, SD)	48.5(18.9)	48.4 (18.7)	48.6 (19.1)
Citizenship			
Switzerland	47.6 (119)	46.4 (58)	48.8 (61)
Europe	17.6 (44)	19.2 (24)	16.0 (20)
Other (e.g. Africa, Asia, Lat Am)	34.5 (86)	34.4 (43)	34.7 (43)
Education			
High school/vocational school	45.2 (113)	39.2 (49)	51.2 (64)
University/College	16.8 (42)	13.6 (17)	20.0 (25)
Compulsory school only, do not know or other	38.0 (95)	47.2 (59)	28.8 (36)
Uninsured	2.8 (7)	2.4 (3)	3.2 (3)
Limited French proficiency	18.8 (47)	18.4 (23)	19.2 (24)
No Primary care physician	14.0 (35)	16.0 (20)	12.0 (15)
Number of ED visits (mean, SD)	6.2 (2.1)	6.1 (1.9)	6.2 (2.3)
5 (%, *n*)	54.8 (137)	51.2 (64)	58.4 (73)
6 (%, *n*)	20.8 (52)	24.0 (30)	17.6 (22)
7 (%, *n*)	9.2 (23)	10.4 (13)	8.0 (10)
8 (%, *n*)	6.0 (15)	5.6 (7)	6.4 (8)
9 (%, *n*)	3.6 (9)	4.8 (6)	2.4 (3)
10 or more (10–24) (%, *n*)	5.6 (14)	4.0 (5)	7.2 (9)
Social determinants (any) (%, *n*)*	72.8 (182)	74.4 (93)	71.2 (89)
Complex family situation	43.6 (109)	45.6 (57)	41.6 (52)
Social isolation	31.2 (78)	30.4 (38)	32.0 (40)
Financial hardship	49.6 (124)	50.4 (63)	48.8 (61)
Inadequate housing (homeless or refugee housing)	24.0 (60)	26.4 (33)	21.6 (27)
Lack of employment or other activities	50.4 (126)	54.4 (68)	46.4 (58)
Limited French proficiency	16.0 (40)	12.8 (16)	19.2 (24)
Problem with immigration status	22.0 (55)	24.0 (30)	20.0 (25)
Somatic determinants (any) (%, n)†	69.2 (173)	69.6 (87)	68.8 (86)
Chronic and/or acute severe illness	59.2 (148)	61.6 (77)	56.8 (71)
Comorbidity	23.2 (58)	24.0 (30)	22.4 (28)
Polypharmacy	17.2 (43)	20.0 (25)	14.4 (18)
Treatment non-adherence	6.4 (16)	8.8 (11)	4.0 (5)
Mental determinants (any) (%, *n*)[‡]	50.8 (127)	47.2 (59)	54.4 (68)
Depression	27.2 (68)	23.2 (29)	31.2 (39)
Anxiety disorder	31.6 (79)	28.8 (36)	34.4 (43)
Personality disorder	6.0 (15)	4.8 (6)	7.2 (9)
Psychotic disorder	3.2 (8)	2.4 (3)	4.0 (5)
At-risk behaviors (any) (%, *n*)[§]	32.0 (80)	34.4 (43)	29.6 (37)
Alcohol use	27.6(69)	28.8 (36)	26.4 (33)
Tobacco use	29.6 (74)	32.0 (40)	27.2 (34)
Illicit drug use	10.8 (27)	11.2 (14)	10.4 (13)

**MOS Social Support Survey*[25] *and subjective social support survey*[26]
[†]*Charlson score*[27]
[‡]*PHQ*[28] *and M.I.N.I.*[29]
[§]*ASSIST*[30]

While our main results do not achieve statistical significance, 19 % fewer ED visits is clinically relevant, given the significant time and resources required to care for frequent ED users.[31–33] For example, in the USA (21-28 % of 130 million total visits), a reduction of the magnitude found in our study would translate into 5.1–6.8 million avoided visits annually.[15,34] The non-significant reduction in ED use found in this study underscores the mixed evidence in the literature. At least seven prior studies[12,13,35–39] showed ED use reductions following a CM or similar intervention, while five studies[9,11,14,40,41] did not. In terms of study design, sample size and intervention (i.e. in-person CM intervention), our trial most closely matches that of Shumway,[12] who found an additional reduction of one ED visit. A 1997 RCT did not find a reduction in number of ED visits following a CM-like intervention[11]; however, they defined frequent use as greater than 10 annual ED visits, and thus their results may be difficult to compare to our own. Two RCTs conducted in Sweden used a lower threshold to define ED frequent use (>3 visits), and implemented interventions different from ours.[13,14] Differences in the definition of frequent use and in intervention design and setting may have contributed to these varying results. Our results may have been influenced by the fact that despite our use of a validated definition,[1] most participants had only 5–6 visits at enrollment, and CM may be of greater benefit for those with higher baseline ED use, given the increased vulnerability of this group.[12,42] Furthermore, over one-third of participants were from Africa, Latin America or Asia, regions of origin common for asylum seekers, refugees or undocumented immigrants living in Switzerland. The limited primary care services in these regions[43] may have led to increased ED use among these participants. Finally, significantly lower education among intervention group participants may have increased ED use in this group.[44]

Table 2 Bivariate and Multivariate Models Predicting Number of ED Visits at Follow-Up

Variables	Bivariate models[a]		Multivariate final model[a,b]	
	b[c]	p value	*b*	p value
Intervention group[d]	−0.217	0.080	−0.219	0.075
Number of ED visits at enrollment (prior 12 months)	0.026	0.333	–	–
Male[e]	−0.119	0.342	–	–
Age	−0.003	0.416	–	–
Citizenship[f]				
Europe	−0.063	0.717	–	–
Other (e.g. Africa, Asia, Lat Am)	−0.022	0.872	–	–
Education[g]				
High school/vocational school	0.080	0.606	–	–
University/college	−0.050	0.799	–	–
Do not know/other	0.164	0.442	–	–
Limited French proficiency	−0.073	0.442	–	–
No primary care physician	−0.257	0.166	–	–
Social determinants[h]	0.272	0.055	0.280	0.048
Somatic determinants[i]	0.078	0.562	–	–
Mental determinants[j]	0.169	0.172	–	–
At-risk behaviors[k]	−0.003	0.980	–	–

[a]*Generalized linear model for count data (negative binomial distribution)*
[b] *Stepwise regression (p = 0.10)*
[c] *"b" is the coefficient of the regression model*
[d]*Reference category: controls*
[e]*Reference category: female*
[f] *Reference category: Swiss nationality*
[g]*Reference category: compulsory school only*
[h]*Social determinants (at least one determinant): complex family situation, social isolation, financial hardship, inadequate housing, lack of employment, limited French proficiency, problems with immigration status*
[i]*Somatic determinants (at least one determinant): chronic and/or acute severe illness, comorbidity, polypharmacy, treatment non-adherence*
[j]*Mental determinants (at least one determinant): depression, anxiety, personality disorder, psychotic disorder*
[k]*At-risk behaviors (at least one determinant): alcohol use, tobacco use, Illicit drug use*

Another important consideration is that the number of ED visits decreased in both groups. Contact between control group participants and the research team may have introduced contamination bias, contributing to a reduction in ED use among controls. However, despite receiving information about the CM team at enrollment, no control group participant proactively contacted the team to seek out services. A second explanation is that ED use becomes less frequent over time (i.e. regression to the mean), even without intervention.[7,19] Finally, the Hawthorne effect—that people have a tendency to change their behavior when under observation—may have influenced ED use among these participants. In the Reinius study,[13] control participants were passively observed in a Zelen's design, adopted in part to avoid a Hawthorne effect.

This pragmatic RCT has several limitations and strengths. First, we conducted this study at a single site, the sole tertiary care center in the canton of Vaud and one of five academic medical centers in Switzerland. However, in order to maximize the generalizability of our findings, we recruited a representative study sample of frequent ED users.[16,45] In addition, the design of the Swiss health system—privatized but with universal coverage—allows for generalization of our findings to North America, Europe and parts of Asia. Second, the enrollment rate of 47.5 % could have biased or contributed to our non-significant results. This suggests that CM services may not appeal to some frequent users, who may benefit from alternative outreach strategies. However, this enrollment rate is comparable to those of other studies,[13] and we recruited an adequate number of participants based on power calculations. Although we anticipated a dropout rate of 30 %, we retained 78 % of study participants. Our intention-to-treat analysis also reflects a "real world" scenario of caring for this highly vulnerable population. Third, we were unable to track the ED use of participants who visited an outside hospital or moved out of the area. Fourth, our small but experienced team was unable to approach 217 individuals during recruitment and were not blinded to allocation given their role in delivering the intervention; thus we cannot exclude a possible selection bias, despite specifically instructing our team against this. Fifth, excluding frequent users who had previously received CM services may have impacted our results. However, the characteristics of the frequent users we enrolled were qualitatively similar to participants in previous studies,[16,22] suggesting that we recruited a representative sample. Finally, the 12-month study duration may have limited our ability to demonstrate the full scope of the benefit (or lack thereof) over a longer period. However, Shumway[12] performed sensitivity analyses demonstrating similar cost-effectiveness of a CM intervention at 12 months and 24 months, suggesting that 1 year may be an appropriate study length.

Evidence regarding the impact of the CM on ED use remains inconclusive. A key goal of this CM intervention was to offer improved orientation and redirection to a range of hospital and community-based services. While most participants already had a PCP, caring for these highly vulnerable patients independently in the community is challenging. The

main contribution of this intervention was to facilitate and coordinate care of frequent users, with the PCP integrated into this approach. The development of effective and efficient strategies to improve care for frequent users of the ED and other health services is an area of great interest. CM could serve as a link between disparate parts of complex health systems, with the PCP as the nexus for care continuity. CM teams should focus on modifiable SDH—such as housing or employment—in addition to traditional biomedical risk factors. Research investigating the impact of CM on specific highly vulnerable frequent users, such forced migrants or those with low health literacy, is warranted. Future research should explore patient-reported outcomes, and analyze costs at the institutional and community levels, taking into account the long-term needs of patients.

Acknowledgments:

This study was funded by the by the Swiss National Science Foundation (no: 32003B_135762).
The authors wish to thank the research team–Séverine Alary, Jolanta Nobs, and Sarah Kahnt–and the CM team (Corine Ansermet, Marina Canepa-Allen, and Laetitia LeNocher) for their contribution to the collection of the data and the medical care provided to the frequent users of the emergency department. We would also like to acknowledge Valentin Rousson for his invaluable guidance regarding our methodology and Phillipe Staeger for his clinical contributions to this study.

Prior Presentations:: *Preliminary study findings were presented at the following:*
• Society of General Internal Medicine (SGIM) 38th Annual Meeting, April 22–25, 2015, Toronto, ON, Canada, oral presentation (plenary session)
• Swiss Society of General Internal Medicine 83rd Annual Congress, May, 20–22, 2015, Basel, Switzerland (oral presentation, 2nd prize among oral presentations)

Corresponding Author: *Patrick Bodenmann, MD, MSc; Vulnerable Populations Center, Department of Ambulatory Care and Community MedicineUniversity of Lausanne, Lausanne, Switzerland (e-mail: Patrick.Bodenmann@hospvd.ch).*

Compliance with Ethical Standards:

Conflict of Interest: *The authors declare that they do not have a conflict of interest.*

REFERENCES

1. **Locker TE, Baston S, Mason SM, Nicholl J.** Defining frequent use of an urban emergency department. Emerg Med J. 2007;24(6):398–401.
2. **van Tiel S, Rood PP, Bertoli-Avella AM, et al.** Systematic review of frequent users of emergency departments in non-US hospitals: state of the art. Eur J Emerg Med. 2015.
3. **Hoot NR, Aronsky D.** Systematic review of emergency department crowding: causes, effects, and solutions. Ann Emerg Med. 2008;52(2):126–136.e121.
4. **Pines JM, Asplin BR, Kaji AH, et al.** Frequent users of emergency department services: gaps in knowledge and a proposed research agenda. Acad Emerg Med. 2011;18(6):e64–e69.
5. **Vu F, Daeppen J-B, Hugli O, et al.** Screening of mental health and substance users in frequent users of a general Swiss emergency department. BMC Emerg Med. 2015;15(1):27.
6. **Moe J, Kirkland S, Ospina MB, et al.** Mortality, admission rates and outpatient use among frequent users of emergency departments: a systematic review. Emerg Med J. 2015. doi:10.1136/emermed-2014-204496.
7. **Althaus F, Paroz S, Hugli O, et al.** Effectiveness of interventions targeting frequent users of emergency departments: a systematic review. Ann Emerg Med. 2011;58(1):41–52.e42.
8. **Kumar GS, Klein R.** Effectiveness of case management strategies in reducing emergency department visits in frequent user patient populations: a systematic review. J Emerg Med. 2013;44(3):717–729.
9. **Lee K-H, Davenport L.** Can case management interventions reduce the number of emergency department visits by frequent users? Health Care Manag. 2006;25(2):155–159.
10. Case Management Society of America. What is a case manager? 2008–11; http://www.cmsa.org/Home/CMSA/WhatisaCaseManager/tabid/224/Default.aspx. Accessed May 30 2016.
11. **Spillane LL, Lumb EW, Cobaugh DJ, Wilcox SR, Clark JS, Schneider SM.** Frequent users of the emergency department: can we intervene? Acad Emerg Med. 1997;4(6):574–580.
12. **Shumway M, Boccellari A, O'Brien K, Okin RL.** Cost-effectiveness of clinical case management for ED frequent users: results of a randomized trial⋆. Am J Emerg Med. 2008;26(2):155–164.
13. **Reinius P, Johansson M, Fjellner A, Werr J, Öhlén G, Edgren G.** A telephone-based case-management intervention reduces healthcare utilization for frequent emergency department visitors. Eur J Emerg Med. 2013;20(5):327–334.
14. **Hansagi H, Olsson M, Hussain A, Öhlén G.** Is information sharing between the emergency department and primary care useful to the care of frequent emergency department users? Eur J Emerg Med. 2008;15(1):34–39.
15. **LaCalle E, Rabin E.** Frequent users of emergency departments: the myths, the data, and the policy implications. Ann Emerg Med. 2010;56(1):42–48.
16. **Bieler G, Paroz S, Faouzi M, et al.** Social and medical vulnerability factors of emergency department frequent users in a universal health insurance system. Acad Emerg Med. 2012;19(1):63–68.
17. Organization for Economic Co-operation and Development (OECD). OECD Health Data: Social protection. 2013; http://www.oecd-ilibrary.org/social-issues-migration-health/data/oecd-health-statistics/oecd-health-data-social-protection_data-00544-en. Accessed May 30,2016.
18. **Leu RE RF, Brouwer W, Matter P, Rütschi C**. The swiss and dutch health insurance systems: Universal coverage and regulated competitive insurance markets. Website of the Commonwealth Fund: Commonwealth Fund 2009.
19. **Bodenmann P, Velonaki V-S, Ruggeri O, et al.** Case management for frequent users of the emergency department: study protocol of a randomised controlled trial. BMC Health Serv Res. 2014;14(1):264.
20. **Sanchez B, Hirzel AH, Bingisser R, et al.** State of emergency medicine in Switzerland: a national profile of emergency departments in 2006. Int J Emerg Med. 2013;6(1):1–11.
21. **Rafnsson V, Gunnarsdottir OS.** Mortality of the users of a hospital emergency department. Emerg Med J. 2006;23(4):269–273.
22. **Bodenmann P, Baggio S, Iglesias K, et al.** Characterizing the vulnerability of frequent emergency department users by applying a conceptual framework: a controlled, cross-sectional study. Int J Equity Health. 2015;14(1):146.
23. **Canepa Allen MCAC, Schüpbach J, Vu F, Bouche L, Ninane F, Bodenmann P.** Respectful nursing support of patient priorities. Krankenpfl Soins Infirm. 2014;107(6):62–65.
24. **Baggio S, Iglesias K, Hugli O, et al.** Associations between perceived discrimination and health status among frequent Emergency Department users. Eur J Emerg Med. 2015.
25. **Sherbourne CD, Stewart AL.** The MOS social support survey. Soc Sci Med. 1991;32(6):705–714.
26. **Singh-Manoux A, Adler NE, Marmot MG.** Subjective social status: its determinants and its association with measures of ill-health in the Whitehall II study. Soc Sci Med. 2003;56(6):1321–1333.
27. **Charlson ME, Pompei P, Ales KL, MacKenzie CR.** A new method of classifying prognostic comorbidity in longitudinal studies: development and validation. J Chronic Dis. 1987;40(5):373–383.
28. **Spitzer RL, Kroenke K, Williams JB, PHQPCS Group.** Validation and utility of a self-report version of PRIME-MD: the PHQ primary care study. JAMA. 1999;282(18):1737–1744.
29. **Sheehan DV, Lecrubier Y, Sheehan KH, et al.** The Mini-International Neuropsychiatric Interview (MINI): the development and validation of a structured diagnostic psychiatric interview for DSM-IV and ICD-10. J Clin Psychiatry. 1998;59:22–33.
30. **World Health Organization ASSIST Working Group.** The alcohol, smoking and substance involvement screening test (ASSIST): development, reliability and feasibility. Addiction. 2002;97(9):1183–1194.
31. **Moore G, Gerdtz M, Manias E, Hepworth G, Dent A.** Socio-demographic and clinical characteristics of re-presentation to an Australian inner-city emergency department: implications for service delivery. BMC Public Health. 2007;7(1):320.

32. **Fuda KK, Immekus R.** Frequent users of Massachusetts emergency departments: a statewide analysis. Ann Emerg Med. 2006;48(1):16. e11–16. e18.
33. **Dent AW, Phillips GA, Chenhall AJ, McGregor LR.** The heaviest repeat users of an inner city emergency department are not general practice patients. Emerg Med. 2003;15(4):322–329.
34. National Hospital Ambulatory Medical Care Survey: 2011 Emergency Department Summary Tables. CDC;2011.
35. **Wassmer R, Winward L, Derlet R**. Does counseling reduce frequent emergency department use? 2008.
36. **Okin R, Boccellari A, Azocar F, et al.** The effects of clinical case management on hospital service use among ED frequent users. Am J Emerg Med. 2000;18(5):603–608.
37. **Pope D, Fernandes C, Bouthillette F, Etherington J.** Frequent users of the emergency department: a program to improve care and reduce visits. CMAJ. 2000;162(7):1017–1020.
38. **Grover CA, Close RJH, Villarreal K, Goldman LM.** Emergency department frequent user: pilot study of intensive case management to reduce visits and computed tomography. West J Emerg Med. 2010;11(4):336–343.
39. **Skinner J, Carter L, Haxton C.** Case management of patients who frequently present to a Scottish emergency department. Emerg Med J. 2009;26(2):103–105.
40. **Wexler R, Hefner JL, Sieck C, et al.** Connecting emergency department patients to primary care. J Am Board Fam Med. 2015;28(6):722–732.
41. **Phillips G, Brophy D, Weiland T, Chenhall A, Dent A.** The effect of multidisciplinary case management on selected outcomes for frequent attenders at an emergency department. Med J Aust. 2006;184(12):602–606.
42. **Doupe MB, Palatnick W, Day S, et al.** Frequent users of emergency departments: developing standard definitions and defining prominent risk factors. Ann Emerg Med. 2012;60(1):24–32.
43. **World Health Organization**. The World Health Report 2008: primary health care (now more than ever). 2014.
44. **Berkman ND, Sheridan SL, Donahue KE, Halpern DJ, Crotty K.** Low health literacy and health outcomes: an updated systematic review. Ann Intern Med. 2011;155(2):97–107.
45. **Althaus F, Stucki S, Guyot S, et al.** Characteristics of highly frequent users of a Swiss academic emergency department: a retrospective consecutive case series. Eur J Emerg Med. 2013;20(6):413–419.

CHUV | CENTRE HOSPITALIER UNIVERSITAIRE VAUDOIS

PMU| POLICLINIQUE MEDICALE UNIVERSITAIRE

Case Management for Frequent Users of the Emergency Department: A Randomized Controlled Trial

Patrick Bodenmann, MD, MSc
Jean-Bernard Daeppen, MD
Bernard Burnand, MD, MPH
Olivier Hugli, MD, MPH
Karine Moschetti, PhD
Philippe Staeger, MD, MPH
Jean-Blaise Wasserfallen MD, MPP

canton de vaud

Table des matières

1. TITRE DE L'ETUDE ... 3
2. DATE DE L'ENVOI DU PROTOCOLE ET DATE PREVUE POUR LE DEBUT DE L'ETUDE ... 3
3. NOM ET SIGNATURE DE L'INVESTIGATEUR RESPONSABLE ET DES CO-INVESTIGATEURS ... 3
4. MISE EN PERSPECTIVE DE L'ETUDE ... 4
A) STATE OF THE ART ... 4
B) HYPOTHESIS ... 7
C) AIM OF THE STUDY ... 8
D) OUTCOMES ... 8
E) RATIONAL ... 8
5. PLAN GENERAL ... 9
A) STUDY DESIGN ... 9
B) SETTING ... 9
C) PARTICIPANTS ... 9
D) INTERVENTIONS ... 10
d.1) The multidisciplinary mobile team CM pattern intervention ... 10
d.2) The control intervention ... 12
E) OUTCOMES ... 12
e.1) Primary outcome: number of ED visits ... 12
e.2) Secondary outcome: costs analysis ... 13
e.3) Secondary outcome: standardized measure of health status via EuroQol (EQ-5D) ... 14
F) RANDOMIZATION, ALLOCATION ... 14
f.1) Sequence generation ... 14
f.2) Allocation concealment mechanism ... 14
f.3) Blinding ... 14
f.4) Statistical methods ... 14
6. SELECTION DES SUJETS ... 15
A) FLOW DIAGRAM ... 15
B) SAMPLE SIZE ... 16
C) PARTICIPANTS ... 16
7. DEROULEMENT DE L'ETUDE ET INVESTIGATIONS PREVUES ... 17
8. SURVEILLANCE MEDICALE ... 17
9. ROLE DU PERSONNEL INFIRMIER ... 17
10. MEDICAMENTS ... 17
11. EVALUATION DES RISQUES ... 17
12. COUVERTURE D'ASSURANCE (RC) ... 18
13. FORMULAIRES D'INFORMATION ET DE CONSENTEMENT ... 18
14. TRAITEMENT DE DONNEES PERSONNELLES ET D'ECHANTILLONS BIOLOGIQUES ... 18
15. PLAN DE FINANCEMENT ET RETRIBUTION : ... 18
16. ETUDE IMPLIQUANT LA PARTICIPATION DE PRATICIENS INSTALLES ... 19
17. INFORMATIONS AU PERSONNEL SOIGNANT MEDICAL ET PARAMEDICAL ... 19
18. REFERENCE LIST ... 20
ANNEXE 1 ... 24
ANNEXE 2 ... 28
ANNEXE 3 ... 36
ANNEXE 4 ... 39
ANNEXE 5 ... 42

1. Titre de l'étude

Case Management for Frequent Users of the Emergency Department: A Randomized Controlled Trial

2. Date de l'envoi du protocole et date prévue pour le début de l'étude

Date de l'envoi du protocole : 27 janvier 2012
(pour la séance de la Commission d'éthique du 7 février 2012)

Date prévue pour le début de l'étude : 1er avril 2012

3. Nom et signature de l'investigateur responsable et des co-investigateurs

Investigateur responsable:

Patrick Bodenmann, Médecin associé, MER, MSc

Co-investigateurs :

Jean-Bernard Daeppen, Professeur associé

Bernard Burnand, Professeur, MPH

Olivier Hugli, Médecin adjoint, PD-MER, MPH

Karine Moschetti, PhD en sciences économiques

Philippe Staeger, Médecin associé, MPH

Jean-Blaise Wasserfallen, Professeur titulaire, MPP

Services dont relèvent les patients prévus dans l'étude :

Département des centres interdisciplinaires et logistique médicale (DCILM)
Service des Urgences

Professeur Bertrand Yersin

Département universitaire de médecine et santé communautaire (DUMSC) Policlinique Médicale Universitaire (PMU)

Professeur Jacques Cornuz

Lieux où l'étude sera réalisée :

Service des urgences, CHUV

Secteur des urgences ambulatoires, PMU

4. Mise en perspective de l'étude

a) State of the art
b) Hypothesis
c) Aims
d) Outcomes
e) Rational

a) State of the art

Vulnerable patients and deficient care

It is recognized that poor social determinants of health (low socioeconomic status, lack of family resources, immigration status, inferior language skills, etc.) have negative effects on health[1-4]. In Switzerland, 9.0% of the working-age population live under the poverty threshold, with more than 3.0% benefiting from financial social help[5]. In addition, about 41,000 asylum seekers and 90,000[6] to 180,000[7] undocumented migrants are recorded. Risk behaviors also have negative impacts on health[1 8 9]. In Switzerland, about 30.0% of the adult population smoke tobacco, 60,000 use cocaine or heroin[10] and 300,000 people are alcohol-dependent.

In addition to poor health, individuals presenting social and/or behavioral risk factors are more likely to receive improper healthcare[11-13]. They can be defined as vulnerable, according to the literature referring to vulnerable patients, as social subgroups at increased risk of poor physical, psychological, social health and inadequate healthcare[11 14-16].

⇨ **Key issue 1 : individuals with social and/or behavioral risk factors are at risk of poor health and of receiving less than optimal healthcare**

Inadequate healthcare is related to the fact that vulnerability is usually shaped by many medical and social factors[11 14 17-19]. Existing facilities are not equipped to service multi-faceted needs[14] or to deal with the related complexity of the cases. In caring for vulnerable populations, someone who can centralize information and guide the patient through the entire care process is usually not available.

In Switzerland, increased poverty, illegal and forced migration, old age and decreased social supports suggest that the number of vulnerable patients is about to increase and put even greater demands on the healthcare system[14 18]. Taking care of a growing number of vulnerable patients becomes an institutional challenge, requiring specific evaluation and orientation measures as well as numerous specialized skills.

⇨ **Key issue 2: taking care of vulnerable patients is complex and requires specific interventions**

A subgroup of vulnerable patients: Emergency department frequent users (ED-FU)

Specific subgroups are identified as vulnerable patients in the medical literature (e.g. forced migrants, substance addicts, and the homeless[14 16 18 20]). FUs of hospital emergency departments (ED) are also one of these subgroups. Recent research suggests that just because these patients return to the ED repeatedly, it is not indicative of a system that is unable to properly treat illness and injury, or has a lack of adequate primary care facilities. Rather, it represents one that is not designed or equipped to accommodate or deal with the complex and

interrelated clinical, psychosocial and patient factors[21] it is confronted with by this sub-population.

ED-FU attend the unit on multiple occasions; however, definitions and number of visits thresholds vary across studies. According to Locker[22], the definition of five attendances or more per year corresponds to a non-random event and should be used to allow better comparisons between studies.

Many studies have established profiles of ED-FU. They present a higher rate of morbidity and mortality than less frequent ED users[21 23-26], are more at risk for drug and alcohol abuse[24 26-28], often present mental health issues[21 24 25 29], are more likely to visit for complications and exacerbations of chronic conditions[29 30] and are often homeless, uninsured and of low socio-economic level[21 31-33].

⇨ **Key issue 3 : ED-FU are identified as vulnerable patients regarding their multiple socio-medical demands on settings not designed to provide this specific type of care**

Those attending ED on a regular basis do not represent the majority of all ED patients, but they do account for a disproportionally high number of all ED visits. Hansagi et al.[34] found that 7% of patients visiting ED at least four times in 12 months accounted for 45% of the total number of ED visits. Locally in 2008-2009, FU accounted for 4.4% of ED patients and 12.1% (n= 5,813) of all ED visits (n= 48,117)[35].

ED-FU contribute significantly to ED overcrowding and extended waiting time, often due to inappropriate visits to the unit[36]. Overcrowding is detrimental to the quality of care in the ED. However, the severity of the reason for consultation at the ED is often controversial[37]. Indeed, several studies show that ED-FU have non-emergency conditions[29 38-40] and could receive better care in settings other than ED[41 42], which is not designed to provide continuous care to patients with non-emergency chronic conditions. In addition, the numerous issues that ED-FU have are not easily addressed by simply providing care only. Appropriate and consistent medical and social services are needed for vulnerable populations.

⇨ **Key issue 4 : ED-FU misuse the healthcare system and overuse ED**

<u>Specific interventions to the attention of ED-FU</u>

ED-FU need consistent medical services and social support. They tend to be more ill, their probability of hospital admission is greater, and they report subjectively poorer physical health. The majority of them believe that their complaints require immediate attention[37], thus they represent a significant burden on the hospital due to multiple visits and amount of problems they bring to the ED. In response to these concerns, several institutions worldwide (e.g. in the United States, Canada, Sweden, United Kingdom, the Netherlands, Spain and Australia)[28 31 43-53] have introduced specific interventions for ED-FU aimed at reducing their visits, treating their medical co-morbidities and/or addressing their social needs.

One of the most common interventions consists of case management (CM) multidisciplinary teams composed of nurses, psychologists and possibly physicians[49 54-59]; this approach can help address complex situations and scenarios. Team members of different professional backgrounds, such as psychologists, psychiatrists and health educators might complement the team, depending on the specific CM project. Coordination and organizational care tasks are often allocated to a case manager[57], who guides patients through the care process and provides social supports. Care is generally considered as a continuous integration of medical and social dimensions. It is commonly patient-centered and holistic in nature, and takes into account

patient empowerment[49 55 56]. Moreover, the locus of intervention is not limited to the hospital, and often extends into the community.

CM is a highly flexible and dynamic process and mainly depends on patient needs; the order of single steps is often not constricted. In fact, its dynamic condition emphasizes that sometimes several steps take place simultaneously, or that the case manager has to return to a previous step. Based on the literature, they can be summarized in five steps [49 58-63]: identification, assessment/reassessment, planning, implementation, and evaluation/ monitoring.

The Behavioural Model for Vulnerable Populations[16] provides a theoretical framework for understanding how CM might improve the care of vulnerable patients; this theoretical framework suggests that the use of health services is a function of:

- predisposition by patients (demographics, health beliefs, social structure and childhood characteristics),
- factors that enable or impede its use (personal, family or community resources), and
- patient need for care (perceived and evaluated health).

CM guarantees that issues in each of these domains are addressed.

⇨ **Key issue 5: Specific interventions, mostly inspired by CM and using adapted frameworks, have been introduced in different countries to improve the health and welfare of ED-FU.**

Interventions aiming at improving ED-FU management have had positive outcomes: some of the interventions evaluated have been effective in reducing emergency department use[28 31 43 46 48 51 53]. Cost-reduction analyses are also promising: Wassmer anticipated reductions in cost even when partially based on modeling estimates[53]; two other studies showed the effects of clinical case management on hospital services and its cost effectiveness[31 51]. Some interventions have had positive effects on social outcomes[31], such as a significant reduction of homelessness[47 51]. A positive effect on social outcome is essential, and the link between social problems and health has been demonstrated by many authors[2]. Finally, clinical outcomes were assessed in 3 studies[31 47 51]; one of them demonstrated a positive effect in reducing alcohol and drug use[31].

⇨ **Key issue 6: Evidence from international projects is promising: a number of interventions succeeded in decreasing ED visits, were cost-effective and had positive impacts on some social and clinical outcomes.**

<u>Local pilot studies</u>

ED-FU are vulnerable patients

At the Lausanne University Hospital ED, a retrospective chart review case-control study[35] conducted between April 2008 and March 2009, demonstrated that social (i.e. homelessness, institutionalization, unemployment and dependence on government welfare) and specific medical vulnerability factors (i.e. ED primary diagnosis of substance abuse and the use of five or more clinical departments in 12 months) increased the risk of ED use among 719 patients. A combination of social and medical factors was markedly associated with ED frequent use, as frequent users were 10 times more likely to have three of them (on a total of eight factors; 95%CI = 5.1 to 19.6).

Unpublished local prospective cross-sectional data obtained between November 2009 and June 2010 has demonstrated differences between 226 FU and 173 infrequent users. FU were more often younger with a mean age of 51 vs. 56, and had five to eighteen admissions in the previous 12 months. They cumulated vulnerabilities in terms of somatic problems, mental diseases, risk behavioral indicators and unfavorable social determinants of health[64]. These vulnerabilities were also more often present among the local hyper-frequent (12 attendances or more during the past year) user population[65].

ED-FU require specific interventions

Interventions vary, according to a recent systematic review of the literature by our research team that identified different types of interventions aimed at improving the management of adult ED FU[66] and at assessing their effectiveness. Most of the studies describe interventions referring to and/or inspired by CM [28 31 47 51-53 67].

Conclusions based on research in the field, and knowledge gaps

ED serves a large base of vulnerable patients. Compared to infrequent or non-users, most of the ED-FU visitors are identified as vulnerable patients because they are more likely to be of low socio-economic status, be more isolated and live alone. They report more chronic medical conditions, have a higher mortality rate and consume more healthcare resources.

Establishing a model of care for those patients locally could improve patient health, and decrease their use of the healthcare system and reduce consequent costs.

In the literature, interventions aimed at improving the management of ED-FU have demonstrated several positive outcomes, but there are still some **knowledge gaps**:

- There is only one Randomized Controlled Trial (RCT) showing a significant reduction in ED use by FU compared to usual care patients[51];
- The threshold for number of visits varies across the three existing RCT[44 51 52]; only one is based on the definition of five or more attendances per year, corresponding to more than known random events[51];
- Cost reductions were demonstrated in three studies[31 51 53], but only one is a RCT[51], and the other two did not contain any control groups; and
- Patient baseline characteristics and healthcare specificities shown in 11 studies included in a systematic review by Althaus and al.[66] were only relevant within the country in which each study was conducted (USA, Sweden, Canada, Australia, and UK).

b) Hypothesis

Since June 2009, a project at the University Hospital of Lausanne devoted to improving healthcare for vulnerable populations at the clinical level has been created and financed at the direction of the University Hospital. ED-FU have been identified and studied locally retrospectively, prospectively, and in a case series design as proxies for vulnerable populations [35 64 65]. Specific interventions have been identified and studied in a systematic review of the literature[66]. The trial proposed herein would take the next logical step towards establishing a specific intervention for a specific vulnerable population, and estimating its clinical appropriateness, cost-benefit ratio and efficiency.

The proposed project tests the hypotheses that CM intervention as compared with standard emergency care

- is a more efficient use of healthcare resources and reduces ED attendance,

- is cost-saving and
- improves quality of life,
- altogether leading to favorable cost-utility ratio.

c) Aim of the study

Because of the knowledge gaps mentioned in page 5 in a key topic for patients, clinicians, and policymakers, we would like to demonstrate in this trial that an intervention on ED-FU by a multidisciplinary mobile team (based on CM care patterns) will show emergency department use reductions, is a more appropriate way to improve quality of life than is standard emergency care by nurses and physicians, will be cost-saving and is a more efficient use of healthcare.

d) Outcomes

Primary outcome

The primary outcome will be the number of ED visits made by FU in the intervention and the control group.

Secondary outcomes

The secondary outcome measure will be a delineation of the total costs of healthcare resource use incurred by ED-FU in the perspective of the healthcare provider.

The tertiary outcome will be an assessment of the health status of participants, as measured by the EQ-5D.

Each outcome will be discussed in details in the part 5e), p.10-11

e) Rational

This study is coordinated with recent local research projects dedicated to assessing profiles and improving healthcare for ED-FU, who are considered to be a highly vulnerable subgroup and a proxy for vulnerable populations in general.

Taking care of a growing number of vulnerable patients requires specific interventions. However, patterns of care that have succeeded elsewhere have to be tested in local or national settings before being introduced into a new context of care among local patients. A mixed methodology using quantitative, qualitative and medico-economic analysis is needed.

Because of some knowledge gaps in the literature, we would like to demonstrate in this trial that a case management intervention is a more efficient use of healthcare and will show emergency department use reduction, will be more cost-saving, and will be a more appropriate means of improving quality of life, than is standard emergency care alone by nurses and physicians serving ED-FU.

Positive findings would constitute a strong incentive to replicate these studies on a larger scale, in a multicenter study with more extensive follow-up procedures. Positive findings would also suggest that specific populations need specific care, and would have major implications for healthcare quality and costs.

Finally, the total number of ED visits in Switzerland is around 1.3 million per year (O. Hugli, unpublished data) and has been steadily growing. If our intervention results in a reduction in the number of ED visits, the impact at the national level could be significant.

5. Plan général

a) **Study design**
b) **Setting**
c) **Participants**
d) **Interventions**
e) **Outcomes**
f) **Randomization, allocation**

a) Study design

This study is a RCT that compares comprehensive CM care associated to standard emergency care with standard emergency care alone among ED-FU vis-à-vis efficiency, cost-utility and appropriateness for improving quality of life (**Figure 1**).

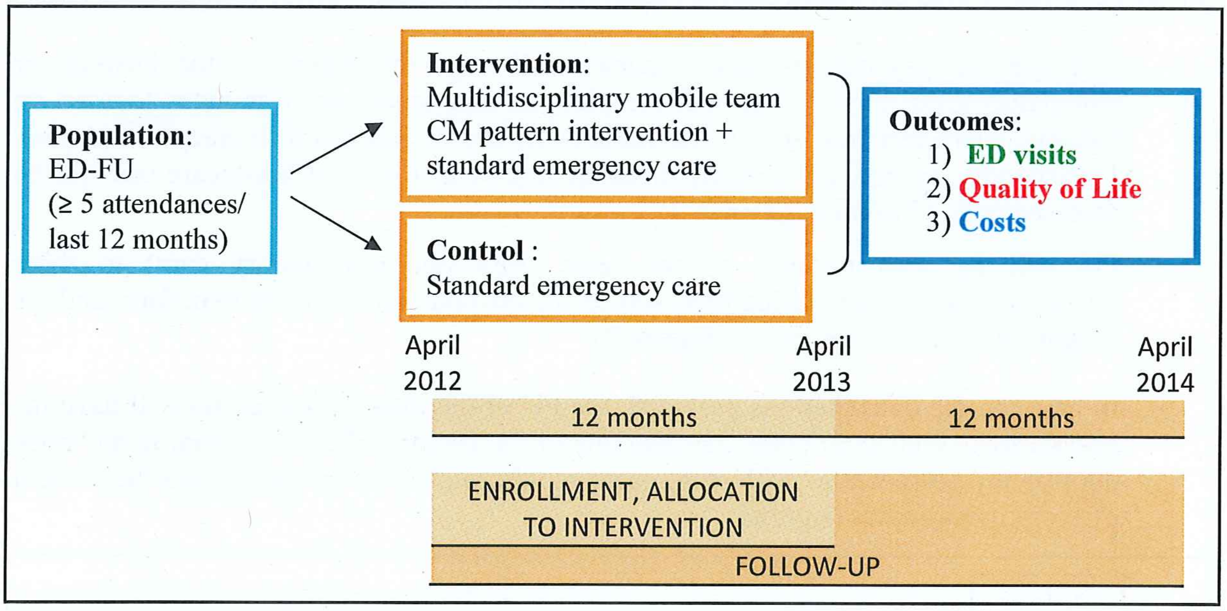

Figure 1: Study design with inclusion and follow-up time table.

b) Setting

The study will be conducted in the Lausanne University Hospital ED. This facility is an urban public hospital serving (with other non-university hospitals) 770,000 people that provides medical, surgical, and mental health care for 35,000 annual ED visits, and is one of the five teaching hospitals located in Switzerland. It will also be realized in the Department of Ambulatory Care and Community Medicine ED who provides 15'000 annual ED visits.

The study will be conducted from April 2012 to April 2015. We have planned to make a follow-up during 12 months; nevertheless this follow-up could be extended to 18 months if the intermediate analysis shows us that more time is needed to demonstrate a clear difference in the outcomes between the intervention and the control groups.

c) Participants

Frequent user identification: an automated 24-hour, seven days per week detection system based on the ED patient tracking software will identify all patients who have attended the hospital and ambulatory EDs five times or more during the previous 12 months. If the patients are no longer in the ED, a member of the CM team will make three attempts to contact them by telephone within 24 to 48 hours of their departure from the hospital, and try to obtain their

oral consent to be interviewed by telephone (all the details concerning Participants are described in the part 6.c, page 15).

d) Interventions

d.1) The multidisciplinary mobile team CM pattern intervention

The mobile team consists of a psychologist and a nurse-practitioner working together with each patient. A medical supervisor (i.e. general practitioner) stages the implementation of the project, monitors the team consolidation process, and is available for medical consultations for any difficult medical conditions in patients. He has the responsibility of verifying that the intervention offered is the preferred one.

Patients randomized to case management will receive an intervention designed to offer support for ED-FU and the professionals who work with them inside the hospital, as well as for the community medical and social providers who will maintain outside continuity of care:

- CM team (a psychologist and a nurse) will meet the patient at the hospital or ambulatory EDs. Firstly, they will complete an assessment of 30 minutes focused on baseline characteristics, social determinants of health and health literacy[68-70] (**Annex 1**). Secondly, mental and somatic diseases, risk behaviors, and healthcare use will be assessed over 45 minutes (**Annex 2**).
- FU will be seen initially by the team from one (ambulatory care) to three (hospitalization) times during their stay at the hospital, and again at two, four, and six months later at their residence (**Figure 2**)
- In between the consultations provided, the FU of the intervention group will have the possibility to contact at every moment one of the persons of the CM team in an "open door policy perspective" with subsequent monitoring of the frequency and the content of every intervention required.

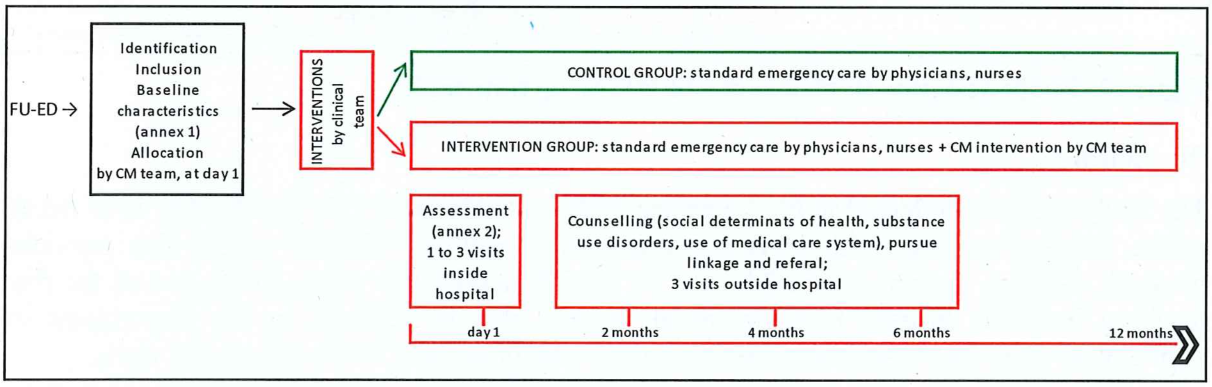

Figure 2: Timetable for every ED-FU included in the study with interventions (at day 1, 2 months, 4 and 6 months for the intervention group).

- Initial (day 1) and follow-up interventions by CM team (at 2, 4, 6 and 12 months) will include counseling about social determinants of health, substance-use disorders, and the use of medical care systems. Counseling will be based on motivational interviewing (empathy, collaboration, autonomy and valorization), while avoiding confrontation. Each member of the CM team will have a check-list about the proposals and advices that they have to give to every FU patients and about the material (flyers, addresses, etc) that they have to provide.

- The primary goals of the interventions are to furnish specific assistance and to provide referrals for the patients:

  - If the social determinants of the ED-FU are not adequate, the team will

    → Lend assistance for obtaining income entitlements, health insurance coverage if eligible, stable housing (e.g. shelters for the homeless), schooling for children, prevention of potential violence (i.e. conjugal and/ or against the children) in the home.

  - If there are mental disturbances, the team will

    → Refer to mental health departments inside the hospital, and if necessary, to a psychiatrist, psychologist or general practitioner (GP) out in the community.

  - If the patient presents risk behaviors (alcohol consumption, smoking, and other drug use), the team will

    → Refer to substance abuse services and links to community services in order to maintain continuity of care.

  - In case of somatic problems (and the patient either has no GP or has not consulted with them for a long time) the team will

    → Find a new GP or make contact with the previous provider, contingent on the patient's consent.

- Each member of the team will follow a maximum caseload of 20 patients as a case-manager. We will take into account the CM team capacity to allow consistent recruitment over the time: if the program reach capacity, particularly when the intervention group of participants is enrolled, making it necessary to stop recruitment until clinical capacity is again available.

- The linkage to medical and social services providers inside the hospital (with the participation of the CM team in network meeting crisis interventions organized by the professionals involved in each case) will continue outside the hospital with GP, home visits by nurses and social services. The CM team will centralize documents and facilitate communications between all care providers, assuring an assertive, ongoing community outreach in order to maintain continuity of care (**Annex 3**).

This program uses an assertive clinical case management model that is patient-identified, patient-directed and developed to provide high intensity services. It provides a continuum of hospital- and community-based patient services that include clinical assessment, outreach referral, coordination and communication with other service providers. Additional components are patient education in a motivational perspective, individual counseling, crisis intervention, medical assessment and on-going medical care.

Teamwork, case conferences, continuing education

The core team (nurse practitioners, psychologist and general practitioner) is supported by several vulnerable population experts within various hospital departments, such as Gynaecology-obstetrics, Paediatrics, Psychiatry, and Ethics, who act as contact persons for their department and complement the team interventions with their expertise on specific problems of gender, minor children, mental diseases and ethical concerns.

Members of the CM team will receive an intensive training in motivational interviewing and cross-cultural competences, take specific classes in adequate referral to social assistance (e.g. income entitlements and stable housing), alcohol, drug use disorders and home violence.

Because of the potential difficult situations concerning many of those vulnerable patients, the members of the CM team will benefit of a psychological support.

d.2) The control intervention

Patients randomized to control group (usual care) will receive standard emergency care by physicians (resident or attending physician) and nurses, without the case manager been involved. Nevertheless, the mobile team will take contact with each patient of the control group, giving them short information through a flyer **(flyer)** which will underline the existence of the mobile team, its addresses and telephone numbers.

The assessment effect will be present in the control group as in the intervention group.

Concerning usual care, after the first orientation by a nurse, when the intervention is provided by a resident and he/she will be systematically supervised by a chief resident. Referrals to other specialties are routinely done by residents acting as liaisons to the appropriate hospitalization sector; there is no systematic presence or involvement of psychologist or nurse practitioner.

Finally, patients randomized to usual care will be eligible to receive CM services at the end of the study. In any critical situation where the patient included in the study (in the intervention or the control group) will need a hospitalization, this will be done.

e) Outcomes

e.1) Primary outcome: number of ED visits[28 31 43 46 48 51 53]

The primary outcome will be the number of ED visits made by FU. This information will be available through the Lausanne hospital/ambulatory electronic record system and hospital/ambulatory administrative databases covering a period of 12 months after the initial emergency department visit.

Diaries will be also generated from the beginning of the study. Patients will be asked to report all visits (ED visits to the CHU or to another hospital and all outpatient visits) in a diary during the 12 (or 18 months) following their first visit (**Figure 3**). On the one hand, patients will be contacted by telephone by the nurse researcher to answer questions about their use of the healthcare system and to verify that they have completed their diaries appropriately. If this is not the case, the nurse researcher will help the patient find ways and strategies for improving their reporting. If necessary, incentives would be used to help FU patients completing diaries appropriately. The quality control will be repeated at 3, 6, 9 months and (final) 12-month follow-ups. On the other hand, confirmatory telephone calls to other hospitals, medical institutions or private offices that the patient claims to have visited will be made by the nurse researcher, after obtaining the patient's written permission to do so. Moreover, validity of FU patients' answers could be assessed by matching their answers to our gold standard electronic record system of visits within the CHUV. Note however, that this will only be possible for health care (HC) services provided by the CHUV.

The timetable for every ED-FU included in the study with intervention and outcomes assessment is reported in **figure 3.**

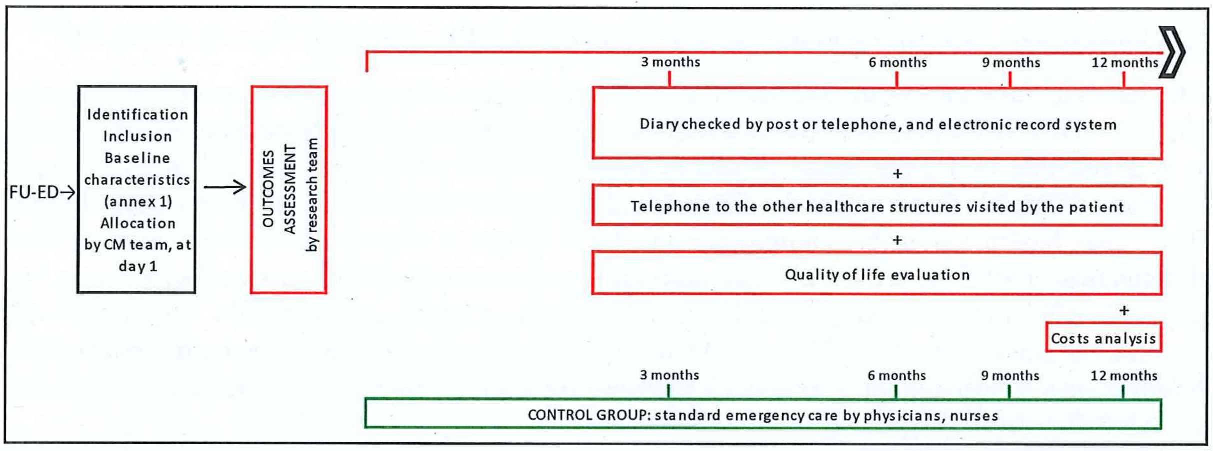

Figure 3: Timetable for every ED-FU included in the study with outcomes assessment (at 3, 6, 9 and 12 months for the intervention and the control group).

e.2) Secondary outcome: costs analysis[31 51 53 71]

The second outcome measure focuses on the costs induced by the health care resources used by the FU patients. Their healthcare consumption is related to services provided by the CHUV but we cannot exclude that the FU also use services provided by other hospitals/institutions in the community (services provided outside the CHUV).

1) Concerning services provided by the CHUV, different hospital administrative databases which record all inpatient and outpatient admissions will allow us to have access to all health care used by the FU and consequently the related costs. These latter's will be composed of costs related to:

   a. Outpatient resources induced by ED attendances,

   b. Inpatient resources induced by ED attendances,

   c. Not ED related outpatient resources used within the hospital,

   d. The ED case management multidisciplinary team intervention.

The access to the "système de gestion administrative du CHUV" that encompasses accounting analytical systems, as well as outpatient invoicing department, will allow the collection of required information in order to calculate costs.

2) Concerning services used outside the CHUV, information recorded in diaries will help identifying in what extent FU seek and use services outside from the CHUV perimeter, for instance whether patients use ED of another hospital etc... The diary, by recording the date and the place of all visits the FU would have had, will also help identifying the type of services (health or/and social services) used by FU.

   The CM intervention may affect how the FU use the HC system in general. The primary outcome of the project will allow us to identify whether the CM intervention is associated with a decrease in the number of visits at the CHUV ED. However, it is also important to investigate in what extent the potential decrease in HC resources used at the CHUV ED is (not) associated with an increase of HC resources used outside the CHUV ED. Consequently, information of the Diary will help capturing a substitution effect between the HC utilization at the CHUV ED and the HC utilization external to the CHUV. Based on average unit costs, costs associated with the Diary HC consumption will be simulated.

e.3) Secondary outcome: standardized measure of health status via EuroQol (EQ-5D)[72]

The last outcome will be an assessment of the health status of participants, as measured by the EQ-5D. This instrument is a non-disease specific, self-report of health-related quality of life. It is applicable to a wide range of health conditions and treatments, and provides a simple measure of health that is used in clinical and economic analysis. Each respondent defines their own health status by combining one level (from a choice of 3) from each of five dimensions: mobility; self-care; usual activities; pain/discomfort; and anxiety/depression. For any state of health reported, an EQ-5D score reflecting a health utility weight will be derived. The health status outcome will be collected by the nurse researcher at four time points (i.e. baseline and follow-ups at 3, 6 and 12 months) for each patient in both the intervention and the control group[73] (**Figure 3**).

To complement the assessment of the health status of participants, we will address the patient satisfaction trough 5 items questionnaire (in process of validation through the health Swiss survey).

f) Randomization, allocation

f.1) Sequence generation

The randomization list associating questionnaire numbers to intervention or control groups will be generated by the statistician using block randomization prior to the start of the study. Computer-based, randomly-generated permuted blocks of random size will assure group size balance (www.randomization.com). Patients will then be allocated to either a group A or a group B. The institution's pharmacy will then decide if group A or B is to be the intervention group, therefore blinding the statistician to the true allocation. The randomization list will be held by the institution's pharmacy during the opening hours. At night and during the week-end, the case-management team will be informed of the FU-ED consultations through email by the ED staff. They will take contact with every FU the day after or the following Monday, with the intervention of the institution's pharmacy for the randomization.

f.2) Allocation concealment mechanism

The institution's pharmacy will hold the randomization list and reveal the patient's allocation corresponding to the questionnaire number. The allocation will be reported by phone once baseline characteristics (**Annex 1**) have been collected by the CM team. The patient will then be informed about the procedures he should follow, without knowing whether he has been assigned to the intervention or to the control group.

f.3) Blinding

The research nurse, responsible of collecting outcomes, will be blinded to the patient's allocation (when collecting baseline characteristics, the CM Team will be also blinded to allocation). The statistician will be blinded to the true group until the analyses are done. As the intervention is also provided by the ED staff that will interact with the case-management team for the intervention group patients, it is impossible to have them blinded. Patients will agree to take part in a study where they will be managed by a coordinated team. Blinding effectiveness will be assessed by asking patients at the end of their follow-up period if they thought they were in the intervention or the control group. Since it delivers the intervention, CM team cannot be blinded. The data collecting manager, also responsible of quality control, will have access to all data and therefore cannot be blinded.

f.4) Statistical methods

Groups will be compared from their initial allocation independently of eventual crossover (intention to treat analyses). The principle measure of effect is an individual's average

reduction of visits to ED over 12 months compared to the number of visits observed in the control group. This will be calculated using linear regression with number of visits during 12 months follow-up as an independent variable, and group allocation and yearly number of ED visits prior to intervention as dependent variables. In case of group imbalance, secondary analysis will test their confounding effects by measuring effect after adjusting for these confounding effects in the linear regression. Known determinants of FU-ED are to be considered as potential confounders if, by chance due to the randomization, we are to observe a relative difference of 20% between groups.

Frequent users are known to visit ED on regular bases on a short period of time (regression to the mean), so we also expect to see the decrease of the number of visits in the control group. Our analysis will measure the true effects of the intervention taking this phenomenon into consideration.

In terms of medico-economic analysis, benefits of the care management program will be evaluated by health gains expressed in Quality Adjusted Life Years (QALYs) over the 24-month period. A cost-utility analysis from the healthcare provider perspective will be conducted by combining the use of two outcomes (i.e. the costs and health status in terms of quality of life). A cost-utility ratio will then be calculated. A sensibility analysis will also be conducted in order to estimate the confidence interval for the cost-utility ratio. Uncertainty will be assessed by univariate and probabilistic sensitivity analysis (Monte Carlo simulation).

6. Sélection des sujets

a) Flow diagram
b) Sample size
c) Participants

a) Flow diagram

The flow diagram (**Figure 4**) shows the progression through the phases of the RCT of intervention based on multidisciplinary mobile team case management pattern, parallel to standard emergency care for ED-FU. The numbers listed on the diagram are the results of a recent cross-sectional study conducted in the same setting at the Lausanne University Hospital ED[64].

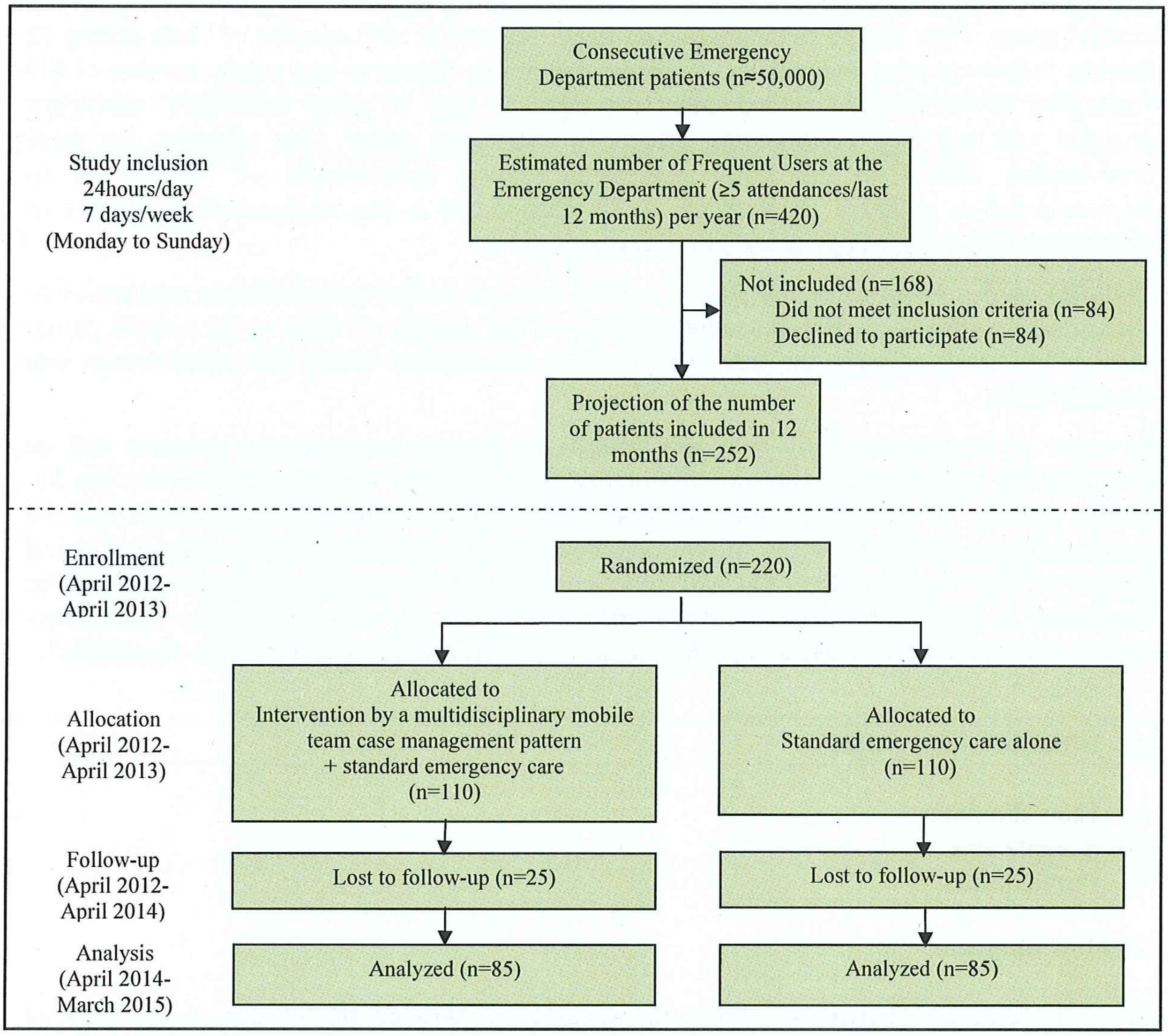

Figure 4: Flow diagram including estimates of number of patients to be included based on prior studies on ED-FU conducted by our research group.[35 64 74]

b) Sample size

The sample size has been calculated to detect a between-group average reduction of two visits per year to the ED (i.e. four visits for the intervention group versus two for the control, with an expected standard deviation of four in both groups), in accordance with the results of a systematic review of the literature by Althaus and al.[66] With a significance level of 0.05 and power of 0.9, each group should include at least 85 participants. From previous observations of the CM team clinical activity, we expect 30% of patients to be refugees or undocumented migrants. The expected proportion of lost to follow-up is higher for refugees/undocumented migrants (20%) than for the rest of the population (10%). We therefore voluntary overestimated the dropout rate for the overall population to be of 20%. The total required simple size has been rounded up to 220 patients (110 in each group). The intermediate analysis is planned once the first 80 patients have been followed-up to 12 months

c) Participants

Frequent user identification: an automated 24-hour, seven days per week detection system based on the ED patient tracking software will identify all patients who have attended the ED five times and more during the previous 12 months. A member of the CM team will approach

the FU; the FU will receive written information, an oral explanation and sufficient time to think about the possibility to integrate the study. If he agrees, he will receive an informal written consent.

If the patients are no longer in the ED, a member of the CM team will make three attempts to contact them by telephone within 24 to 48 hours of their departure from the hospital, and try to obtain their oral consent to be interviewed by telephone.

The general practitioner of each patient included in the study will be alerted by telephone, email or mail by the team member in charge of the patient.

Inclusion criteria: patients presenting at ED between the 1st April 2012 and the 30th April 2013, will be eligible for study participation, provided they are at least age 18, have made five or more visits to the ED in the previous 12 months and are capable of communicating in any of the languages spoken by the team (i.e. French, German, Italian, English and Spanish) or through a community interpreter.

Exclusion criteria: patients will not be enrolled if they cannot give informed consent or are ineligible to receive CM services (e.g. acutely confused, acutely psychotic, with dementia or intoxicated), are not expected to survive or will not remain in Switzerland for 12 to 18 months after enrollment; prisoners will not be included.

7. Déroulement de l'étude et investigations prévues

All the information concerning the setting, the periods of time related with enrollment, allocation to intervention and follow-up and, the different interventions are presented in the part 5. Figure 1, 2, 3 give more precisions concerning those information.

8. Surveillance médicale

Refer to part 5, pages 7 to 13. Criteria exclusion are explained in point 6. c) page 15.

9. Rôle du personnel infirmier

There will be no extra-work for the ED staff, including physicians and nurses.

10.Médicaments

Not applicable

11.Evaluation des risques

During and following the participation of all subjects (in either of the groups), the research staff will ensure that adequate medical care is provided to subjects who experience any adverse events. Adverse effects could be noted by FU in their diaries, and discussed with the nurse researcher at 3, 6, 9 and 12 months or any time by telephone with the primary investigator. Considering that the intervention has a low risk of causing harm to participants, no stopping rules were planned.

12.Couverture d'assurance (RC)

Not applicable

13.Formulaires d'information et de consentement

Refer to **Annex 4** and **5**

14.Traitement de données personnelles et d'échantillons biologiques

All information obtained will be kept anonymous and secure by the staff investigators during all the period of the investigation and after the end of the investigation, during a period of ten years.

15.Plan de financement et rétribution :

The entire budget for the clinical team activity comes from the grant "Axe stratégique populations vulnerable" Direction of the Centre Hospitalier Universitaire Vaudois in the perspective of the CHUV strategic plan (2009-2013). The amount dedicated for this clinical team during the year 2012-2013 will be about CHF400'000 per year. The entire amount for the research team activity comes from a national grant (FNS 30023B_135762/1) for a total amount of 422'149 (see the budget bellow).

Répartition du subside par rubrique

	Total	1ère tranche	2ème tranche	3ème tranche
**Projet**				
Matériel, entretien	4'798	4'798	0	0
Déplacements	16'390	2'880	5'940	7'570
Divers	3'000	0	0	3'000
Salaires	343'070	87'388	137'903	117'779
Charges sociales	54'891	13'982	22'064	18'845
**Total**	422'149	109'048	165'907	147'194

Début: 1er avril 2011 Durée: 36 mois

Conditions

Salaires:

Accordés:
N.N., Postdoc, 36 mois 50%
CHF 27'127.- / 49'306.- / 39'548.-
N.N., Postdoc, 36 mois 80%
CHF 40'133.- / 70'864.- / 54'742.-
N.N., Postdoc, 36 mois 20%
CHF 15'017.- / 10'918.- / 18'378.-
N.N., Assistant, 36 mois 20%
CHF 5'111.- / 6'815.- / 5'111.-

Charges sociales: 16% = CHF 54'891.-

Figure 5: national grant for the research team activity (FNS 30023B_135762/1)

Finally, there will be no retribution amount of money for each patient (for the intervention or the control group) who will benefit of the intervention or the presentation of the case-management team.

16.Etude impliquant la participation de praticiens installés

Not applicable

17. Informations au personnel soignant médical et paramédical

This information will figure in the two emergency department rooms of the Centre Hospitalier Universitaire Vaudois and Policlinique Médicale Universitaire. The content of this information is bellow. Different oral presentations will be done before the beginning of the study on April 2012.

Mesdames, Messieurs, Chers Collègues,

Les patients usagers fréquents des services d'urgence sont souvent considérés comme vulnérables de par leur mortalité, morbidité, mais aussi de par leurs caractéristiques et demandes sociales. Le recours fréquent aux services d'urgences n'est souvent pas adapté, les services d'urgence ne répondant pas aux demandes spécifiques socio-médicales de ce type de patients.

Il a été démontré dans quelques études internationales qu'une prise en charge en deuxième ligne par de petites équipes interdisciplinaires avec une orientation de type « case-management », s'adapte de manière plus adéquate aux demandes spécifiques de ce type de patients, avec un impact sur la qualité de prise en charge, la fonctionnalité du service d'urgences et le coût des soins.

Une équipe interdisciplinaire (Equipe mobile vulnérabilitéS, EmvS) a été créée en juillet 2010 au sein de la Cité hospitalière universitaire et travaille depuis lors avec ce type de patients notamment.

Dans le cadre d'une étude du fonds national (FNS 30023B_135762/1) qui débutera le 1er avril 2012, nous étudierons l'impact de l'existence de cette équipe auprès du service des urgences du CHUV et du secteur des urgences ambulatoires de la Policlinique. Après intervention de cette équipe en deuxième ligne, nous souhaitons démontrer dans le cadre de cet essai clinique randomisé, que l'intervention auprès d'usagers fréquents est la façon la plus appropriée de diminuer le recours aux urgences, d'améliorer la qualité de vie de ces patients et à terme de diminuer les coûts institutionnels et sociétaux y relatifs.

Il n'y aura aucun risque potentiel pour chacun des patients inclus dans l'étude, et aucun surplus de travail vous concernant. Vous n'aurez par ailleurs pas à changer vos habitudes de travail avec l'EmvS avec qui vous travaillez déjà. Enfin une présentation orale de cette étude sera réalisée dans les différents secteurs des urgences du CHUV et de la PMU.

18.Reference list

1. Lantz PM, House JS, Lepkowski JM, Williams DR, Mero RP, Chen J. Socioeconomic factors, health behaviors, and mortality: results from a nationally representative prospective study of US adults. *JAMA* 1998;279(21):1703-8.
2. Marmot M, Friel S, Bell R, Houweling TA, Taylor S. Closing the gap in a generation: health equity through action on the social determinants of health. *Lancet* 2008;372(9650):1661-9.
3. Marmot MG. Status syndrome: a challenge to medicine. *JAMA* 2006;295(11):1304-7.
4. Wilkinson RG. Socioeconomic determinants of health. Health inequalities: relative or absolute material standards? *BMJ* 1997;314(7080):591-5.
5. OFS. http://www.bfs.admin.ch/bfs/portal/fr/index.html. Neuchâtel: Office féderal de la statistique, 2009.
6. Longchamp C, Aebersold M, Rousselot B, Ratelband-Pally S. Sans-papiers en Suisse: c'est le marché de l'emploi qui est déterminant, non pas la politique d'asile. [Rapport final sur mandat de l'Office fédéral des migrations]. Berne: OFM, 2005.
7. Piguet E, Losa S. *Travailleurs de l'ombre? Demande de main-d'oeuvre du domaine de l'asile et ampleur de l'emploi d'étrangers non déclarés en Suisse.* Seismo ed. Zurich, 2002.
8. Muennig P, Fiscella K, Tancredi D, Franks P. The Relative Health Burden of Selected Social and Behavioral Risk Factors in the United States: Implications for Policy. *Am J Public Health.*
9. Rew L. Health risks of homeless adolescents. Implications for holistic nursing. *J Holist Nurs* 1996;14(4):348-59.
10. ISPA. http:www.sfa-ispa.ch/. Lausanne: Institut suisse de prévention de l'alcoolisme et autres toxicomanies 2009.
11. Derose KP, Escarce JJ, Lurie N. Immigrants and health care: sources of vulnerability. *Health Aff (Millwood)* 2007;26(5):1258-68.
12. Fiscella K, Franks P, Gold MR, Clancy CM. Inequality in quality: addressing socioeconomic, racial, and ethnic disparities in health care. *JAMA* 2000;283(19):2579-84.
13. Shi L, Stevens GD. Vulnerability and unmet health care needs. The influence of multiple risk factors. *J Gen Intern Med* 2005;20(2):148-54.
14. Aday LA. Health status of vulnerable populations. *Annu Rev Public Health* 1994;15:487-509.
15. Flaskerud JH, Winslow BJ. Conceptualizing vulnerable populations health-related research. *Nurs Res* 1998;47(2):69-78.
16. Gelberg L, Andersen RM, Leake BD. The Behavioral Model for Vulnerable Populations: application to medical care use and outcomes for homeless people. *Health Serv Res* 2000;34(6):1273-302.
17. Mechanic D, Tanner J. Vulnerable people, groups, and populations: societal view. *Health Aff (Millwood)* 2007;26(5):1220-30.
18. Rogers AC. Vulnerability, health and health care. *J Adv Nurs* 1997;26(1):65-72.
19. Vladeck BC. How useful is 'vulnerable' as a concept? *Health Aff (Millwood)* 2007;26(5):1231-4.
20. Moore LW, Miller M. Initiating research with doubly vulnerable populations. *J Adv Nurs* 1999;30(5):1034-40.
21. Moore G, Gerdtz M, Manias E, Hepworth G, Dent A. Socio-demographic and clinical characteristics of re-presentation to an Australian inner-city emergency department: implications for service delivery. *BMC Public Health* 2007;7:320.

22. Locker TE, Baston S, Mason SM, Nicholl J. Defining frequent use of an urban emergency department. *Emerg Med J* 2007;24(6):398-401.
23. Dent AW, Phillips GA, Chenhall AJ, McGregor LR. The heaviest repeat users of an inner city emergency department are not general practice patients. *Emerg Med (Fremantle)* 2003;15(4):322-9.
24. Fuda KK, Immekus R. Frequent users of Massachusetts emergency departments: a statewide analysis. *Ann Emerg Med* 2006;48(1):9-16.
25. Hansagi H, Allebeck P, Edhag O, Magnusson G. Frequency of emergency department attendances as a predictor of mortality: nine-year follow-up of a population-based cohort. *J Public Health Med* 1990;12(1):39-44.
26. Huang JA, Tsai WC, Chen YC, Hu WH, Yang DY. Factors associated with frequent use of emergency services in a medical center. *J Formos Med Assoc* 2003;102(4):222-8.
27. Byrne M, Murphy AW, Plunkett PK, McGee HM, Murray A, Bury G. Frequent attenders to an emergency department: a study of primary health care use, medical profile, and psychosocial characteristics. *Ann Emerg Med* 2003;41(3):309-18.
28. Skinner J, Carter L, Haxton C. Case management of patients who frequently present to a Scottish emergency department. *Emerg Med J* 2009;26(2):103-5.
29. Malone RE. Heavy users of emergency services: social construction of a policy problem. *Soc Sci Med* 1995;40(4):469-77.
30. Lucas RH, Sanford SM. An analysis of frequent users of emergency care at an urban university hospital. *Ann Emerg Med* 1998;32(5):563-8.
31. Okin RL, Boccellari A, Azocar F, Shumway M, O'Brien K, Gelb A, et al. The effects of clinical case management on hospital service use among ED frequent users. *Am J Emerg Med* 2000;18(5):603-8.
32. Sun BC, Burstin HR, Brennan TA. Predictors and outcomes of frequent emergency department users. *Acad Emerg Med* 2003;10(4):320-8.
33. Ullman R, Block JA, Stratmann WC. An emergency room's patients: their characteristics and utilization of hospital services. *Med Care* 1975;13(12):1011-20.
34. Hansagi H, Allebeck P, Edhag O. Health care utilization after referral from a hospital emergency department. *Scand J Soc Med* 1989;17(4):291-9.
35. Bieler G, Paroz S, Faouzi M, Trueb L, Vaucher P, Althaus F, et al. Social and Medical Vulnerability Factors of Emergency Department Frequent Users in a Universal Health Insurance System. *Academic emergency medicine : official journal of the Society for Academic Emergency Medicine* 2012.
36. Hsia RY, Tabas JA. Emergency care: the increasing weight of increasing waits. *Arch Intern Med* 2009;169(20):1836-8.
37. LaCalle E, Rabin E. Frequent users of emergency departments: the myths, the data, and the policy implications. *Ann Emerg Med*;56(1):42-8.
38. Kne T, Young R, Spillane L. Frequent ED users: patterns of use over time. *Am J Emerg Med* 1998;16(7):648-52.
39. Oktay C, Cete Y, Eray O, Pekdemir M, Gunerli A. Appropriateness of emergency department visits in a Turkish university hospital. *Croat Med J* 2003;44(5):585-91.
40. Ovens HJ, Chan BT. Heavy users of emergency services: a population-based review. *CMAJ* 2001;165(8):1049-50.
41. Care plans for frequent flyers save money, cut costs: hospital saves more than $500,000. *Hospital Case Management* 2006;14(2):22.
42. Reducing 'frequent flyers' in the emergency room: Medicaid members connect with primary care. *Patient Education Management* 2008;15(6):67-68.
43. Andren KG, Rosenqvist U. Heavy users of an emergency department: psycho-social and medical characteristics, other health care contacts and the effect of a hospital social worker intervention. *Soc Sci Med* 1985;21(7):761-70.

44. Hansagi H, Olsson M, Hussain A, Ohlen G. Is information sharing between the emergency department and primary care useful to the care of frequent emergency department users? *Eur J Emerg Med* 2008;15(1):34-9.
45. Lee KH, Davenport L. Can case management interventions reduce the number of emergency department visits by frequent users? *Health Care Manag (Frederick)* 2006;25(2):155-9.
46. Olsson M, Hansagi H, Tomson Y, Sjoberg S. [Individualised care plans - a feasible way to improve care for frequent users of the emergency department?]. *Socialmed Tidskr* 2004;1(2):122-29.
47. Phillips GA, Brophy DS, Weiland TJ, Chenhall AJ, Dent AW. The effect of multidisciplinary case management on selected outcomes for frequent attenders at an emergency department. *Med J Aust* 2006;184(12):602-6.
48. Pope D, Fernandes CM, Bouthillette F, Etherington J. Frequent users of the emergency department: a program to improve care and reduce visits. *CMAJ* 2000;162(7):1017-20.
49. Powell SK, Tahan HA. Case management. A practical guide for education and practice. Third edition. ed. Philadelphia: Wolters Kluwer | Lippincott Williams & Wilkins, 2010.
50. Sargent P, Pickard S, Shaeff R, Boaden R. Patient and carer perceptions of case managmeent for long-term conditions. *Helath and Social Care in the Community* 2007;15(6):511 - 519.
51. Shumway M, Boccellari A, O'Brien K, Okin RL. Cost-effectiveness of clinical case management for ED frequent users: results of a randomized trial. *Am J Emerg Med* 2008;26(2):155-64.
52. Spillane LL, Lumb EW, Cobaugh DJ, Wilcox SR, Clark JS, Schneider SM. Frequent users of the emergency department: can we intervene? *Acad Emerg Med* 1997;4(6):574-80.
53. Wassmer RW, Winward L, Derlet R. Does Counseling Reduce Frequent Emergency Department Use?, 2008.
54. Brewer BB, Jackson L. A case management model for the emergency department. *J Emerg Nurs* 1997;23(6):618-621.
55. Bristow DP, Herrick CA. Emergency department case management: the dyad team of nurse case manager and social worker improve discharge planning and patient and staff satisfaction while decreasing inappropriate admissions and costs: a literature review. *Lippincotts Case Manag* 2002;7(6):243-51.
56. CMSA. Case management model act of 2009: CMSA, 2009.
57. Schneidermann M, Ferandez A. Case management / multidisciplinary care models. In: King T, Wheeler M, editors. *Medical management of vulnerable and underserved patients*. New York: McGraw-Hill, 2007:151 - 158.
58. Walsh KT, Zander K. Emergency department case management. Marblehead: HCPro, Inc., 2007.
59. Wetta-Hall R, Berry M, Ablah E, Gillispie JM, Stepp-Cornelius LK. Community case management: a strategy to improve access to medical care in uninsured populations. *Care Manag J* 2004;5(2):87-93.
60. Berger CS. Social work case management in medical settings. In: Roberts AR, Gilbert JG, editors. *Social workers' desk reference.* Oxford: University Press, 2002:497-501.
61. Carr D. Implications for case management. Ensuring access and delivery of quality health care to undocumented immigrant populations. *Lippincott's Case Management.* 2006;11(4):195-206.
62. Netzwerk CM Schweiz. Definition Case Management. Standards Case Management, 2006.

63. Walcher M. Das Konzept Case Management und seine Anwendung. *Education Permanente* 2009(4):12-13.
64. Bodenmann P, Stucki S, Paroz S, Hugli O, Ansermet C, Diserens EA, et al. Frequent users of the Emergency Department as identification criteria of social and medical vulnerability: a descriptive cross- sectional study. *In process* 2011.
65. Bodenmann P, Stucki S, Guyot S, Trueb L, Daeppen JB. Taking care of the most vulnerable: a case- series of patients with 12 visits or more at the ED in 2009. *In process* 2011.
66. Althaus F, Paroz S, Hugli O, Ghali WA, Daeppen JB, Peytremann-Bridevaux I, et al. Effectiveness of interventions targeting frequent users of emergency departments: a systematic review. *Annals of emergency medicine* 2011;58(1):41-52 e42.
67. Lee A, Lau FL, Hazlett CB, Kam CW, Wong P, Wong TW, et al. Factors associated with non-urgent utilization of Accident and Emergency services: a case-control study in Hong Kong. *Soc Sci Med* 2000;51(7):1075-85.
68. Berkman ND, Sheridan SL, Donahue KE, Halpern DJ, Crotty K. Low health literacy and health outcomes: an updated systematic review. *Ann Intern Med* 2011;155(2):97-107.
69. Chew LD, Griffin JM, Partin MR, Noorbaloochi S, Grill JP, Snyder A, et al. Validation of screening questions for limited health literacy in a large VA outpatient population. *J Gen Intern Med* 2008;23(5):561-6.
70. Herndon JB, Chaney M, Carden D. Health literacy and emergency department outcomes: a systematic review. *Annals of emergency medicine* 2011;57(4):334-45.
71. Drummond MF, Jefferson TO. Guidelines for authors and peer reviewers of economic submissions to the BMJ. The BMJ Economic Evaluation Working Party. *BMJ* 1996;313(7052):275-83.
72. Brooks R. EuroQol: the current state of play. *Health Policy* 1996;37(1):53-72.
73. Manca A, Hawkins N, Sculpher MJ. Estimating mean QALYs in trial-based cost-effectiveness analysis: the importance of controlling for baseline utility. *Health Econ* 2005;14(5):487-96.
74. Schulz KF, Altman DG, Moher D. CONSORT 2010 statement: updated guidelines for reporting parallel group randomized trials. *Ann Intern Med*;152(11):726-32.

Etiquette

Annex 1

Recueil de données
Equipe mobile vulnérabilitéS
DUMSC - CHUV

La santé est un état de complet bien-être physique, mental et social, et ne consiste pas seulement en une absence de maladie ou d'infirmité (OMS)

Nom

Prénom

Date de naissance (JJ/MM/AAAA)

IPP

Sexe : M ☐ F ☐

☐ Patient informé de la confidentialité de ce recueil et de ses limites

1er référent du patient : ☐ Intervenant 1 ☐ Intervenant 2 ☐ Intervenant 3 ☐ Autre intervenant:

2ème référent du patient : ☐ Intervenant 1 ☐ Intervenant 2 ☐ Intervenant 3 ☐ Autre intervenant:

Date d'ouverture du dossier EmvS (JJ/MM/AAAA) :

Date de fermeture du dossier EmvS (JJ/MM/AAAA) :

Mandant de l'intervention : ☐1 EmvS - dépistage ☐2 URG-CHUV ☐3 URG-PMU ☐4 Autre :

Nom, prénom, fonction et coordonnées du mandant :

DONNEES GENERALES DU PATIENT

Coordonnées du patient

Adresse :

Numéro de téléphone :

Représentant légal (tutelle/curatelle) (cf. liste des intervenants) ? oui ☐ non ☐

Le patient s'exprime

En français sans difficultés majeures ☐1

En français avec difficultés majeures ☐2

Dans une autre langue que le français ☐3

Laquelle ?

Nécessité d'un **traducteur (cf. liste des intervenants)** ? oui ☐1 non ☐2

Si nécessité d'avoir un traducteur mais impossibilité de l'organiser, pour quelle raison ?

☐1 indisponibilité ☐2 pas de traducteur dans la langue désirée ☐3 impossibilité de financer le service ☐4 autre raison

Liste des intervenants du réseau

**Nom, prénom et spécialité ou fonction**	**Coordonnées**
Nom :<br>Prénom :<br>Fonction :	Adresse :<br>Tél :<br>Fax :<br>E-mail :
Nom :<br>Prénom :<br>Fonction :	Adresse :<br>Tél :<br>Fax :<br>E-mail :
Nom :<br>Prénom :<br>Fonction :	Adresse :<br>Tél :<br>Fax :<br>E-mail :
Nom :<br>Prénom :<br>Fonction :	Adresse :<br>Tél :<br>Fax :<br>E-mail :
Nom :<br>Prénom :<br>Fonction :	Adresse :<br>Tél :<br>Fax :<br>E-mail :

Etiquette

Annex 1

1. A quelle fréquence avez-vous besoin de l'aide d'un tiers (membre de la famille, ami, personnel de la clinique ou de l'hôpital) pour lire des instructions, des informations ou d'autres documents médicaux ?

   - ☐ 0 Jamais
   - ☐ 1 Rarement
   - ☐ 2 De temps en temps
   - ☐ 3 Souvent
   - ☐ 4 Tout le temps

2. A quelle fréquence avez-vous des problèmes de compréhension sur votre situation de santé parce que vous avez des difficultés à la lecture ?

   - ☐ 0 Jamais
   - ☐ 1 Rarement
   - ☐ 2 De temps en temps
   - ☐ 3 Souvent
   - ☐ 4 Tout le temps

3. Est-ce que vous vous sentez sûr de vous lorsque vous remplissez des documents médicaux ?

   - ☐ 0 Jamais
   - ☐ 1 Rarement
   - ☐ 2 De temps en temps
   - ☐ 3 Souvent
   - ☐ 4 Tout le temps

Etiquette

Annex 2

DETERMINANTS SOMATIQUES

Motif(s) de consultation du patient aux urgences/autres services ou motif(s) d'hospitalisation :

Grossesse en cours ? oui ☐ non ☐ **Suivi gynéco-obst.** en cours **(cf. listes des intervenants)** : oui ☐ non ☐

Difficultés de mobilité (p.ex. handicap) **?** oui ☐ non ☐

Utilisation de moyens auxiliaires ? ☐ fauteuil roulant standard ou électrique ☐ cannes ☐ déambulateur (tintébin) ☐ autre :

Médication actuelle (y compris contraception)

☐ Aucun médicament ☐ médicaments multiples (> 5 médic.) ☐ médicaments coûteux

Médicaments relevants ou importants :

Compliance médicamenteuse douteuse ou mauvaise ? oui ☐ non ☐

Allergies connues ? oui ☐ non ☐ Si oui, à quoi ?

Déterminants somatiques

☐ Maladie/s aiguë/s ou chronique/s sévère/s[1]

☐ Polymorbidité somatique[2]

☐ Traitement médicamenteux complexe[3]

☐ Adhérences thérapeutique et/ou médicamenteuse inadéquates[4]

☐ Grossesse et/ou période néonatale[5]

☐ Mobilité restreinte/handicap physique[6]

Recueil des informations importantes

Diagnostics somatiques :

Notes/commentaires :

Objectif de prise en charge 1 (Ob soma 1) :

Description :

Mesures à entreprendre/objectifs :

Objectif de prise en charge 2 (Ob soma 2) :

Description :

Mesures à entreprendre/objectifs :

Objectif de prise en charge 3 (Ob soma 3) :

Description :

Mesures à entreprendre/objectifs :

Etiquette

Annex 2

DETERMINANTS DE SANTE MENTALE

Motif(s) de consultation du patient aux urgences/autres services ou motif(s) d'hospitalisation :

Etat de santé mentale

- ☐ Polymorbidité psychiatrique[1]
- ☐ Trouble de l'humeur (y.c. auto/hétéro-aggressivité, tentamen,…)[2]
- ☐ Trouble anxieux[3]
- ☐ Trouble psychotique[4]
- ☐ Trouble de la personnalité[5]
- ☐ Trouble somatoforme[6]
- ☐ Syndrome de stress post-traumatique[7]
- ☐ Démence[8]
- ☐ Troubles du développement psychologique (retard mental,…)[9]

Recueil des informations importantes

Diagnostics psychiatriques :

Notes/commentaires :

Objectif de prise en charge 1 (Ob psy 1) :

Description :<br><br>Mesures à entreprendre/objectifs :

Objectif de prise en charge 2 (Ob psy 2) :

Description :<br><br>Mesures à entreprendre/objectifs :

Objectif de prise en charge 3 (Ob psy 3) :

Description :<br><br>Mesures à entreprendre/objectifs :

Etiquette

Annex 2

DÉTERMINANTS COMPORTEMENTAUX

CONSOMMATIONS

Alcool ☐[1] Tabac ☐[2] Drogues ☐[3] :

Quantité (nb de paquet de cigarette/nb litre d'alcool/nb de joint/etc par jour ou mois) :

Soutien et suivi spécialisé déjà existant ? oui ☐ non ☐ Si **oui**, auprès de qui/quelle service ou institution ?

VIOLENCES INTER-PERSONNELLES

Patient est victime de **violences** physiques ou verbales ? oui ☐ non ☐

Si oui, des démarches ont-elles été entreprises ? oui ☐ non ☐ Si **oui**, auprès de qui/quelle institution ?

COMPORTEMENTS SEXUELS

Comportements sexuels à risques au cours des 12 derniers mois ? oui ☐ non ☐

Désir d'un dépistage pour les maladies sexuellement transmissibles ? oui ☐ non ☐

☐ Abus de substances/Dépendances (alcool, tabac, drogues, jeux, médicaments…)[1]

☐ Comportement sexuel à risque[2]

☐ Problématiques en lien avec la contraception ou l'interruption de grossesse[3]

☐ Violences inter-personnelles morales et/ou physiques (y.c. violences conjugales, mobbing, abus sexuels,...)[4]

☐ Situation à risque ou représentant un danger pour un enfant[5]

Recueil des informations importantes

Notes/commentaires :

Objectif de prise en charge 1 (Ob comp 1) :

Description :

Mesures à entreprendre/objectifs :

Objectif de prise en charge 2 (Ob comp 2) :

Description :

Mesures à entreprendre/objectifs :

Objectif de prise en charge 3 (Ob comp 3) :

Description :

Mesures à entreprendre/objectifs :

Etiquette

Annex 2

DETERMINANTS CONSOMMATION DE SOINS

Usager fréquent des urgences (« frequent user » ≥ 5 visites/an) ? oui ☐ non ☐

Combien de visites au cours des 12 derniers mois (lors de la première évaluation par l'EmvS) ?

Consommation de soins

☐ Recours fréquents au service des urgences CHUV-PMU ou à d'autres lieux de soins[1]

☐ Multiples intervenants médico-infirmiers[2]

☐ Aucun médecin de premier recours extra-hospitalier[3]

☐ Difficultés dans la relation aux soignants[4]

Recueil des informations importantes

Notes/commentaires :

Objectif de prise en charge 1 (Ob soins 1) :

Description :

Mesures à entreprendre/objectifs :

Objectif de prise en charge 2 (Ob soins 2) :

Description :

Mesures à entreprendre/objectifs :

Objectif de prise en charge 3 (Ob soins 3) :

Description :

Mesures à entreprendre/objectifs :

Etiquette

Notes de suite

Date	Objectif	Sign	Notes

Etiquette

Annex 2

FACTURATION EmvS

Nombres d'entretiens effectués avec le patient par intervenant(s) *(cocher les cases)*

	5x · 10x · 15x · 20x · 30x
Oliver Collis[1]	☐☐☐☐☐☐☐☐☐☐☐☐☐☐☐☐☐☐☐☐☐☐☐☐☐☐☐☐☐☐
Marina Canepa Allen[2]	☐☐☐☐☐☐☐☐☐☐☐☐☐☐☐☐☐☐☐☐☐☐☐☐☐☐☐☐☐☐
Corine Ansermet[3]	☐☐☐☐☐☐☐☐☐☐☐☐☐☐☐☐☐☐☐☐☐☐☐☐☐☐☐☐☐☐
Francis Vu[4]	☐☐☐☐☐☐☐☐☐☐☐☐☐☐☐☐☐☐☐☐☐☐☐☐☐☐☐☐☐☐
Binôme assistant social – infirmière[5]	☐☐☐☐☐☐☐☐☐☐☐☐☐☐☐☐☐☐☐☐☐☐☐☐☐☐☐☐☐☐
Binôme médecin – assistant social[6]	☐☐☐☐☐☐☐☐☐☐☐☐☐☐☐☐☐☐☐☐☐☐☐☐☐☐☐☐☐☐
Binôme médecin - infirmière[7]	☐☐☐☐☐☐☐☐☐☐☐☐☐☐☐☐☐☐☐☐☐☐☐☐☐☐☐☐☐☐
Autre[8]	☐☐☐☐☐☐☐☐☐☐☐☐☐☐☐☐☐☐☐☐☐☐☐☐☐☐☐☐☐☐

Durées des interventions, par 5 min. *(cocher les cases)*

1. entretiens **individuels en présence** du patient[1]

30' 60' 90' 120' 150' 180' 210'

☐☐

☐☐

240' 270' 300' 330' 360' 390' 420'

2. évaluation **globale en l'absence** du patient (y compris étude du dossier)[2]

30' 60' 90' 120' 150' 180' 210'

☐☐

☐☐

240' 270' 300' 330' 360' 390' 420'

3. entretiens de **réseau en présence** du patient[3]

30' 60' 90' 120' 150' 180' 210'

☐☐

☐☐

240' 270' 300' 330' 360' 390' 420'

4. entretiens de **réseau en l'absence** du patient[4]

30' 60' 90' 120' 150' 180' 210'

☐☐

☐☐

240' 270' 300' 330' 360' 390' 420'

5. entretiens **téléphoniques avec le patient**[5]

30' 60' 90' 120' 150' 180' 210'

☐☐

☐☐

240' 270' 300' 330' 360' 390' 420'

Etiquette

Annex 2

Feedback des situations prises en charge par l'EmvS à 3 mois

☐[1] Intervenant 1 ☐[2] Intervenant 2 ☐[3] Intervenant 3 ☐[4] Autre intervenant :

Date :

Feedback pris auprès du :

☐[1] patient ☐[2] médecin traitant ☐[3] assistant social ☐[4] infirmier/soignant ☐[5] famille/entourage proche

Qui (préciser) ?

Déterminants somatiques :

Etat de santé mental :

Déterminants comportementaux :

Déterminants sociaux :

Consommation des soins :

Feedback des situations prises en charge par l'EmvS à 6 mois

☐[1] Intervenant 1 ☐[2] Intervenant 2 ☐[3] Intervenant 3 ☐[4] Autre intervenant :

Date :

Feedback pris auprès du :

☐[1] patient ☐[2] médecin traitant ☐[3] assistant social ☐[4] infirmier/soignant ☐[5] famille/entourage proche

Qui (préciser) ?

Déterminants somatiques :

Etat de santé mental :

Déterminants comportementaux :

Déterminants sociaux :

Consommation des soins :

Etiquette

6. entretiens **téléphoniques avec le réseau**[6]

30' 60' 90' 120' 150' 180' 210'

240' 270' 300' 330' 360' 390' 420'

Traductions (durée de l'intervention du traducteur), par 5 min. *(cocher les cases)*

☐[1] traduction par entourage/famille ☐[2] traducteur professionnel ☐[3] traducteur CHUV ☐[4] autre :

30' 60' 90' 120' 150' 180' 210'

240' 270' 300' 330' 360' 390' 420'

Présentation du cas lors des colloques hebdomadaires EmvS, par 5 min. *(cocher les cases)*

30' 60' 90' 120' 150' 180' 210'

240' 270' 300' 330' 360' 390' 420'

Rédaction de lettres, rapports ou e-mails, par 5 min. *(cocher les cases)*

30' 60' 90' 120' 150' 180' 210'

240' 270' 300' 330' 360' 390' 420'

Rendez-vous manqué(s) par le patient *(cocher les cases)*

5x 10x 15x 20x 30x 40x

Déplacements (indemnités), par km *(cocher les cases)*

5x 10x 15x 20x 30x 40x

Etiquette

Equipe mobile vulnérabilités - EmvS
Nes_03_3003
Avenue Pierre-Decker 5
CH-1011 Lausanne

Madame la Dresse
xxxx
Consultation Générale – PMU
Rue du Bugnon 44
1011 Lausanne

Madame P, née
N°IPP

Lausanne, le 22.07.2010

Madame et Chère Consoeur,

Le/la patient(e) susnommé(e) a été évalué(e) par l'Equipe mobile vulnérabilitéS (EmvS) dans la période du **4 juillet 2010** au **22.07.2010.**

DEMANDEUR DE L'INTERVENTION
Dépistage EmvS par Mme xxxx, psychologue, via l'alarme « Usagers Fréquents » du programme Gyropat des Urgences CHUV, en date du **4 juillet 2010.**

EVALUATION SELON LES 5 AXES DE VULNERABILITE
La demande d'intervention de l'équipe EmvS a été motivée par la constatation d'un cumul de **8 *critères de vulnérabilités*** présents dans les 5 axes de vulnérabilités suivants :

1) DETERMINANTS SOMATIQUES

- ☒ Maladie/s aiguë/s ou chronique/s sévère/s
- ☐ Traitement médicamenteux complexe
- ☐ Grossesse et/ou période néonatale
- ☐ Polymorbidité somatique
- ☐ Adhérences thérapeutique et/ou médicamenteuses inadéquates
- ☐ Mobilité restreinte/handicap physique

2) ETAT DE SANTE MENTALE

- ☐ Polymorbidité psychiatrique
- ☐ Trouble anxieux
- ☐ Trouble psychotique
- ☐ Trouble somatoforme
- ☐ Démence
- ☐ Trouble de l'humeur (y.c. autoaggressivité, tentamen,…)
- ☐ Trouble de la personnalité
- ☐ Syndrome de stress post-traumatique
- ☐ Troubles du développement psychologique

3) DETERMINANTS COMPORTEMENTAUX

- ☐ Abus de substances/dépendances actives (alcool, tabac, drogues, médicaments, jeux,…)
- ☐ Comportement sexuel à risque
- ☐ Problématiques en lien avec la contraception ou l'interruption de grossesse
- ☐ Situation à risque ou représentant un danger pour un enfant
- ☐ Violences inter-personnelles morales et/ou physiques (y.c. violences conjugales, mobbing, abus sexuels,...)

Etiquette

4) DETERMINANTS SOCIAUX

- ☒ Situation familiale complexe/difficile
- ☒ Situation financière complexe/difficile
- ☐ Assurance inexistante ou insuffisante
- ☒ Origine et/ou statut de séjour précaire
- ☒ Exclusion sociale ou isolement social
- ☒ Logement inadéquat ou inexistant
- ☐ Difficultés ou absence au travail/écoles/activités sociales
- ☒ Difficultés de compréhension/maîtrise insuffisante d'une langue commune

5) CONSOMMATION DE SOINS

- ☐ Recours fréquents au service des urgences CHUV-PMU ou à d'autres lieux de soins
- ☐ Multiples intervenants médico-infirmiers
- ☒ Aucun médecin de premier recours extra-hospitalier
- ☐ Difficultés dans la relation aux soignants

ORIENTATION ET DEMARCHES ENTREPRISES

1) **DETERMINANTS SOMATIQUES :** Mme P présente de longue date des ***céphalées diffuses*** de type tensionnelles qui se sont nettement aggravées en novembre 2009, avec l'apparition de vertiges, de troubles de l'équilibre, de vomissements et de paresthésies au membre supérieur gauche. Le bilan de ces troubles neurologiques effectué au CHUV a mis en évidence un ***kyste épidermoïde centré sur l'angle ponto-cérébelleux droit***. En juin 2010, la patiente a bénéficié d'une excision de ce kyste par nos collègues neurochirurgiens du CHUV, ***compliquée d'une pseudo-méningocèle*** dans les suites opératoires. En raison de l'apparition de symptômes méningés et suspicion de méningite, Mme P est hospitalisée depuis le 13 juillet 2010 en neurochirurgie – CHUV.

2) **ETAT DE SANTE MENTAL :** en dépit de sa précarité sociale (cf. « déterminants sociaux ») et de ses problèmes somatiques, Mme P ne décrit actuellement ***pas de trouble de l'humeur*** ni de crise d'angoisses significatifs. Il nous paraît toutefois indispensable de ***réévaluer régulièrement la thymie de la patiente***, son contexte de vie actuellement difficile pouvant conduire à un effondrement de son humeur. A noter que le contact avec la patiente est bon et nous ne relevons pas de bizarrerie dans le comportement ni de symptômes francs de la lignée psychotique.

3) **DETERMINANTS COMPORTEMENTAUX :** ***pas de*** consommation de substances psycho-actives. ***Pas de*** consommation d'alcool et reprise occasionnelle de la consommation de tabac depuis quelques jours. Ancienne « petite » fumeuse, avait arrêté le tabac 20 jours avant son opération.

4) **DETERMINANTS SOCIAUX :** ***Originaire de l'Equateur***, Mme P est en Suisse dans la ***clandestinité depuis environ 10 ans***. Mère de ***3 enfants*** (âgés respectivement de 21, 24 et 26 ans) restés en Equateur, ***séparée*** du père depuis 12 ans, la patiente a vécu chez Les « Soeurs de la Charité » de décembre 2009 à juillet 2010, et vit actuellement chez sa belle-sœur. A l'heure actuelle, au vu de sa grande précarité, de l'altération de son état général en lien avec ses problèmes de santé somatiques et de l'impossibilité d'exercer une profession qui en découle, Mme P ***envisage de rentrer dans son pays d'origine*** afin d'y retrouver ses enfants. Afin d'accéder à sa demande, un accompagnement au ***bureau d'Aide au retour*** à Lausanne sera effectué par Mme xxxx le 20 août prochain. La patiente bénéficie également d'un ***soutien par le Service social du CHUV*** (Mme xxxx).

Etiquette

5) **CONSOMMATION DE SOINS :** en l'absence de médecin de premier recours jusqu'alors, la patiente a été adressée à votre ***consultation générale à la PMU*** le 12.07.2010 pour un suivi au long cours.

OBJECTIFS ET PROPOSITIONS

- Suivi médical conjoint par le Service de Neurochirurgie du CHUV et la Consultation Générale de la PMU
- Soutien par le Service Social du CHUV
- Accompagnement pour l'aide au retour au pays d'origine de la patiente (Equateur)

RESEAU D'INTERVENANTS

Dresse xxxx<br>Médecin-assistant	Consultation Générale – PMU<br>Rue du Bugnon 44<br>1011 Lausanne	Tél. xxxx
Dr xxxx<br>Chef de clinique adjoint	Centre Universitaire Romand<br>De Neurochirurgie<br>CHUV – 1011 Lausanne	Tél. xxxx
Mme xxxx<br>Assistante sociale	Service Social CHUV<br>1011 Lausanne	Tél. xxxx
Mme xxxx<br>Assistante sociale	Service d'Aide au Retour<br>SPOP<br>Av. de Beaulieu 25<br>1014 Lausanne	Tél. xxxx

En restant à votre disposition pour tout renseignement complémentaire, nous vous prions d'agréer, Monsieur et Cher Confrère, nos meilleures salutations.

Signatures

Intervenant 1 Intervenant 2 Intervenant 3

INFORMATION AU PATIENT

Prise en charge de type « gestion de cas » *(case management)* des usagers fréquents des services d'urgence : un essai clinique randomisé.

Département Universitaire de Médecine et Santé Communautaire du CHUV et Policlinique Médicale Universitaire.

Madame, Monsieur,

Nous vous proposons de participer à cette étude, parce que vous êtes un usager fréquent des urgences, c'est-à-dire que vous avez consulté le secteur des urgences 5 fois ou plus au cours des 12 derniers mois.
Cette étude a pour but d'étudier l'impact d'une intervention, réalisée par un binôme interdisciplinaire composé d'une infirmière et d'une psychologue, auprès des patients usagers fréquents des urgences, en plus de l'intervention habituelle des médecins et infirmières/ers.
L'intervention de ce binôme devrait permettre à terme :

- une diminution du recours au secteur des urgences par les usagers fréquents (parce que mieux orientés dans le service des soins) ;
- une augmentation de leur qualité de vie au cours de l'année de suivi ;
- une diminution globale des coûts de la santé par une meilleure orientation au sein du système de soins des usagers fréquents des urgences.

En effet, les patients usagers fréquents des urgences sont souvent considérés comme plus vulnérables de par leurs caractéristiques et demandes socio-médicales, et une prise en charge par une équipe interdisciplinaire est décrite dans la littérature comme étant plus adaptée à cet état de vulnérabilité.
Cette étude, financée par le Fonds National Suisse de la Recherche (FNSR), est une étude locale qui sera réalisée durant la période allant d'avril 2012 à avril 2014 (période d'inclusion d'une année, puis période de suivi d'une année). Parmi l'ensemble des patients usagers fréquents du secteur des urgences consultant durant cette période, 140 d'entre eux tirés au hasard bénéficieront en plus de la prise en charge habituelle, d'une intervention interdisciplinaire par une infirmière et une psychologue, alors que 140 autres patients tirés au hasard bénéficieront d'une prise en charge habituelle par l'équipe des urgences. Cette étude est réalisée conformément aux lois suisses en vigueur et dans le respect de principes reconnus au plan international. Le protocole de cette étude de recherche a reçu l'avis positif de la Commission d'éthique de la recherche du ...., en date du...../...../2012.

Description de l'étude
Il s'agit d'un essai clinique randomisé dans lequel les patients sont assignés au hasard (comme si on tirait à pile ou face une pièce de monnaie) au groupe intervention ou au groupe contrôle. Cet essai clinique se fait en simple aveugle, c'est-à-dire que le patient ne sait pas de quelle intervention il bénéficiera. La durée du suivi est de un an à partir du jour de l'inclusion (figure 1).

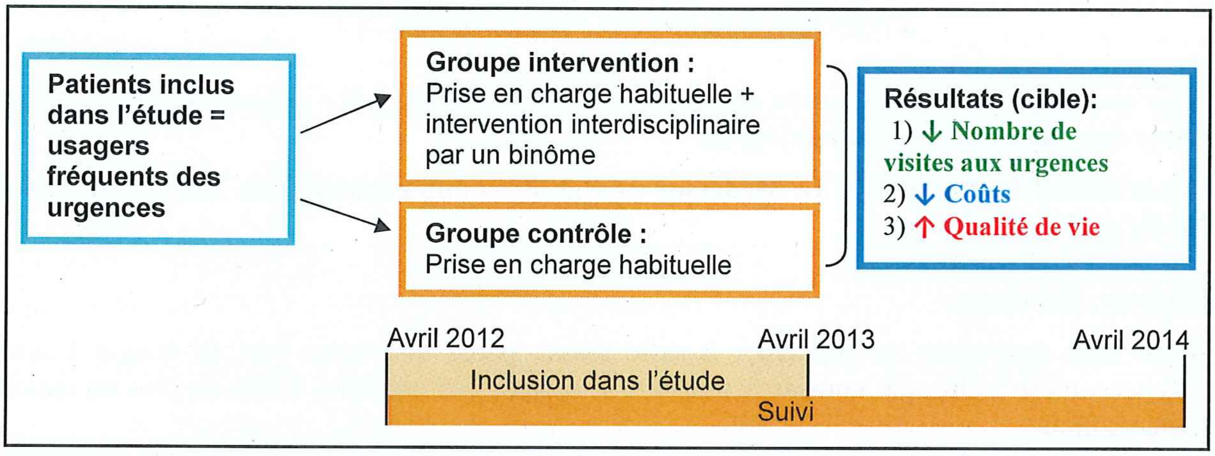

Figure 1 : description de l'étude

Au cours du suivi un questionnaire sur votre recours au système de soins, les coûts de vos différentes consultations, ainsi que sur votre qualité de vie, vous sera soumis par une infirmière de recherche à 3, 6, 9 et 12 mois, quel que soit le groupe (intervention ou contrôle) auquel vous appartiendrez ; un recours de votre part au binôme interdisciplinaire sera possible à tout moment de l'étude.

Votre participation à l'étude

Votre participation à cette étude est volontaire. Renoncer à y prendre part n'aura aucune incidence sur votre suivi médical ultérieur. Le même principe s'applique en cas de révocation de votre consentement initial. Vous pouvez donc renoncer en tout temps à votre participation. Vous n'êtes tenu(e) de justifier ni la révocation de votre consentement, ni un désistement éventuel. En cas de révocation, les données recueillies jusqu'alors continueront toutefois à être utilisées.
En tant que participant(e) à cette étude, vous êtes tenu(e) de suivre les instructions médicales de votre médecin investigateur et de vous conformer au plan de l'étude.
Si vous ne souhaitez pas participer à cette étude, vous disposez des possibilités de prise en charge habituelle par les médecins et infirmiers/ères des services des urgences.
Participer à cette étude, pourrait vous procurer des avantages tels qu'une meilleure orientation au sein du système de soins, la mise en place d'un réseau ou la réactivation de ce réseau, et un suivi par le binôme infirmière/psychologue pendant une année. Votre participation peut par ailleurs permettre à d'autres personnes de profiter des résultats de cette étude.
Il n'y a aucun risque ou désagrément induit par la participation à l'étude.

Informations supplémentaires

Le médecin investigateur vous informera de toute découverte qui pourrait avoir de l'importance pour votre santé ou qui pourrait modifier le bon déroulement de la suite de l'étude, et donc influencer votre consentement à poursuivre l'étude. Ces informations vous seront communiquées par écrit.

Anonymisation de vos données

Des données personnelles vous concernant seront recueillies pendant l'étude. Elles sont toutefois rendues anonymes et ne sont accessibles qu'à des spécialistes à des fins d'analyses scientifiques. La Commission d'éthique compétente pourrait être amenée à consulter ces données. Le promoteur en Suisse ou le représentant du promoteur étranger en Suisse répond du respect des dispositions nationales et internationales relatives à la protection des données.

Coûts et rétribution

Les interventions réalisées par le binôme infirmière/psychologue sont financées en partie par le FNSR. L'intervention de l'EmvS sera prise en charge par votre assurance.

Contacts

Si, pendant ou à l'issue de l'essai clinique, vous deviez souffrir de problèmes de santé ou constatez des dommages d'une autre nature, veuillez vous adresser au médecin compétent (ci-dessous) qui engagera pour vous la procédure requise.

En cas d'urgence, d'incertitude ou d'événement inattendu ou indésirable survenant pendant ou après l'essai clinique, vous pouvez vous adresser à tout moment à la personne suivante :

Médecin investigateur, Dr Patrick Bodenmann, Tél 079/556 44 67

Service des urgences CHUV, Bugnon 44, 1011 Lausanne

Service des urgences PMU, Bugnon 44, 1011 Lausanne

CONSENTEMENT ÉCLAIRÉ ÉCRIT DU PATIENT POUR LA PARTICIPATION À UNE ÉTUDE CLINIQUE

- Veuillez lire attentivement ce formulaire.
- N'hésitez pas à poser des questions si certains aspects vous semblent peu clairs ou si vous souhaitez obtenir des précisions.

Numéro de l'étude:	
Titre de l'étude:	Prise en charge de type «gestion de cas » *(case management)* des usagers fréquents des services d'urgence : un essai clinique randomisé
Promoteur (adresse complète) :	DUMSC/CHUV
Lieu de réalisation de l'essai clinique:	Service des Urgences, CHUV<br>Secteur des urgences ambulatoires, PMU
**Médecin-investigateur**<br>Nom et prénom :	Dr Patrick Bodenmann
**Patient(e)**<br>Nom et prénom :<br>Date de naissance :	☐ homme ☐ femme

- Je déclare avoir été informé(e), oralement et par écrit, par le soignant de l'Equipe mobile vulnérabilitéS (EmvS) des objectifs et du déroulement de l'étude, des avantages et des inconvénients possibles ainsi que des risques éventuels.
- Je certifie avoir lu et compris l'information écrite aux patients qui m'a été remise sur l'étude précitée, datée du.... J'ai reçu des réponses satisfaisantes aux questions que j'ai posées en relation avec ma participation à cet essai clinique. Je conserve l'information écrite aux patients et reçois une copie de ma déclaration écrite de consentement.
- J'ai été informé(e) de l'existence possible d'autres traitements.
- J'ai eu suffisamment de temps pour prendre ma décision.
- Je suis informé(e) qu'une assurance a été souscrite pour couvrir les dommages éventuels découlant de l'étude.
- Je sais que mes données personnelles ne seront transmises que sous une forme anonyme à des institutions externes à des fins de recherche. J'accepte que les spécialistes compétents du mandataire de l'étude, des autorités et de la Commission d'éthique cantonale puissent consulter mes données brutes, afin de procéder à des examens et à des contrôles, à condition toutefois que leur confidentialité soit strictement assurée.
- Je prends part de façon volontaire à cette étude. Je peux, à tout moment et sans avoir à fournir de justification, révoquer mon consentement à participer à cette étude, sans pour cela en subir quelque inconvénient que ce soit dans mon suivi médical ultérieur.
- Je suis conscient(e) du fait que les exigences et les restrictions mentionnées dans l'information aux patients devront être respectées pendant la durée de l'étude. Le soignant de l'Equipe mobile vulnérabilitéS (EmvS) peut m'exclure à tout moment de l'étude dans l'intérêt de ma santé. De mon côté, je m'engage à informer le soignant de l'Equipe mobile vulnérabilitéS (EmvS) de tout traitement concomitant auprès d'un autre médecin.

Lieu, date	Signature du patient/de la patiente

Attestation du soignant de l'Equipe mobile vulnérabilitéS (EmvS): J'atteste par ma signature avoir expliqué à ce/cette patient/e la nature, l'importance et la portée de l'étude. Je déclare satisfaire à toutes les obligations en relation avec cet essai clinique. Si je devais prendre connaissance, à quelque moment que ce soit durant la réalisation de l'étude, d'informations susceptibles d'influer sur le consentement du/de la patient(e) à participer à l'étude, je m'engage à l'en informer immédiatement.

Lieu, date	Signature du soignant de l'Equipe mobile vulnérabilitéS (EmvS)

24.06.2013

canton de vaud
LIBERTÉ ET PATRIE

Commission cantonale d'éthique de la recherche sur l'être humain
Av. de Chailly 23, 1012 Lausanne

Prof. R. Darioli, Président
Secrétariat central
Tél. 021 316 18 30/31/32/33
Fax 021 316 18 37
E-mail: secretariat.cer@vd.ch

Yes!
Patrick

Dr Patrick Bodenmann, MER, MSc
Médecin associé
PMU
Bugnon 44
1011 Lausanne

Sous-Commission II, Président Prof. R. Darioli
Tél. 021 316 18 35

Lausanne, le 19 juin 2013
RD/ns

Protocole 32/12 : Prise en charge de type "gestion de cas" (case-management) des patients usagers fréquents des services d'urgence: essai clinique randomisé

Amendement 1 : Avis de la CER-VD

Monsieur et cher Collègue,

La Commission a procédé à l'évaluation de l'amendement 1 au protocole susmentionné.

Cet avis est fondé sur l'examen des documents reçus le 17 juin 2013 :

1. Votre email du 14.06.2013
2. Version abrégée du protocole
3. Lettre patient du 15.06.2013
4. Feuille d'information
5. Formulaire de consentement
6. Addendum au consentement

Type de procédure:

☐ procédure ordinaire ☐ ré-évaluation ☐ procédure ordinaire CED

☐ procédure simplifiée ☒ Avis présidentiel ☐ Avis présidentiel CEL

La Commission arrête l'avis suivant:

☒ **positif**[1]

☐ **avis conditionnel**[2] (conditions à remplir avant approbation)

- ☐ Les documents révisés seront réévalués en procédure ordinaire (nombre de copies: 13)
- ☐ Révision des documents et information écrite à la Commission d'éthique (nombre de copies: 1)
- ☐ Entretien avec la Commission

☐ **négatif**[3] (motivé)

☐ **avis justifié de ne pas entrer en matière**[4]

....

signifie

[1] L'amendement peut être soumis aux autorités fédérales compétentes (Swissmedic / OFSP / OFEFP) pour notification, s'il s'agit d'un essai thérapeutique ou d'un essai avec dispositif médical. Dans les autres types d'étude, l'amendement peut être mis en application dès le présent avis positif.

[2] Les documents concernés doivent être révisés avant soumission à la Commission d'éthique.
L'amendement ne pourra prendre effet, ni être notifié avant d'avoir obtenu l'avis positif de la Commission d'éthique.

[3] Dans sa forme actuelle, l'amendement ne peut pas être effectif.

Emoluments perçus pour chaque amendement soumis à la Commission pour évaluation, selon barème ci-joint: **CHF 300.- (code 4.2).** Une facture **(n°32/12190613)** vous sera envoyée ultérieurement.

Remarques :

- La CER atteste qu'elle accomplit son travail conformément aux recommandations ICH-GCP.
- Conformément à l'art. 21 de l'Ordonnance sur les essais cliniques de produits thérapeutiques (OClin) et à l'art. 11 du Règlement de la Commission cantonale (VD) d'éthique de la recherche sur l'être humain, veuillez SVP retourner à la CER le rapport intermédiaire une fois par année puis le rapport final (cf pages 3-4).
- Droit de récours dans le cadre de la Commission d'éthique.
- L'avis s'applique également aux autres investigateurs(trices) mentionné(e)s dans la demande d'évaluation qui travaillant dans des sites de recherche relevant du champ de compétence de la CER (doivent figurer sur une liste séparée).

R. Darioli

Prof. Roger Darioli
Président de la Sous-Commission II
